# Supplementary figures and images for: PPM1G dephosphorylates eIF4E in control of mRNA translation and cell proliferation (part 1 of 2)
Source: Life Sci Alliance. 2024 Aug 7;7(10):e202402755. doi: 10.26508/lsa.202402755 (PMC11306785; doi:10.26508/lsa.202402755)

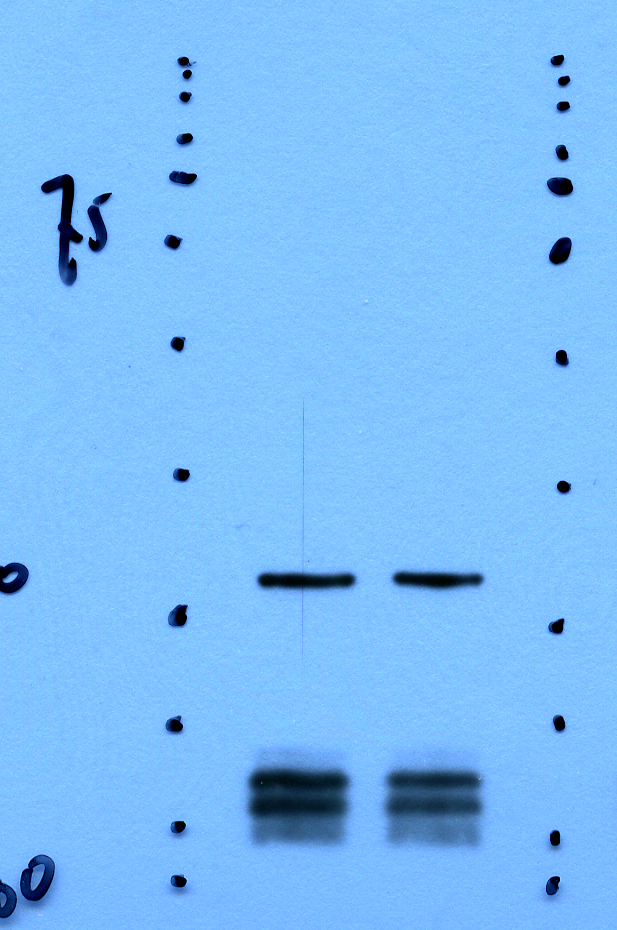

Supplement: Supplementary file 1 [file LSA-2024-02755_SdataF1.zip › LSA-2024-02755_SdataF1.1.tif]

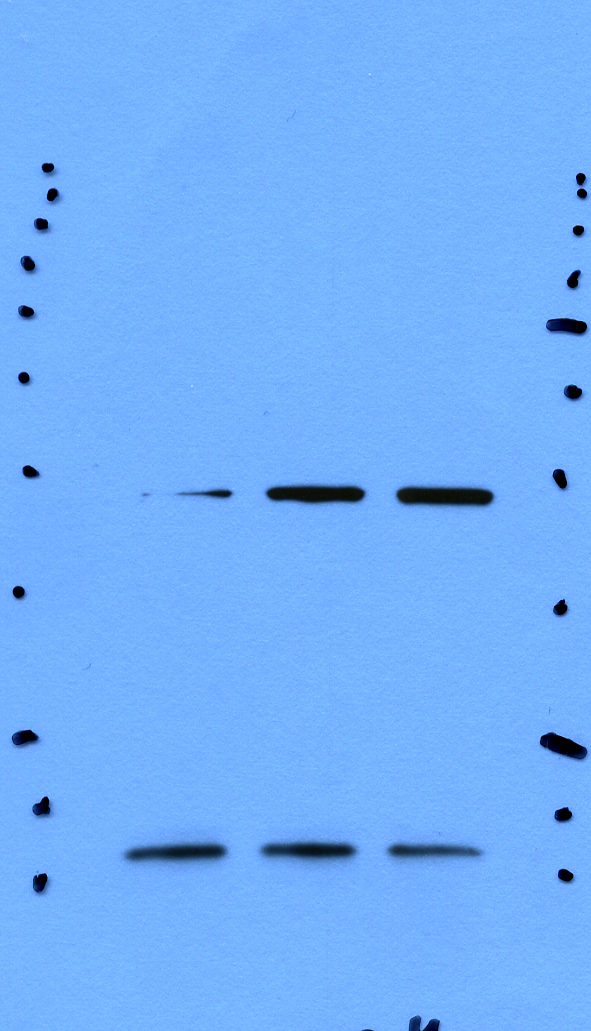

Supplement: Supplementary file 1 [file LSA-2024-02755_SdataF1.zip › LSA-2024-02755_SdataF1.10.tif]

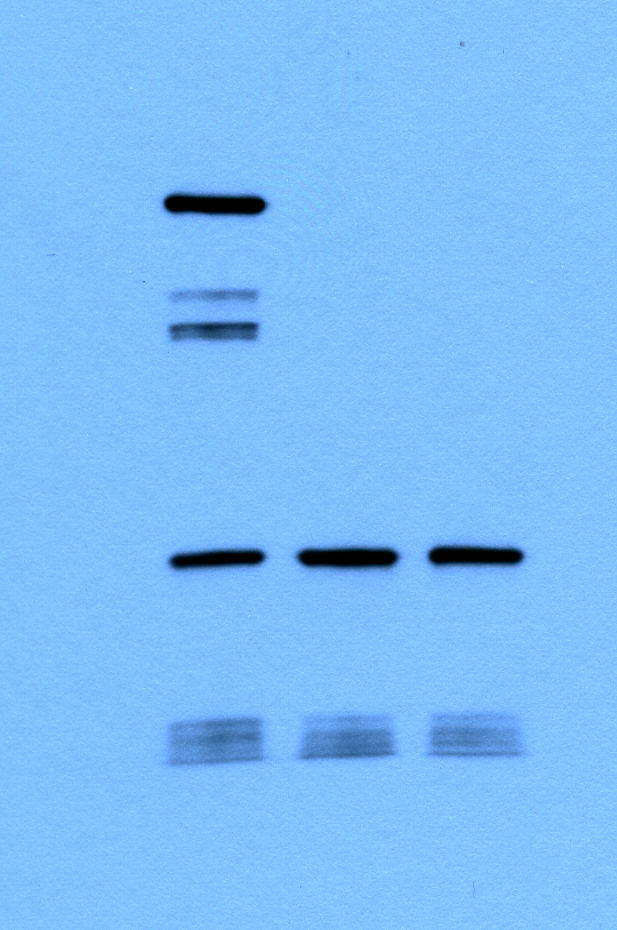

Supplement: Supplementary file 1 [file LSA-2024-02755_SdataF1.zip › LSA-2024-02755_SdataF1.11.tif]

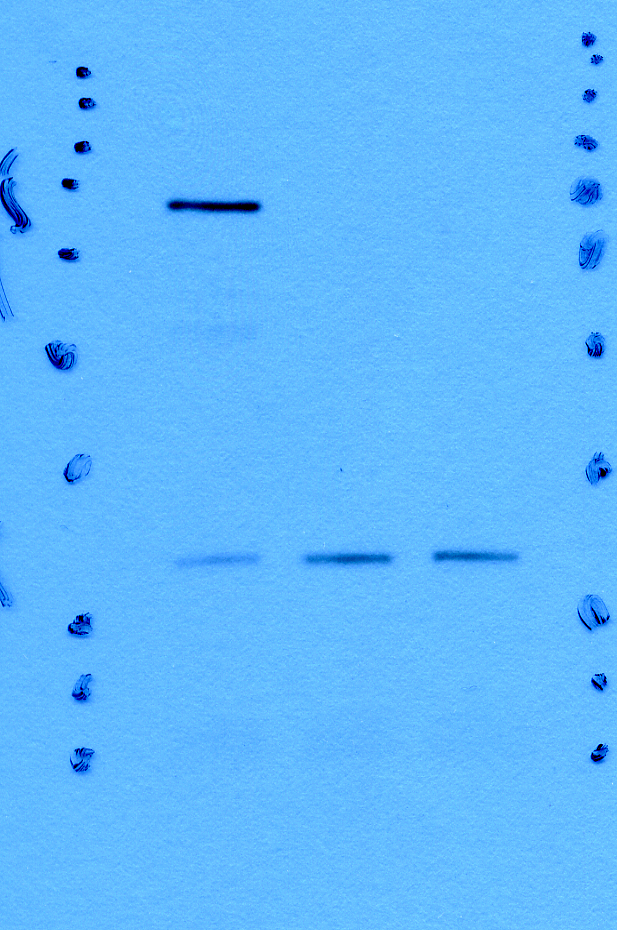

Supplement: Supplementary file 1 [file LSA-2024-02755_SdataF1.zip › LSA-2024-02755_SdataF1.12.tif]

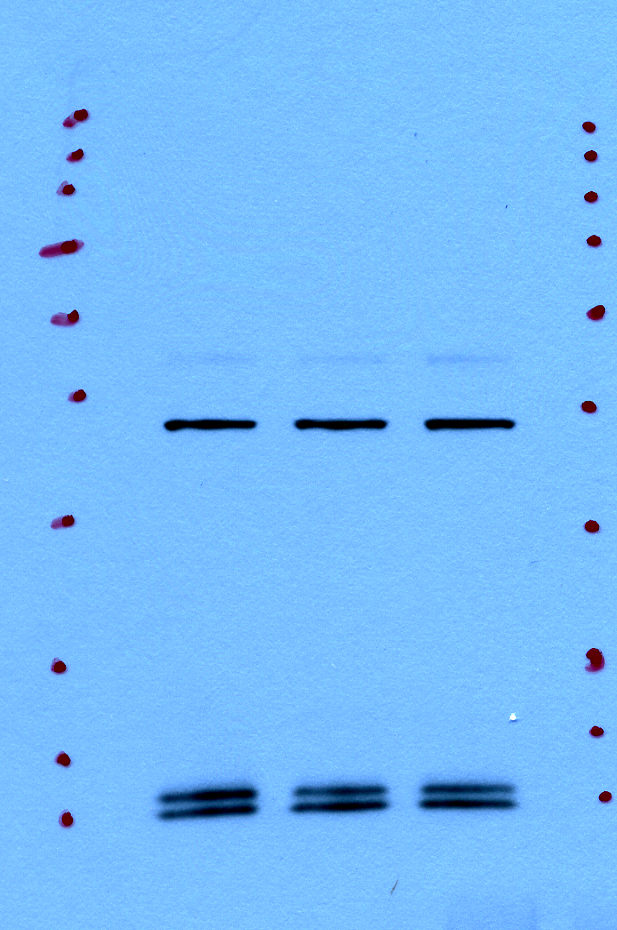

Supplement: Supplementary file 1 [file LSA-2024-02755_SdataF1.zip › LSA-2024-02755_SdataF1.13.tif]

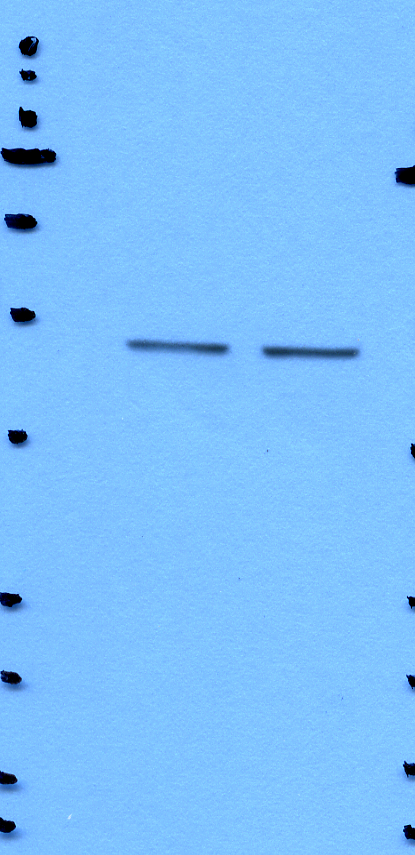

Supplement: Supplementary file 1 [file LSA-2024-02755_SdataF1.zip › LSA-2024-02755_SdataF1.14.tif]

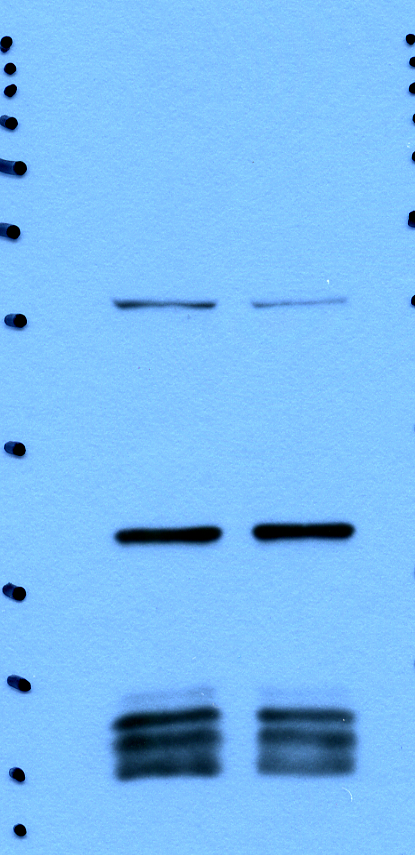

Supplement: Supplementary file 1 [file LSA-2024-02755_SdataF1.zip › LSA-2024-02755_SdataF1.15.tif]

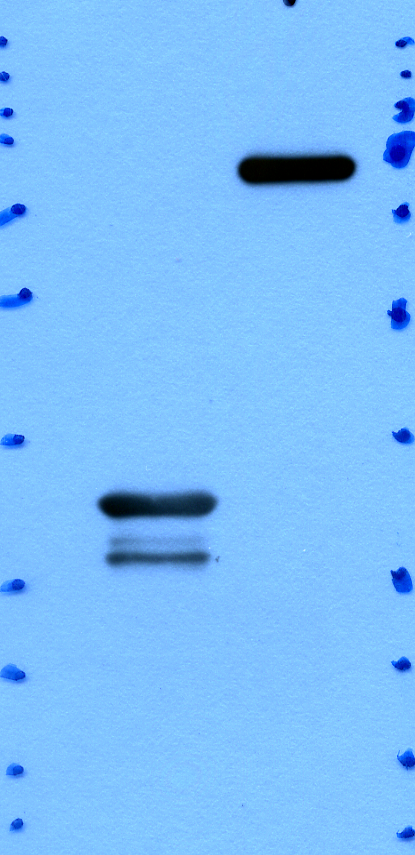

Supplement: Supplementary file 1 [file LSA-2024-02755_SdataF1.zip › LSA-2024-02755_SdataF1.16.tif]

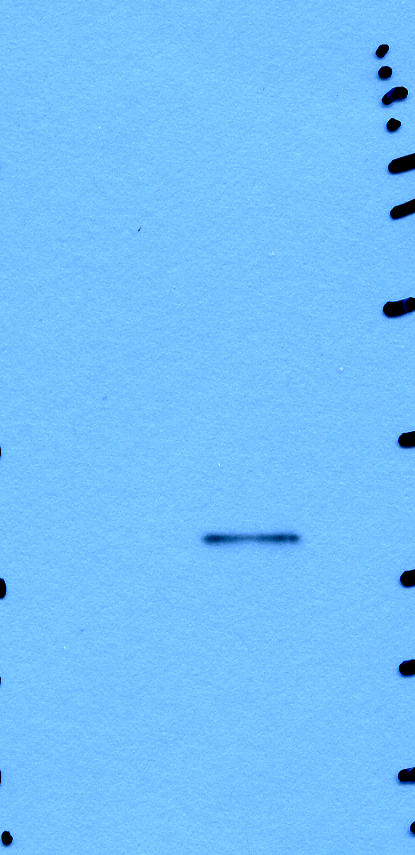

Supplement: Supplementary file 1 [file LSA-2024-02755_SdataF1.zip › LSA-2024-02755_SdataF1.17.tif]

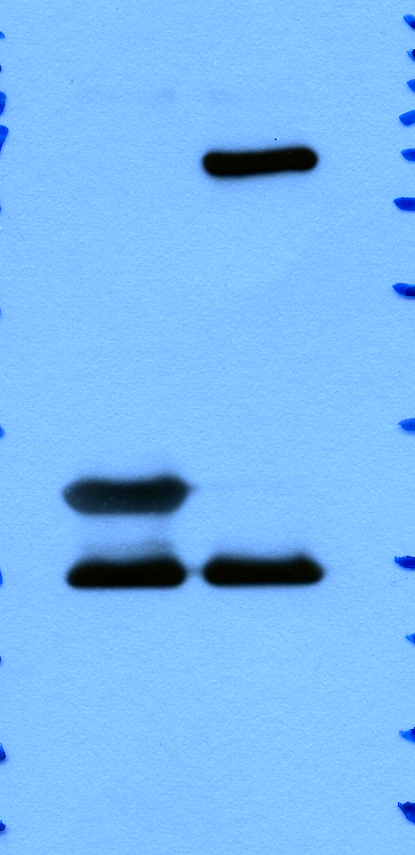

Supplement: Supplementary file 1 [file LSA-2024-02755_SdataF1.zip › LSA-2024-02755_SdataF1.18.tif]

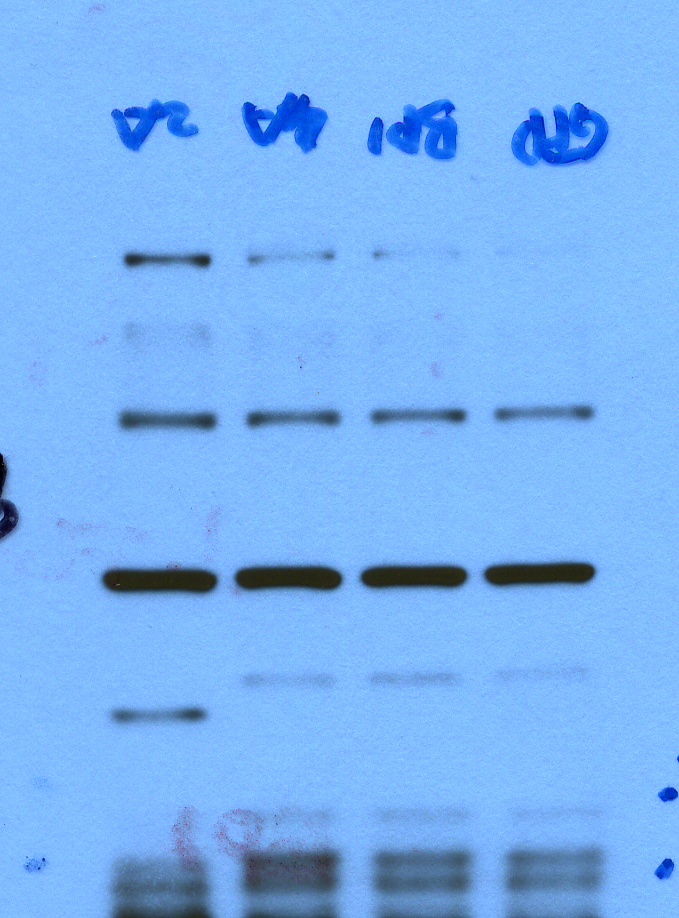

Supplement: Supplementary file 1 [file LSA-2024-02755_SdataF1.zip › LSA-2024-02755_SdataF1.19.tif]

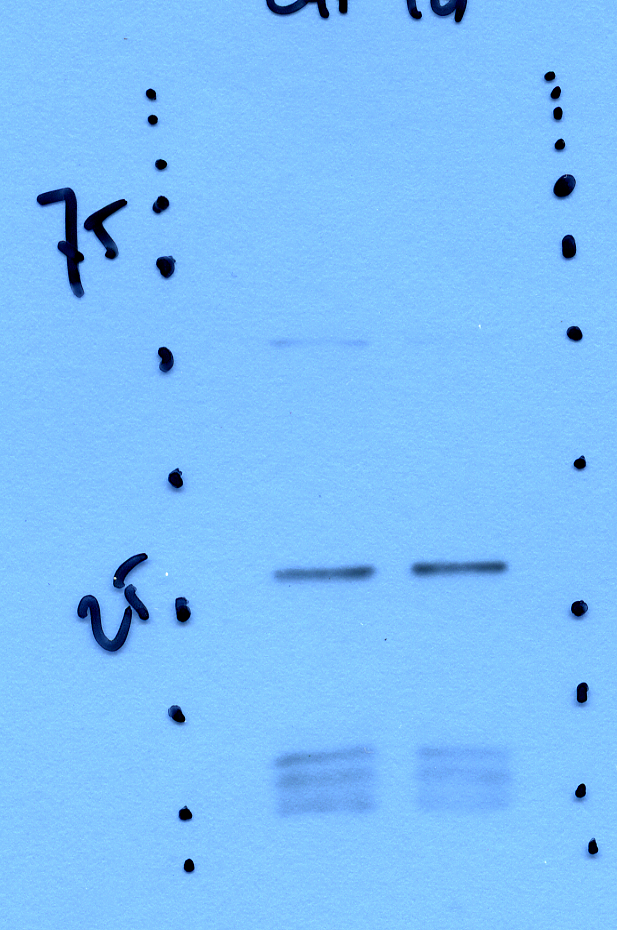

Supplement: Supplementary file 1 [file LSA-2024-02755_SdataF1.zip › LSA-2024-02755_SdataF1.2.tif]

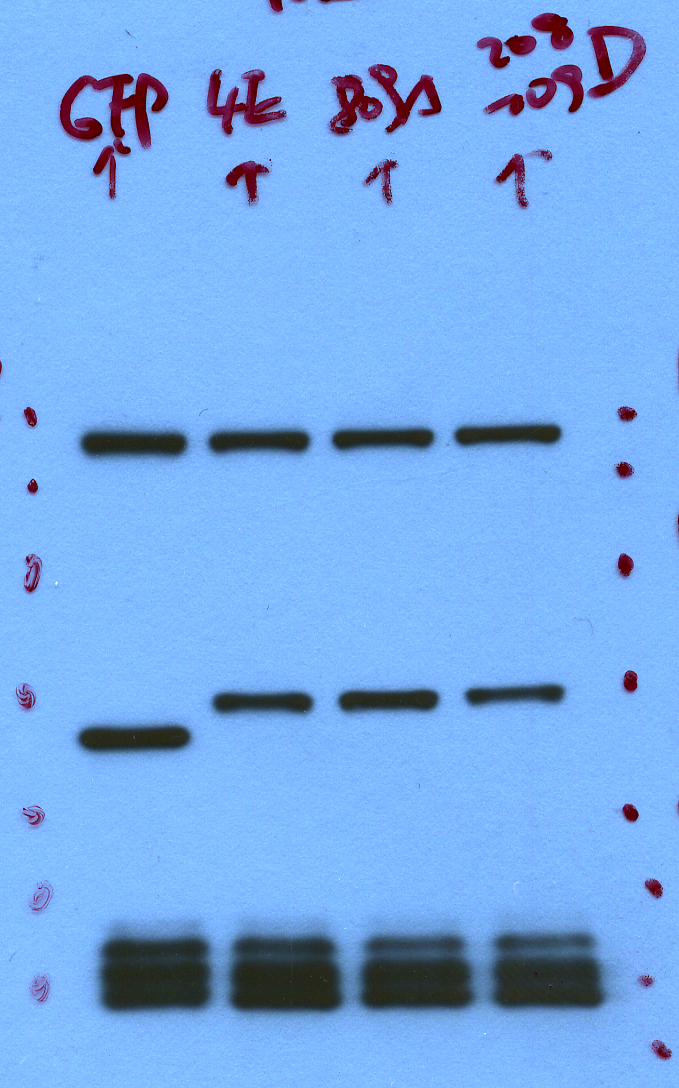

Supplement: Supplementary file 1 [file LSA-2024-02755_SdataF1.zip › LSA-2024-02755_SdataF1.20.tif]

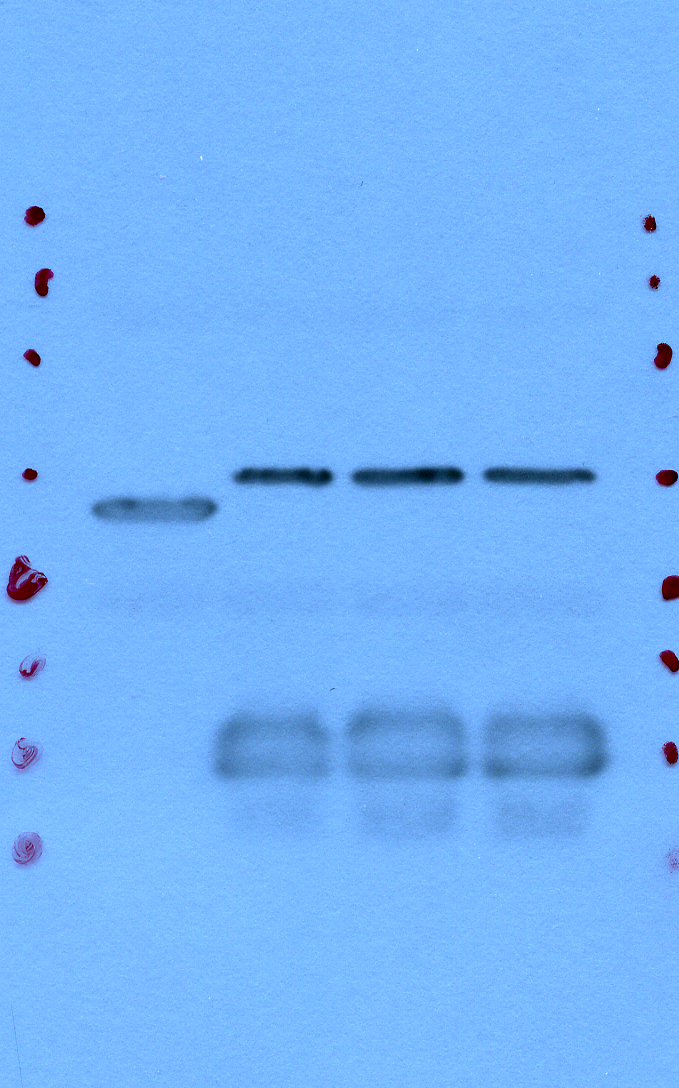

Supplement: Supplementary file 1 [file LSA-2024-02755_SdataF1.zip › LSA-2024-02755_SdataF1.21.tif]

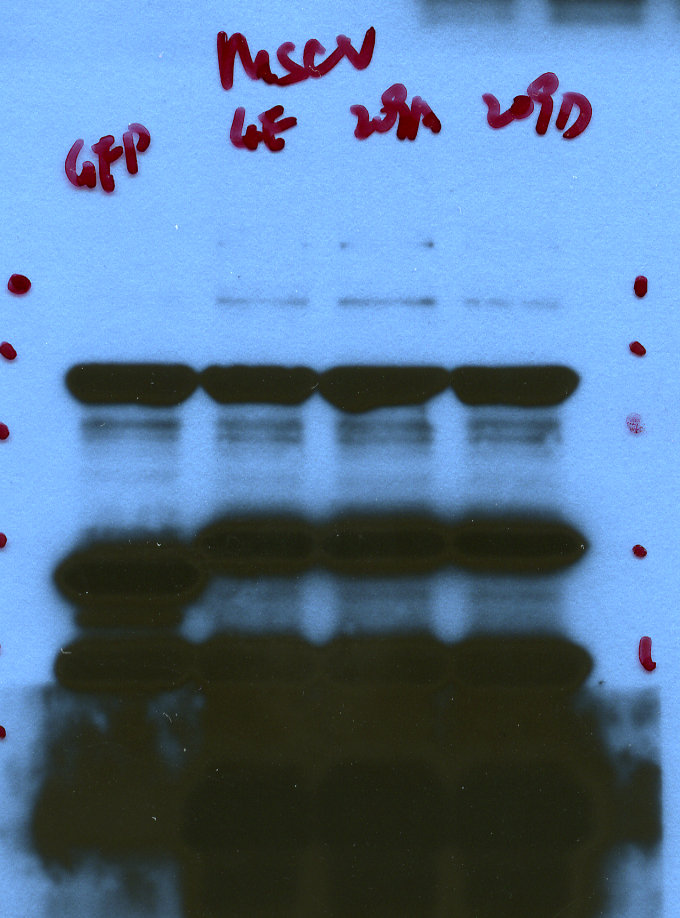

Supplement: Supplementary file 1 [file LSA-2024-02755_SdataF1.zip › LSA-2024-02755_SdataF1.22.tif]

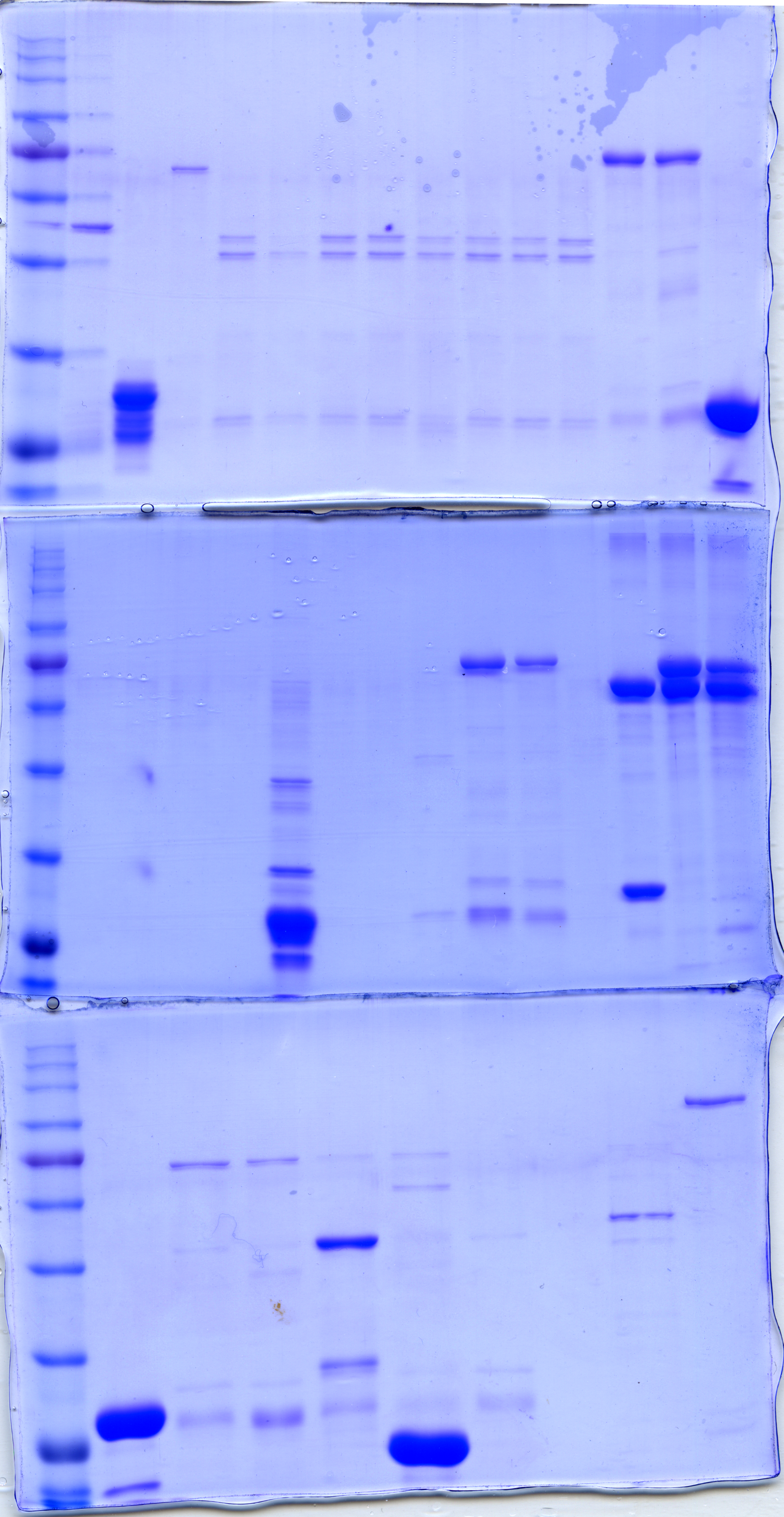

Supplement: Supplementary file 1 [file LSA-2024-02755_SdataF1.zip › LSA-2024-02755_SdataF1.23.tif]

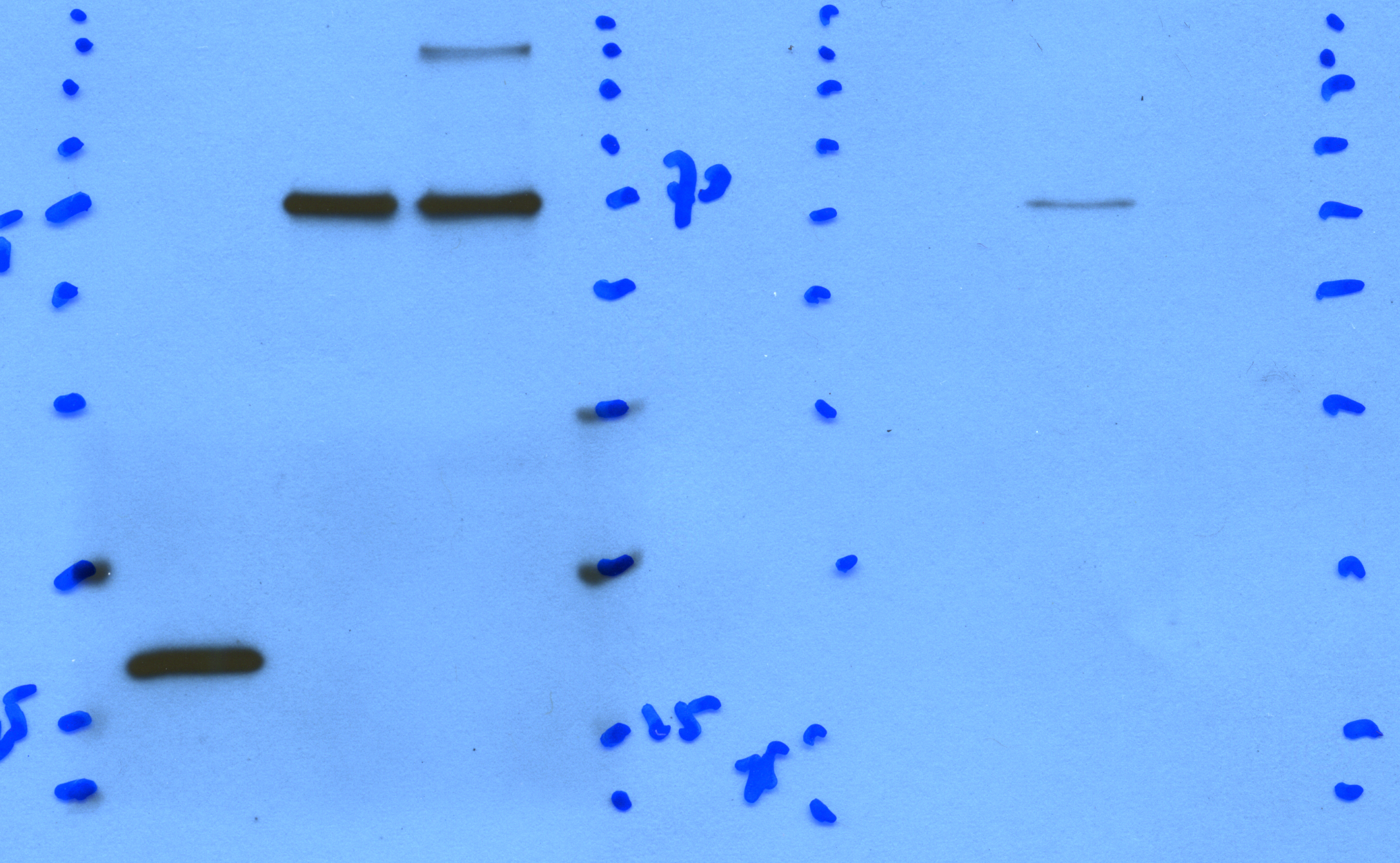

Supplement: Supplementary file 1 [file LSA-2024-02755_SdataF1.zip › LSA-2024-02755_SdataF1.24.tif]

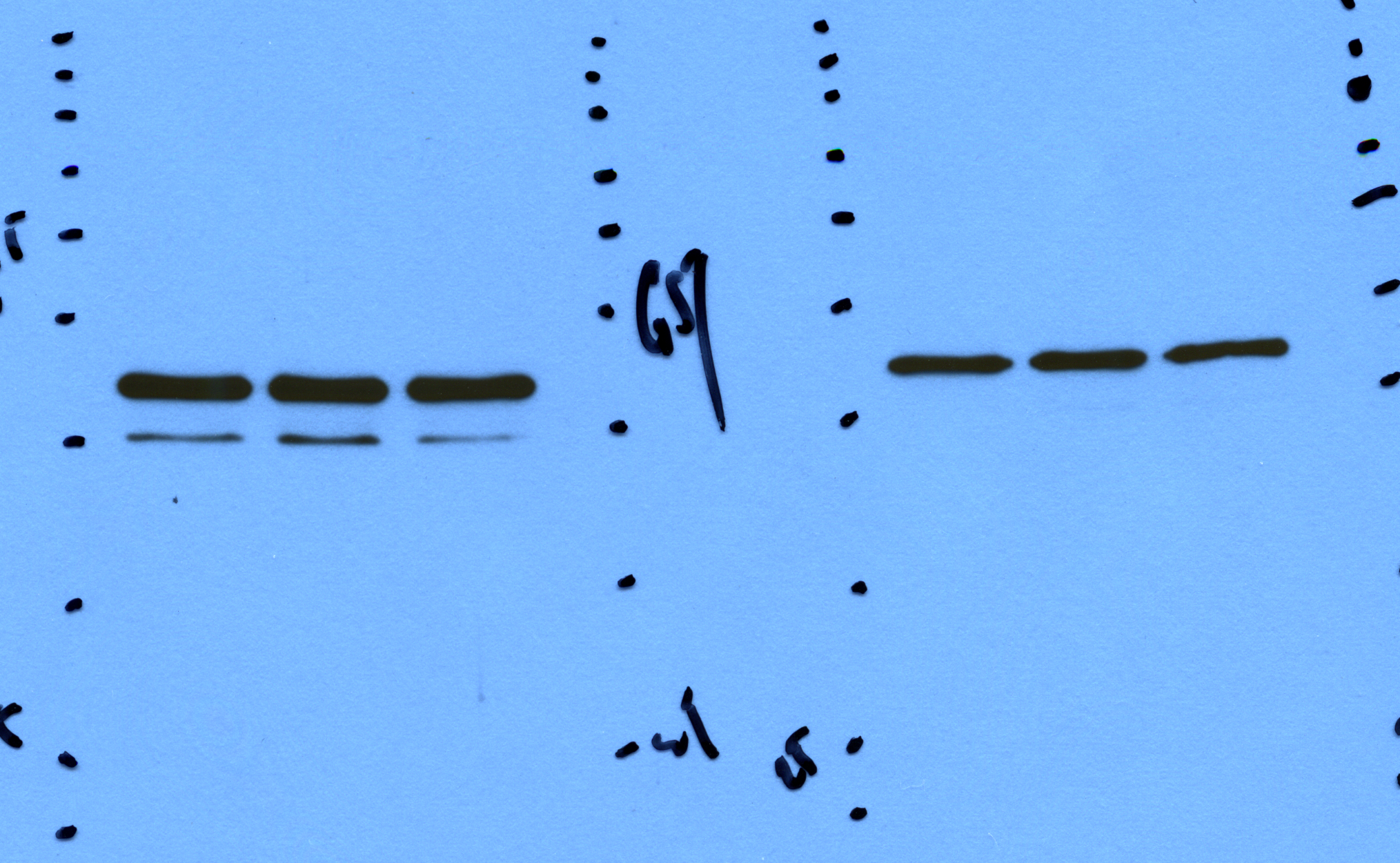

Supplement: Supplementary file 1 [file LSA-2024-02755_SdataF1.zip › LSA-2024-02755_SdataF1.25.tif]

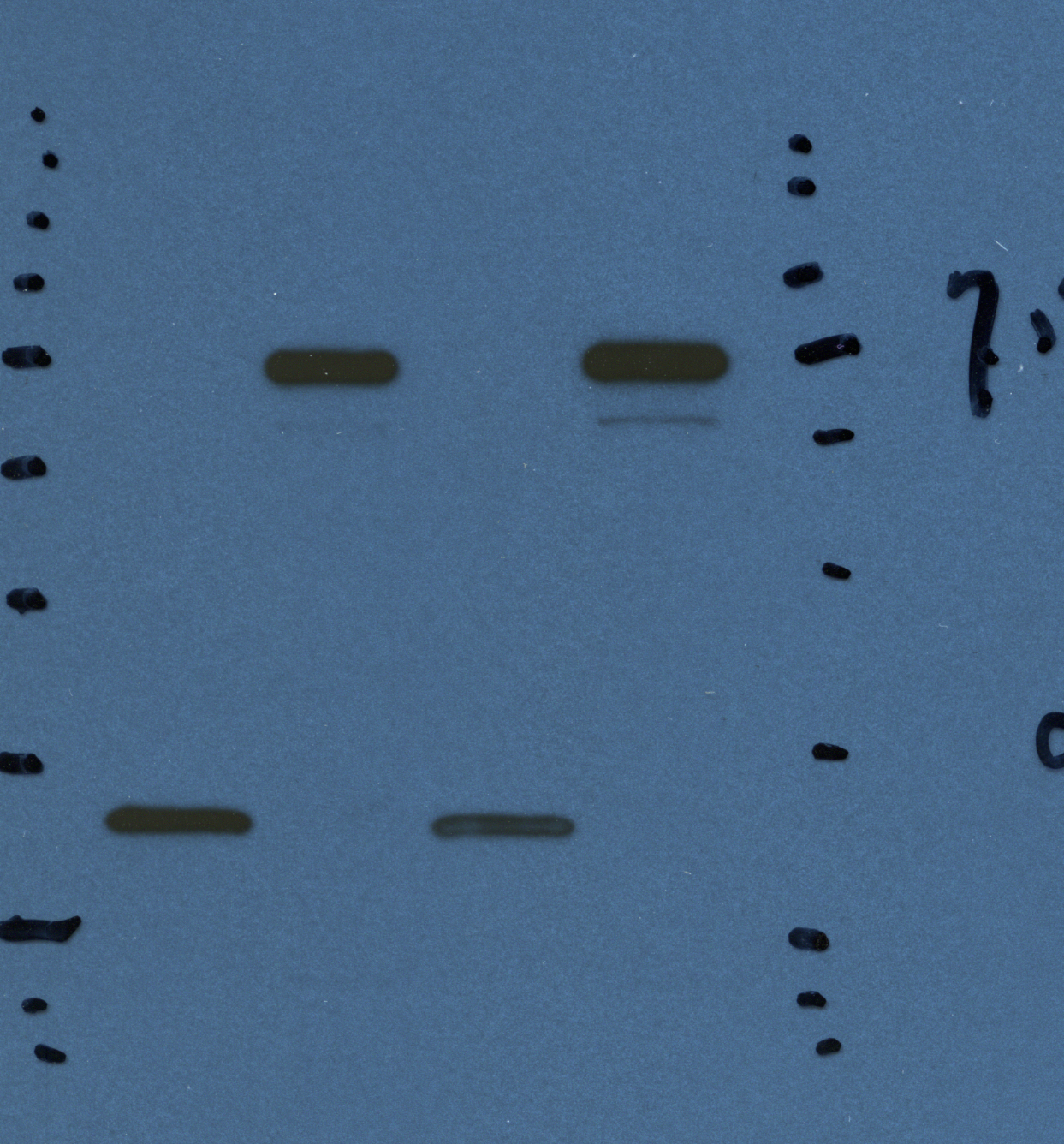

Supplement: Supplementary file 1 [file LSA-2024-02755_SdataF1.zip › LSA-2024-02755_SdataF1.26.tif]

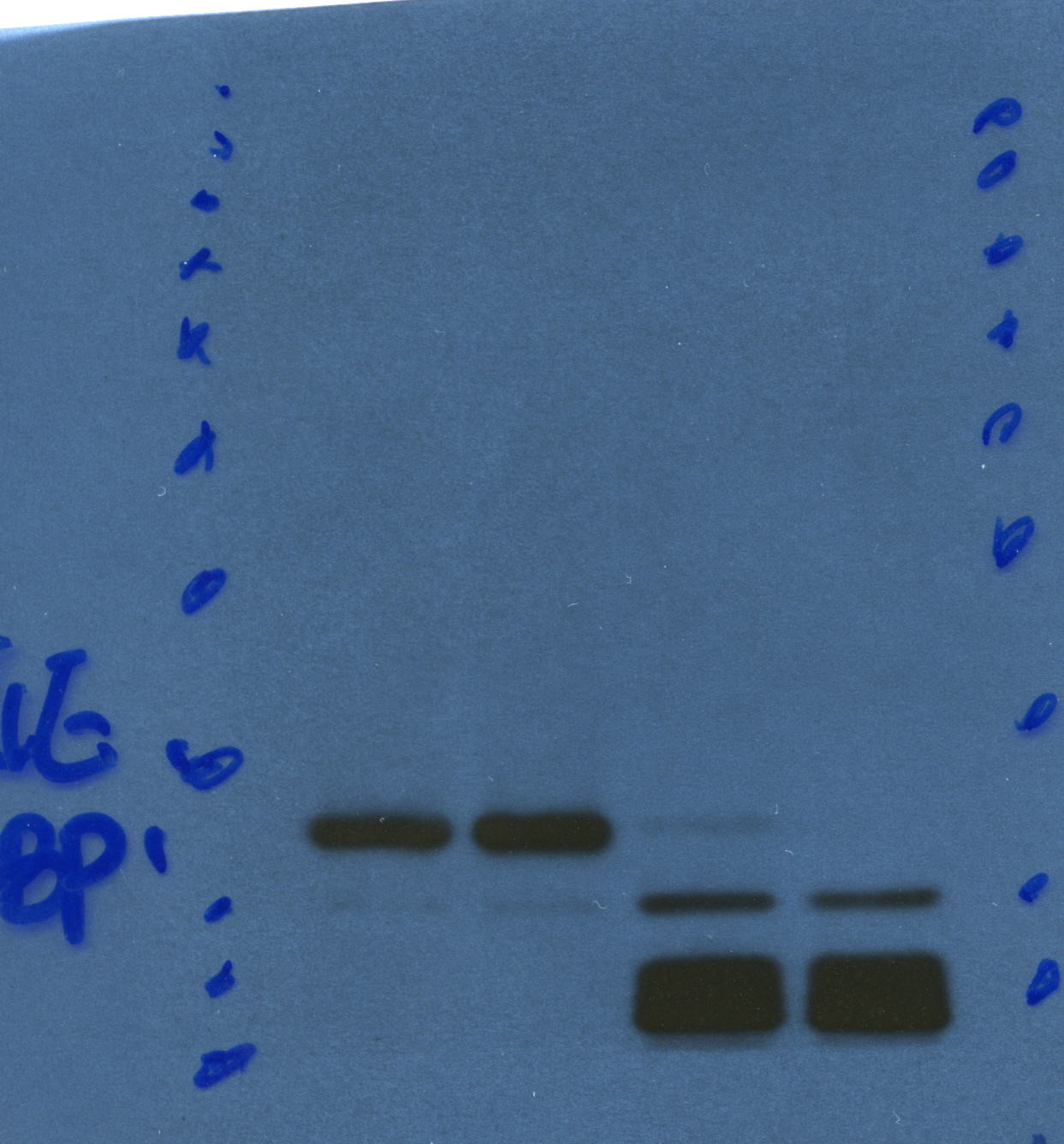

Supplement: Supplementary file 1 [file LSA-2024-02755_SdataF1.zip › LSA-2024-02755_SdataF1.27.tif]

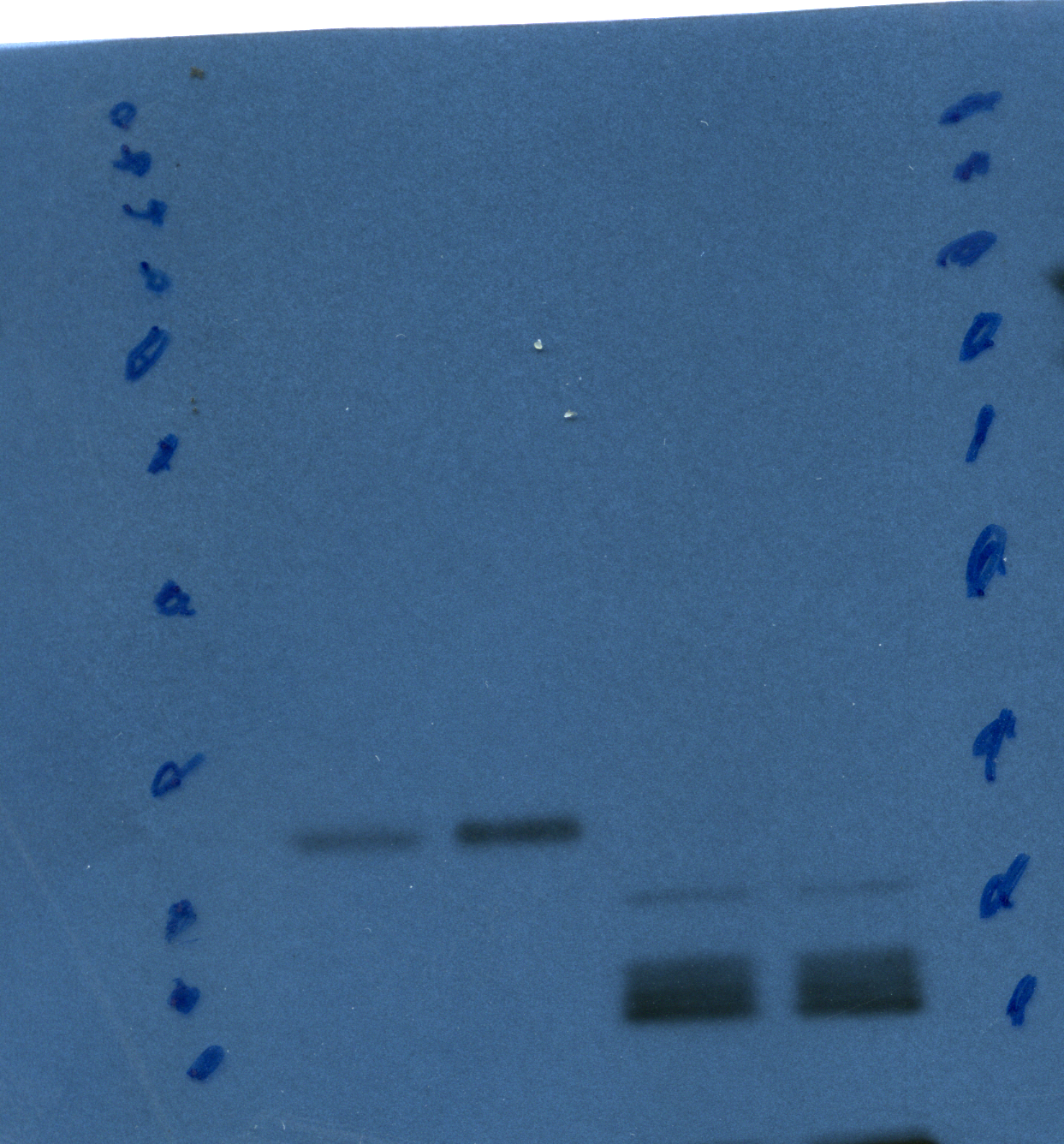

Supplement: Supplementary file 1 [file LSA-2024-02755_SdataF1.zip › LSA-2024-02755_SdataF1.28.tif]

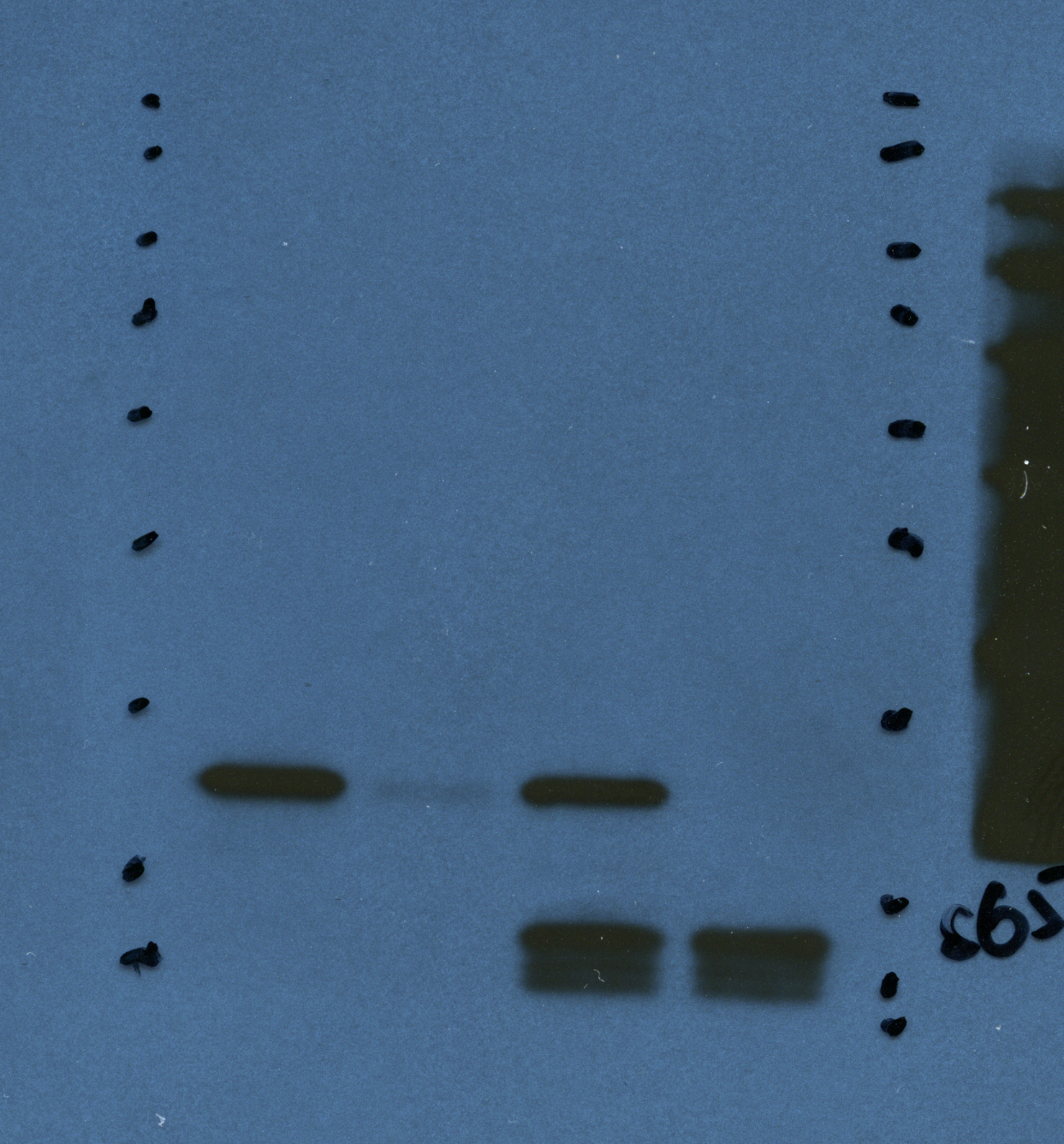

Supplement: Supplementary file 1 [file LSA-2024-02755_SdataF1.zip › LSA-2024-02755_SdataF1.29.tif]

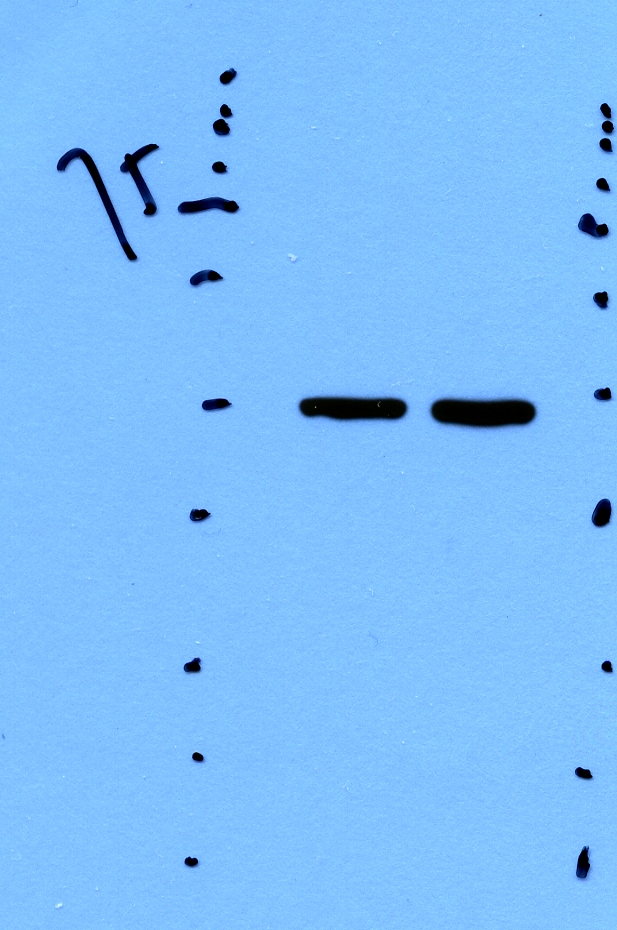

Supplement: Supplementary file 1 [file LSA-2024-02755_SdataF1.zip › LSA-2024-02755_SdataF1.3.tif]

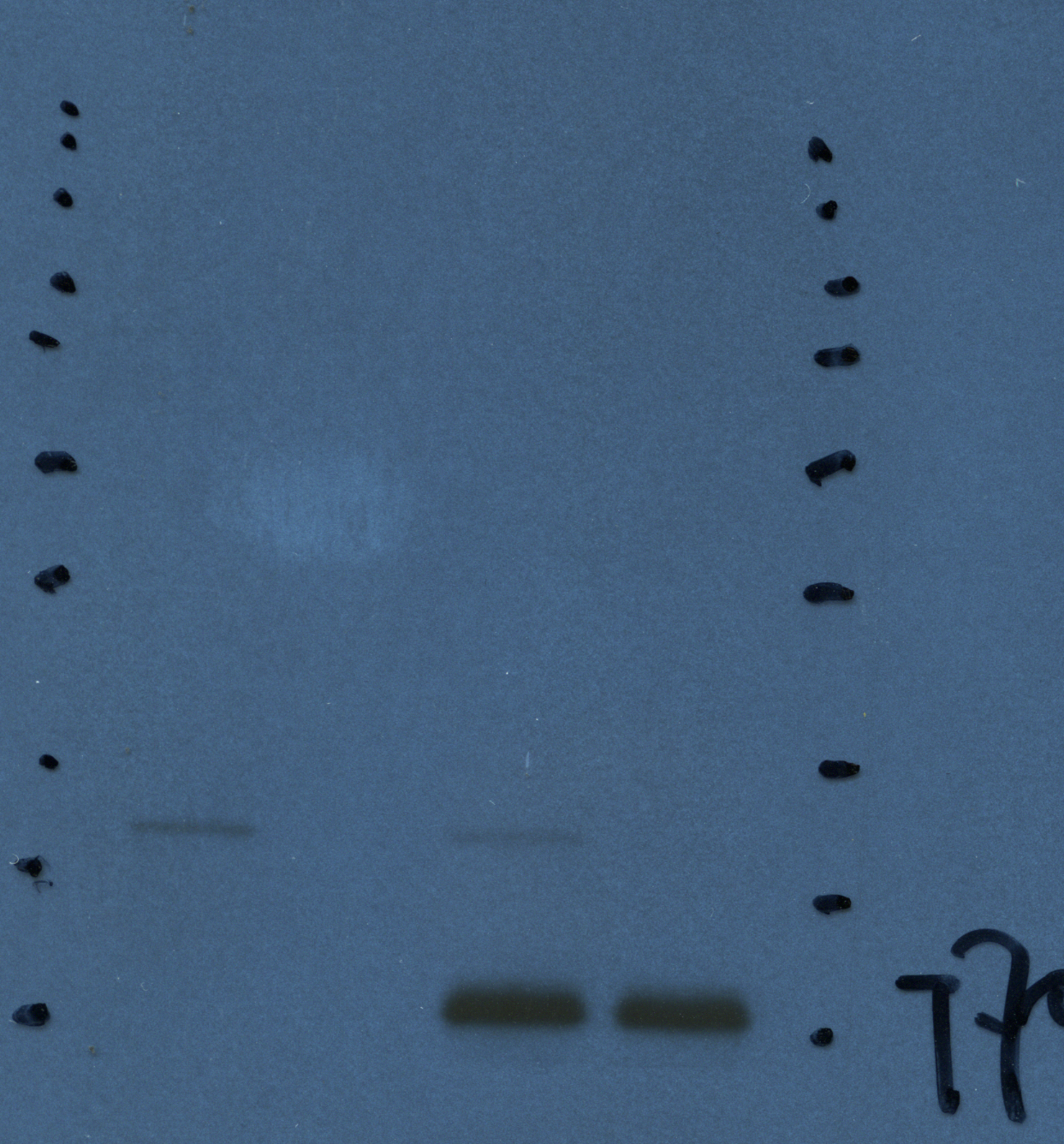

Supplement: Supplementary file 1 [file LSA-2024-02755_SdataF1.zip › LSA-2024-02755_SdataF1.30.tif]

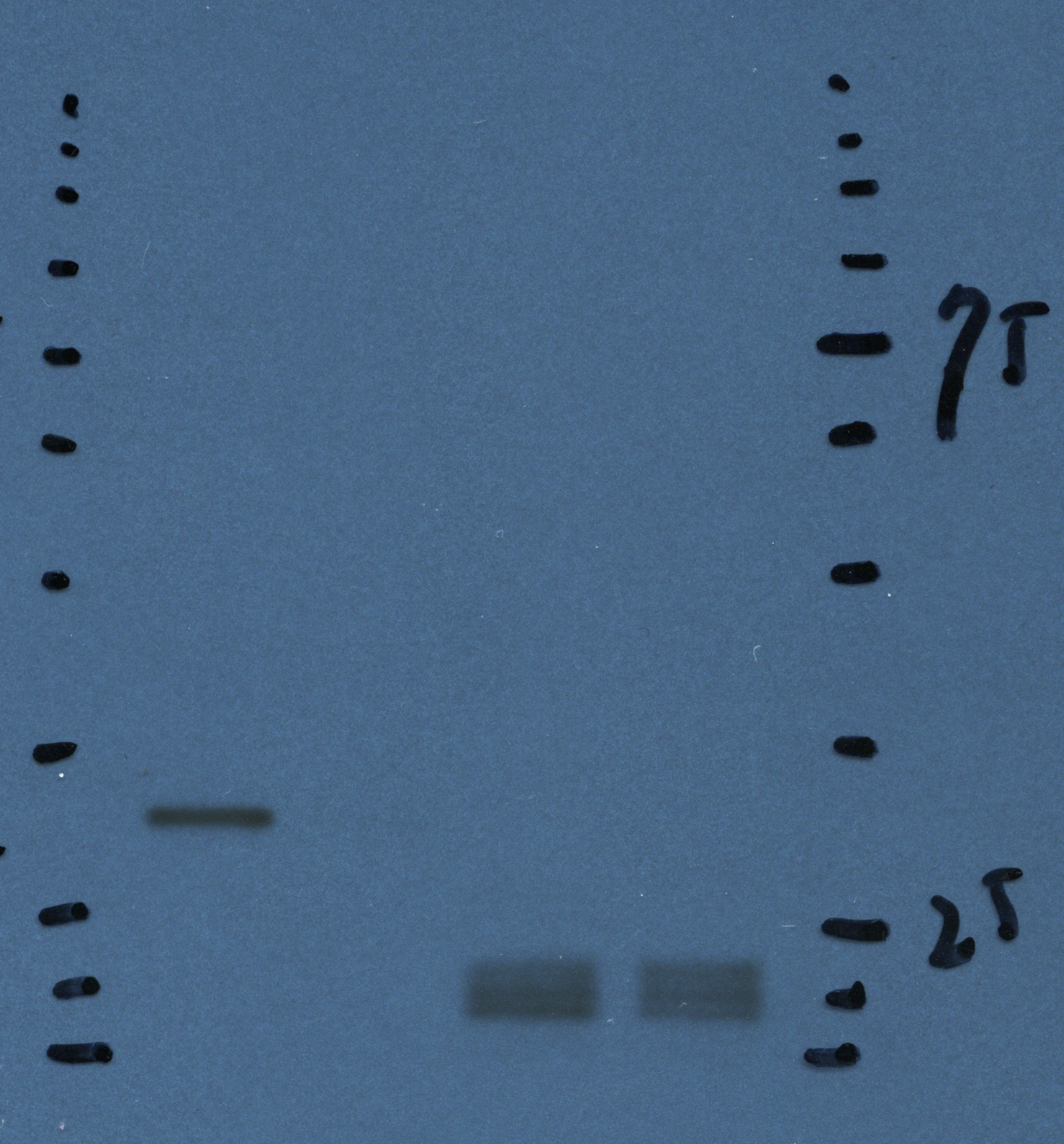

Supplement: Supplementary file 1 [file LSA-2024-02755_SdataF1.zip › LSA-2024-02755_SdataF1.31.tif]

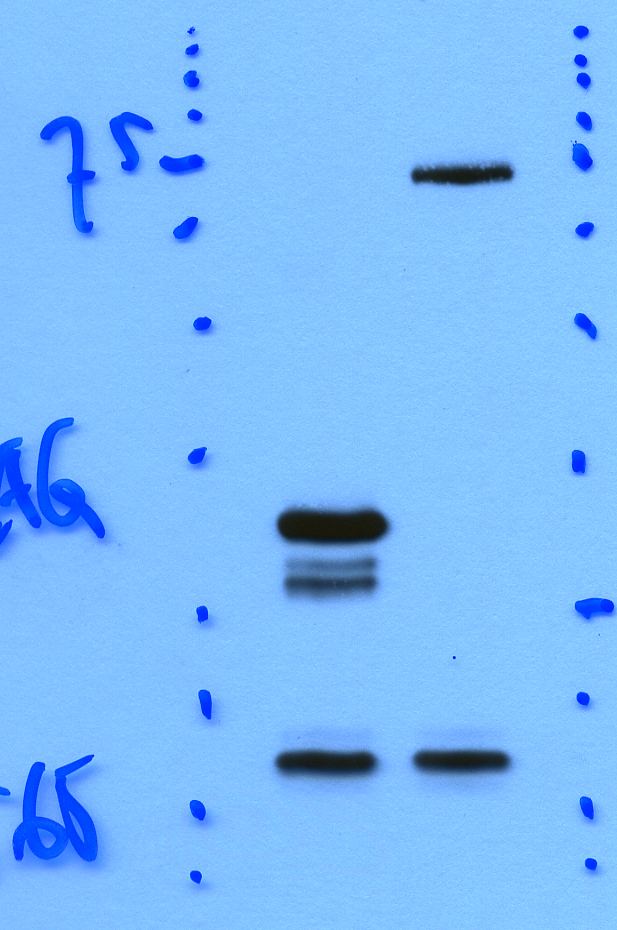

Supplement: Supplementary file 1 [file LSA-2024-02755_SdataF1.zip › LSA-2024-02755_SdataF1.4.tif]

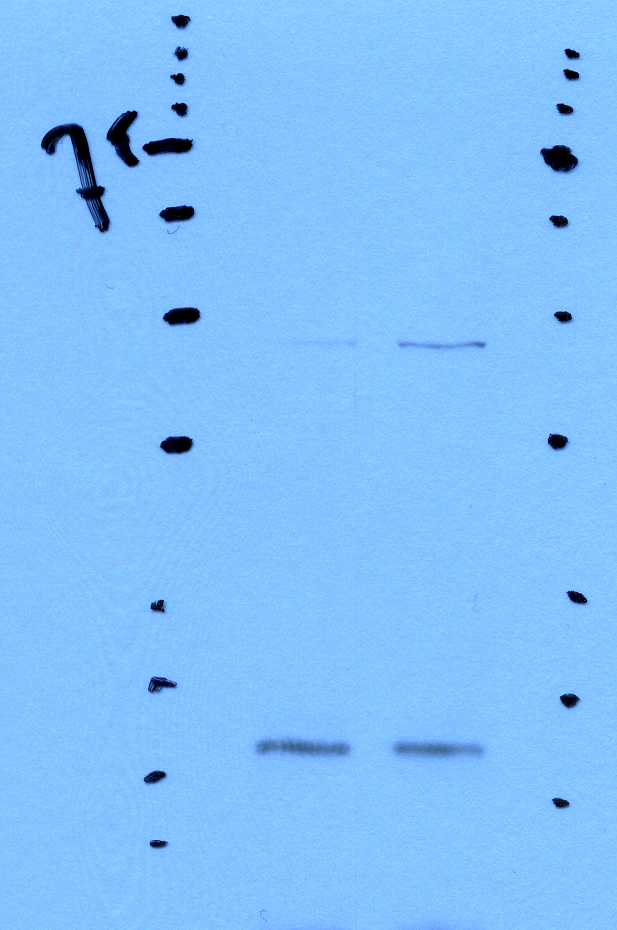

Supplement: Supplementary file 1 [file LSA-2024-02755_SdataF1.zip › LSA-2024-02755_SdataF1.5.tif]

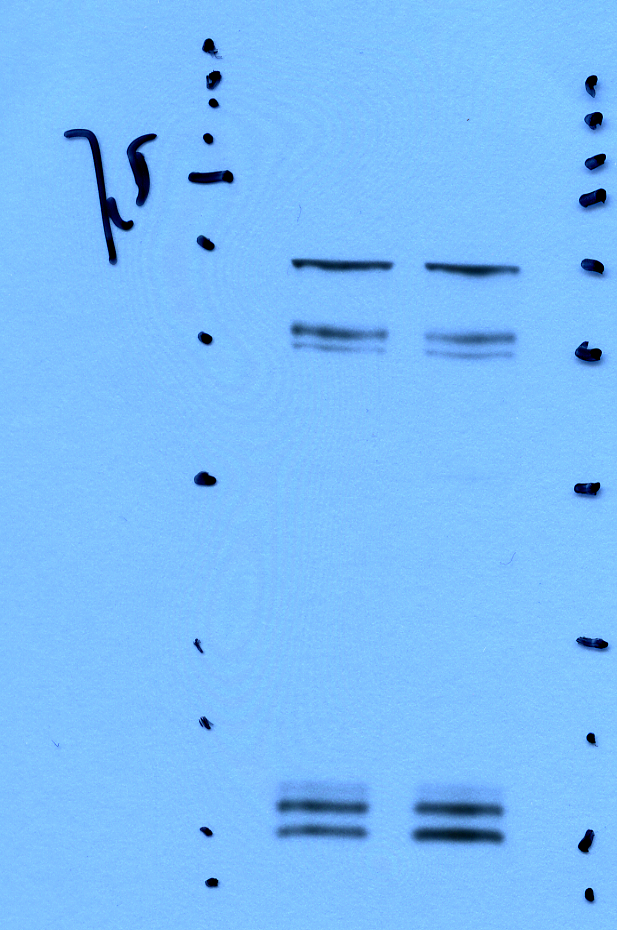

Supplement: Supplementary file 1 [file LSA-2024-02755_SdataF1.zip › LSA-2024-02755_SdataF1.6.tif]

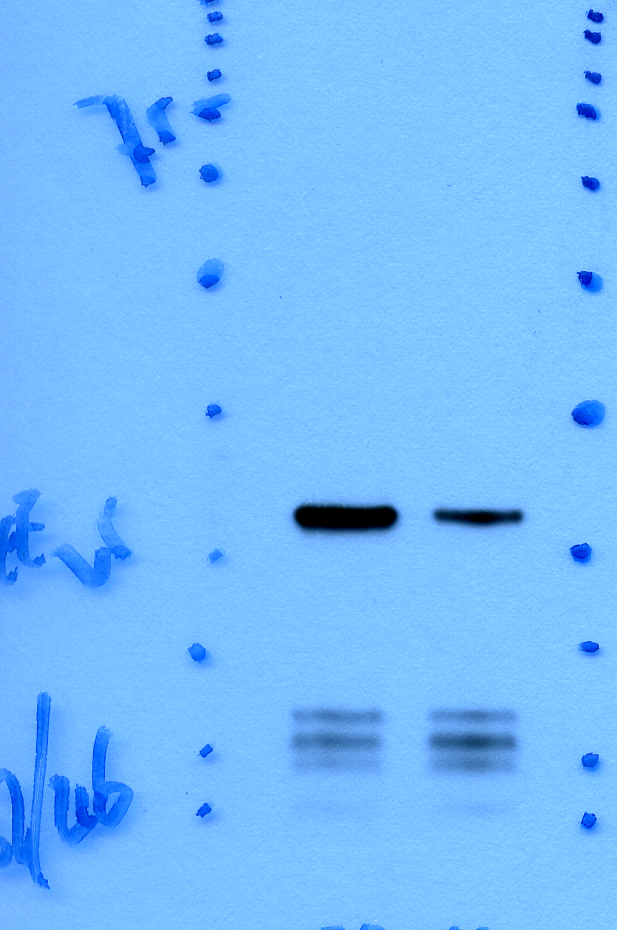

Supplement: Supplementary file 1 [file LSA-2024-02755_SdataF1.zip › LSA-2024-02755_SdataF1.7.tif]

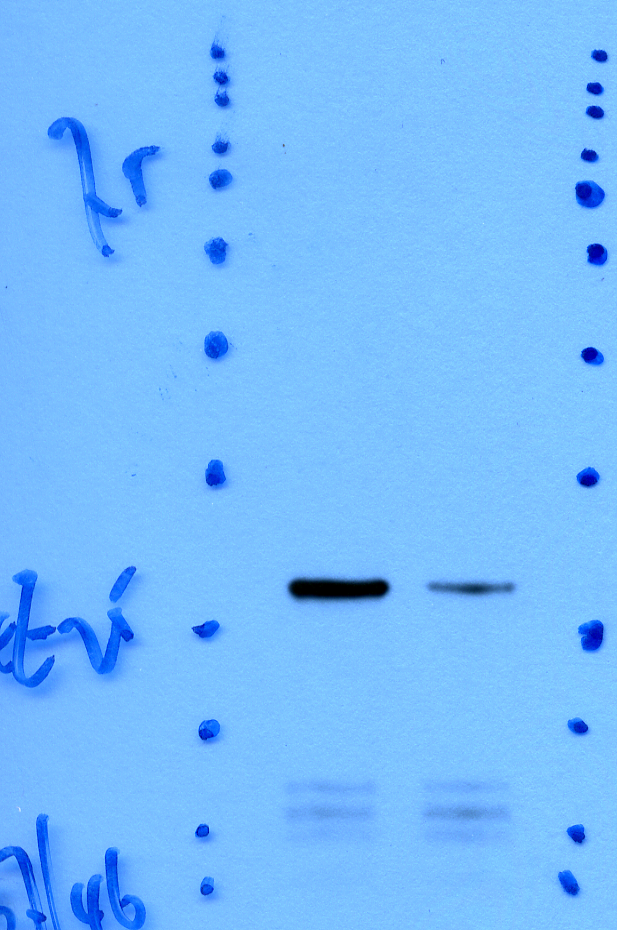

Supplement: Supplementary file 1 [file LSA-2024-02755_SdataF1.zip › LSA-2024-02755_SdataF1.8.tif]

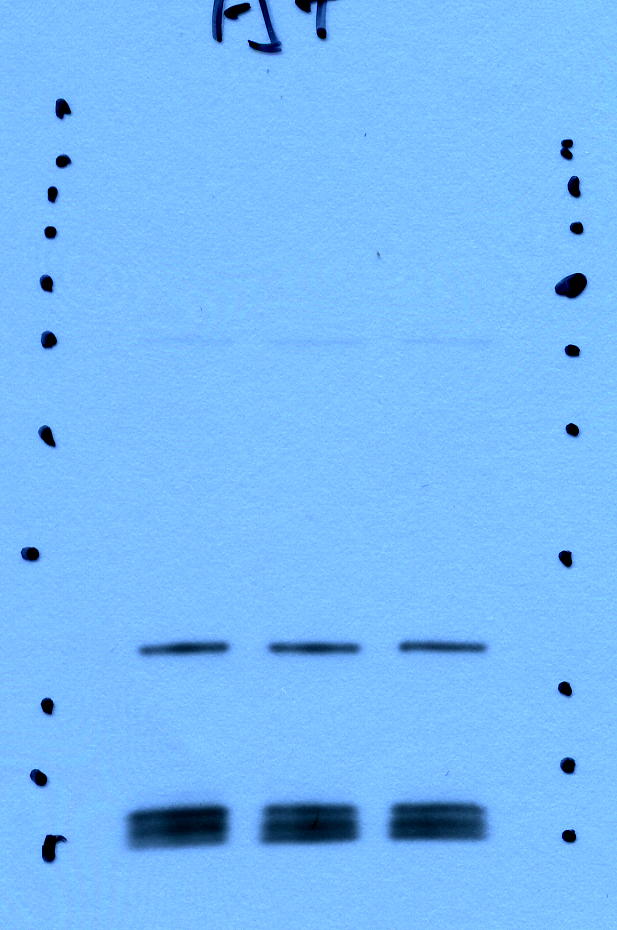

Supplement: Supplementary file 1 [file LSA-2024-02755_SdataF1.zip › LSA-2024-02755_SdataF1.9.tif]

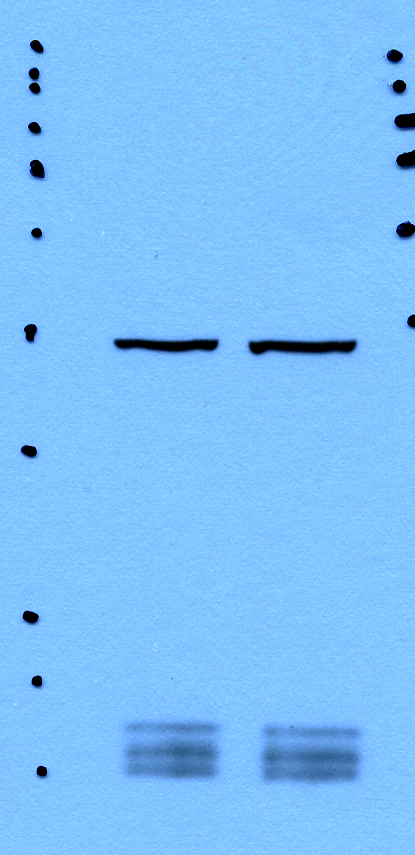

Supplement: Supplementary file 2 [file LSA-2024-02755_SdataFS1.zip › LSA-2024-02755_SdataFS1.1.tif]

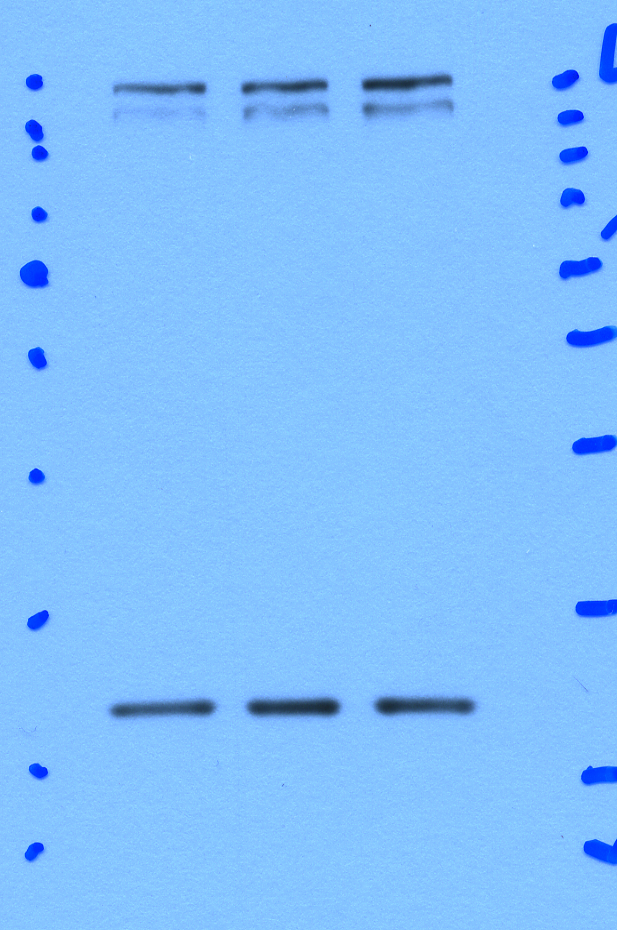

Supplement: Supplementary file 2 [file LSA-2024-02755_SdataFS1.zip › LSA-2024-02755_SdataFS1.10.tif]

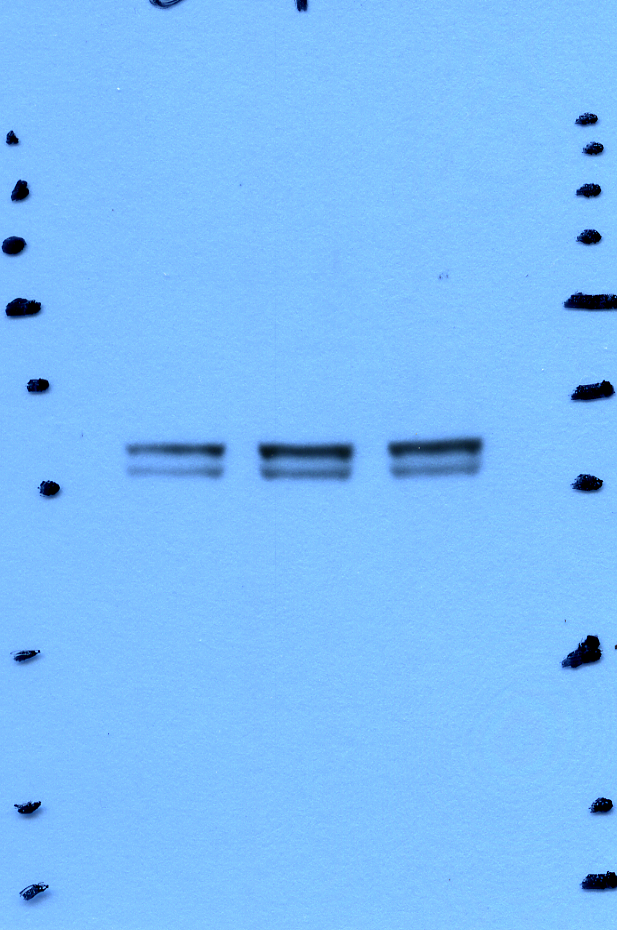

Supplement: Supplementary file 2 [file LSA-2024-02755_SdataFS1.zip › LSA-2024-02755_SdataFS1.11.tif]

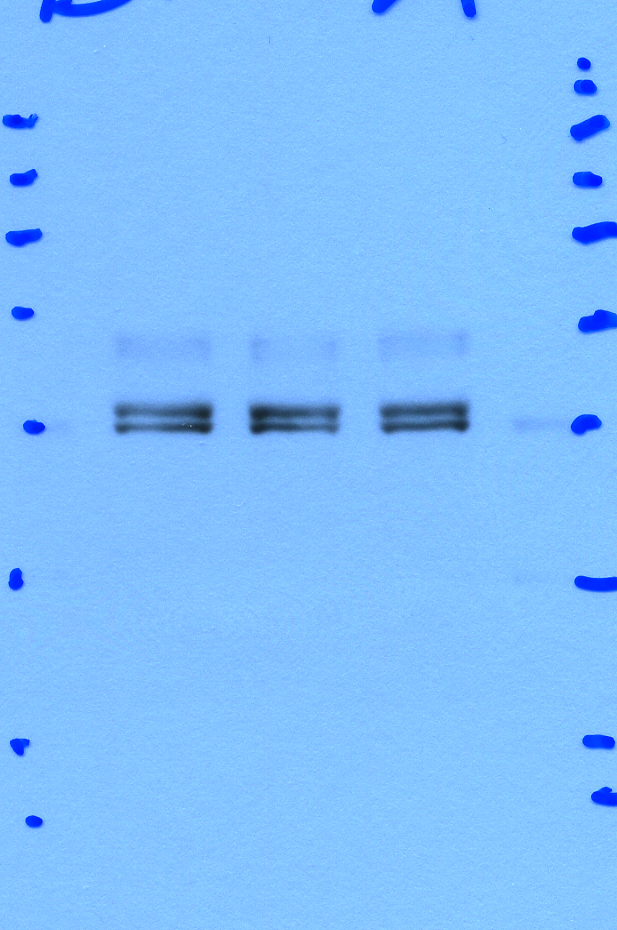

Supplement: Supplementary file 2 [file LSA-2024-02755_SdataFS1.zip › LSA-2024-02755_SdataFS1.12.tif]

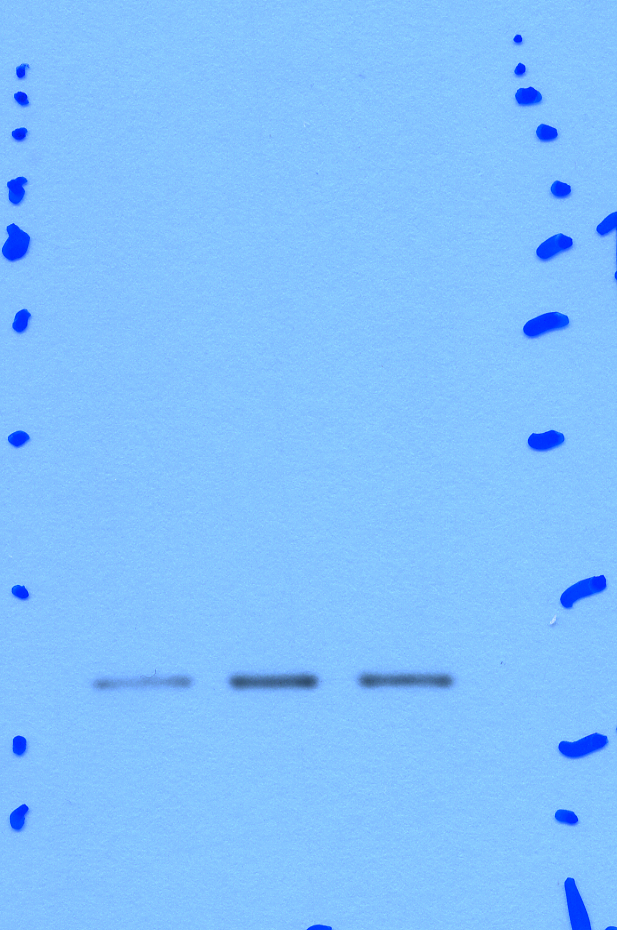

Supplement: Supplementary file 2 [file LSA-2024-02755_SdataFS1.zip › LSA-2024-02755_SdataFS1.13.tif]

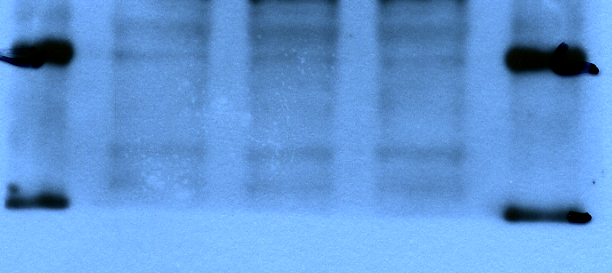

Supplement: Supplementary file 2 [file LSA-2024-02755_SdataFS1.zip › LSA-2024-02755_SdataFS1.14.tif]

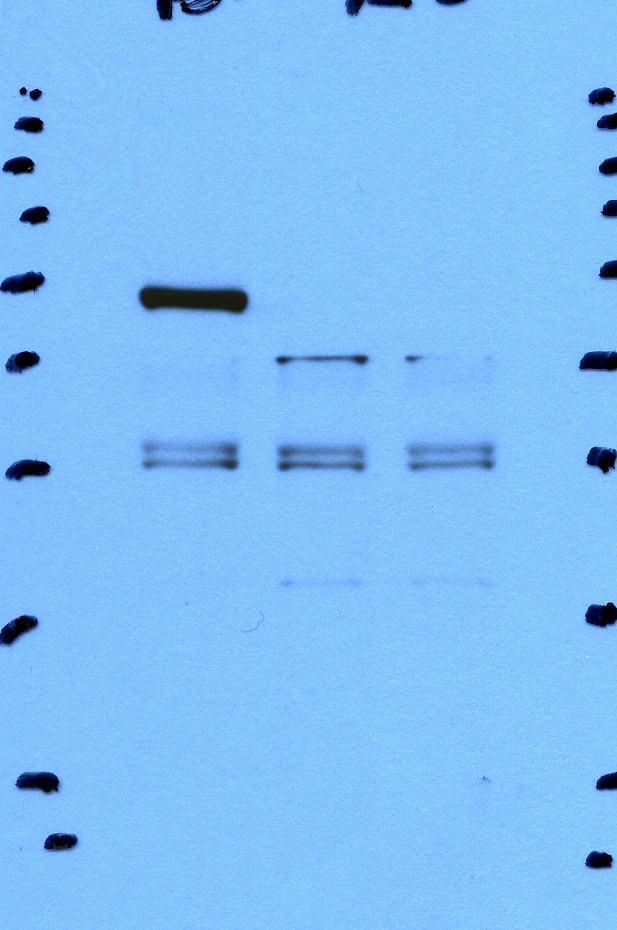

Supplement: Supplementary file 2 [file LSA-2024-02755_SdataFS1.zip › LSA-2024-02755_SdataFS1.15.tif]

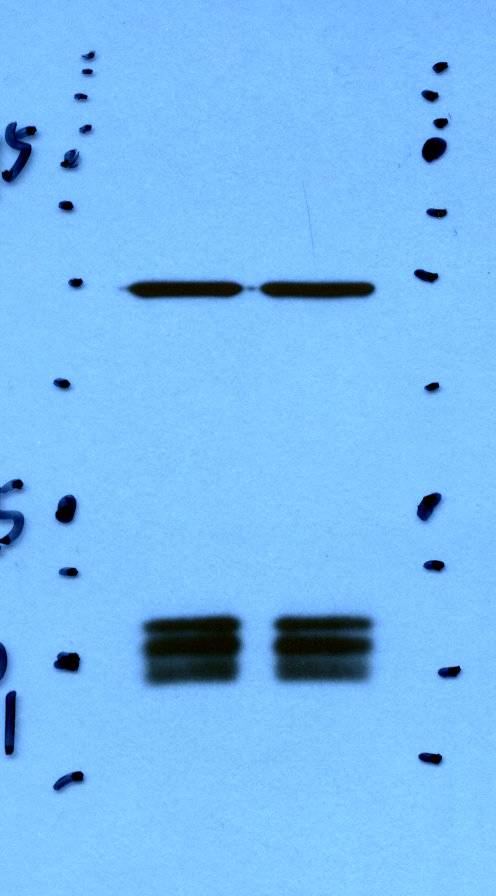

Supplement: Supplementary file 2 [file LSA-2024-02755_SdataFS1.zip › LSA-2024-02755_SdataFS1.16.tif]

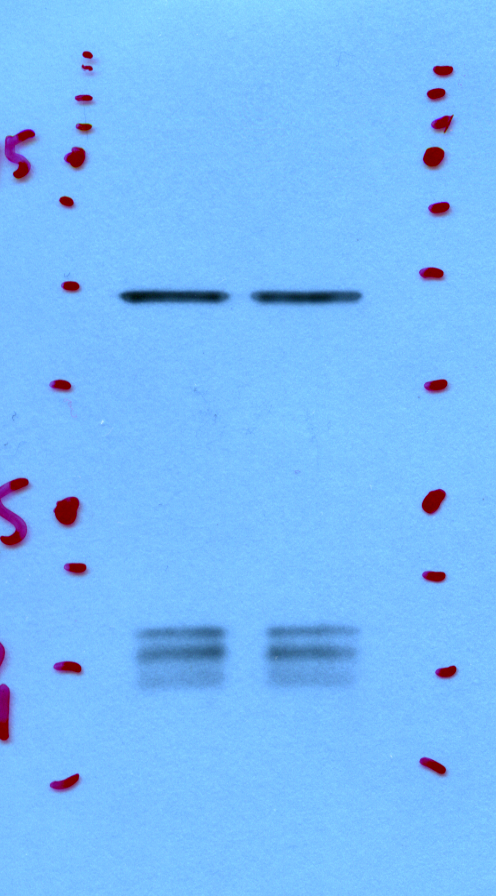

Supplement: Supplementary file 2 [file LSA-2024-02755_SdataFS1.zip › LSA-2024-02755_SdataFS1.17.tif]

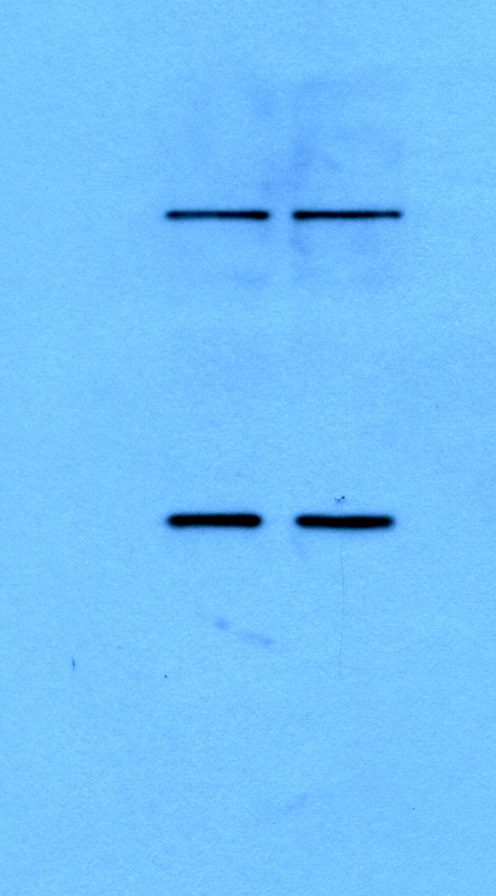

Supplement: Supplementary file 2 [file LSA-2024-02755_SdataFS1.zip › LSA-2024-02755_SdataFS1.18.tif]

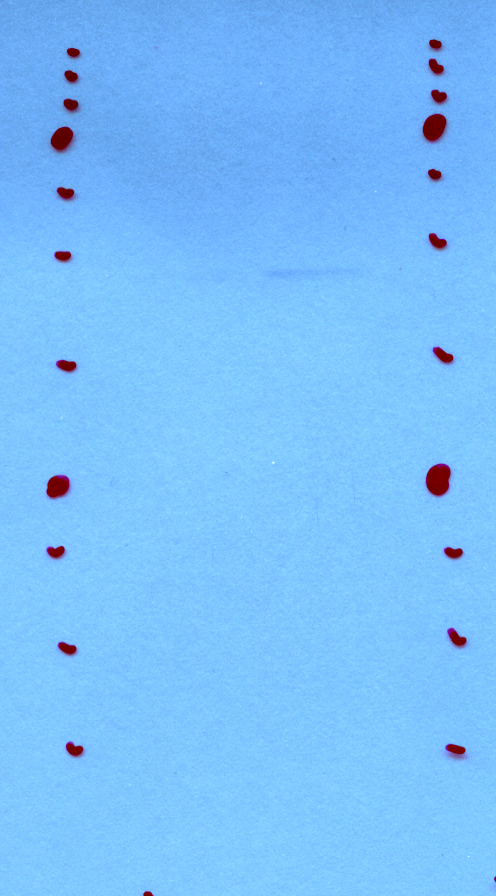

Supplement: Supplementary file 2 [file LSA-2024-02755_SdataFS1.zip › LSA-2024-02755_SdataFS1.19.tif]

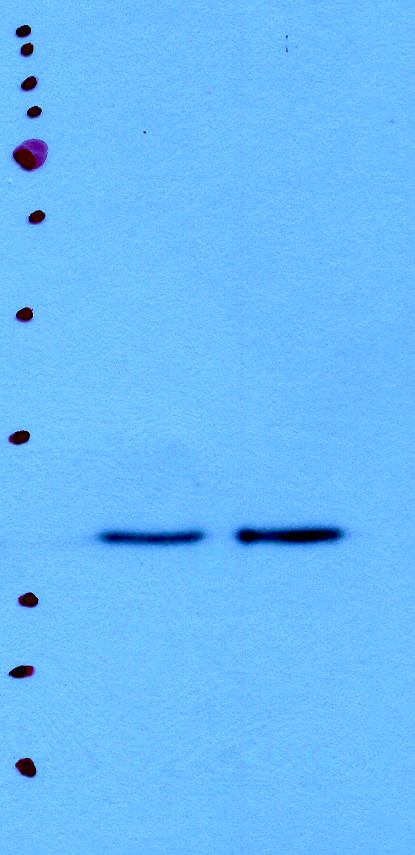

Supplement: Supplementary file 2 [file LSA-2024-02755_SdataFS1.zip › LSA-2024-02755_SdataFS1.2.tif]

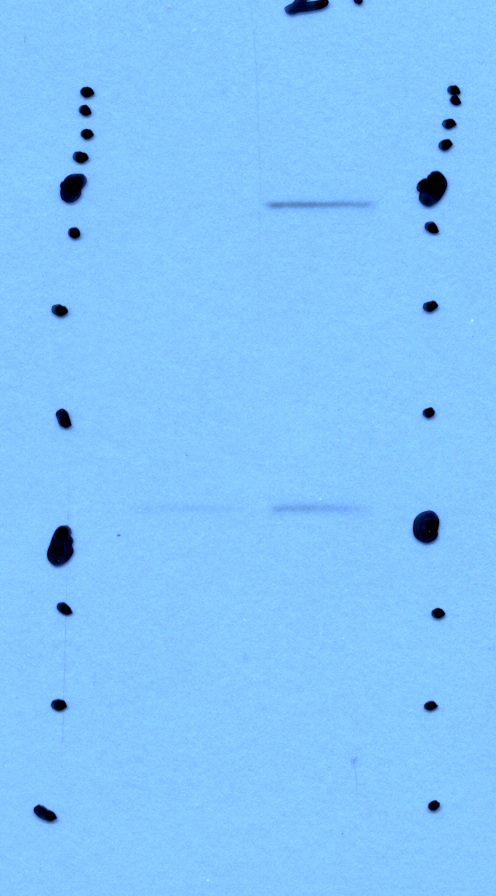

Supplement: Supplementary file 2 [file LSA-2024-02755_SdataFS1.zip › LSA-2024-02755_SdataFS1.20.tif]

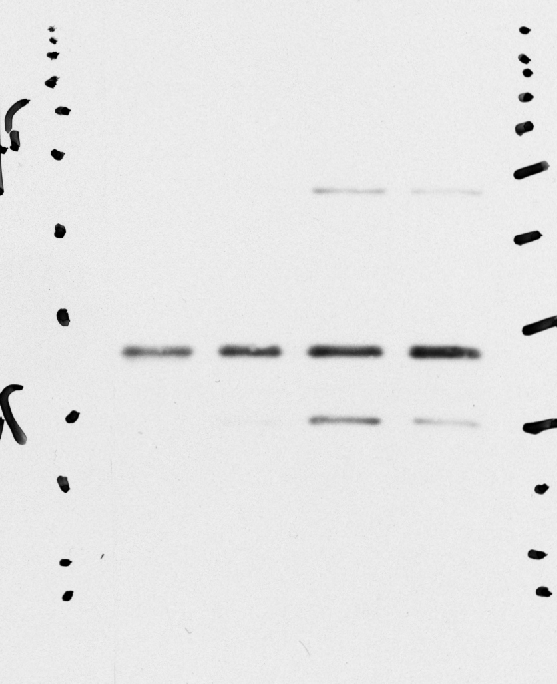

Supplement: Supplementary file 2 [file LSA-2024-02755_SdataFS1.zip › LSA-2024-02755_SdataFS1.21.tif]

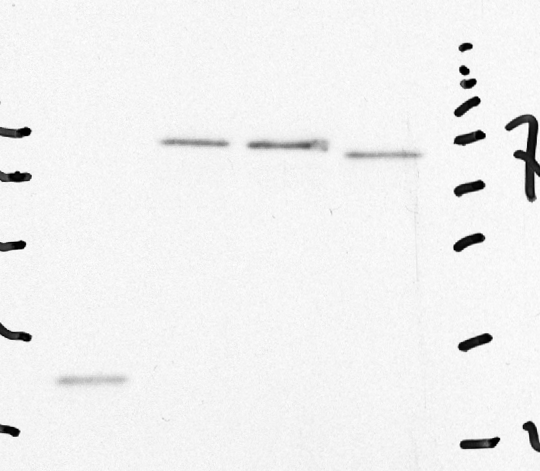

Supplement: Supplementary file 2 [file LSA-2024-02755_SdataFS1.zip › LSA-2024-02755_SdataFS1.22.tif]

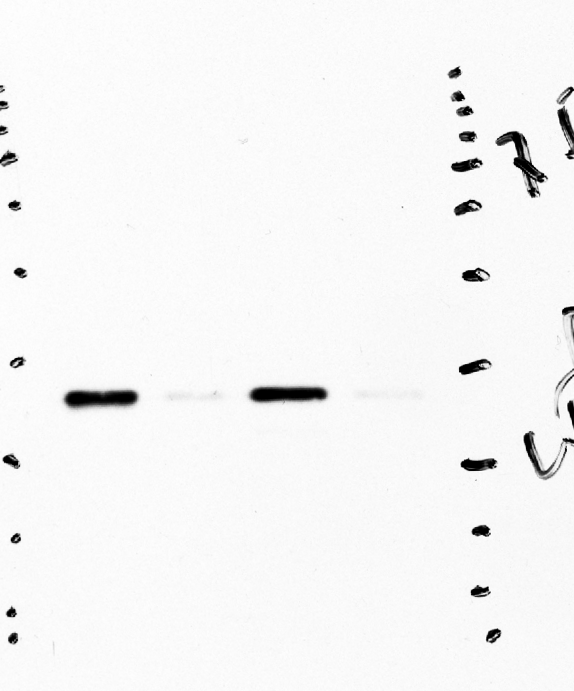

Supplement: Supplementary file 2 [file LSA-2024-02755_SdataFS1.zip › LSA-2024-02755_SdataFS1.23.tif]

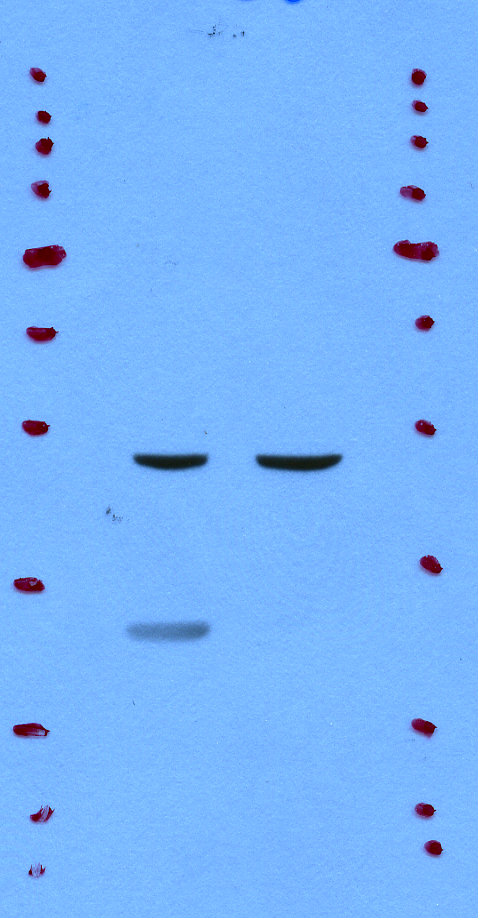

Supplement: Supplementary file 2 [file LSA-2024-02755_SdataFS1.zip › LSA-2024-02755_SdataFS1.24.tif]

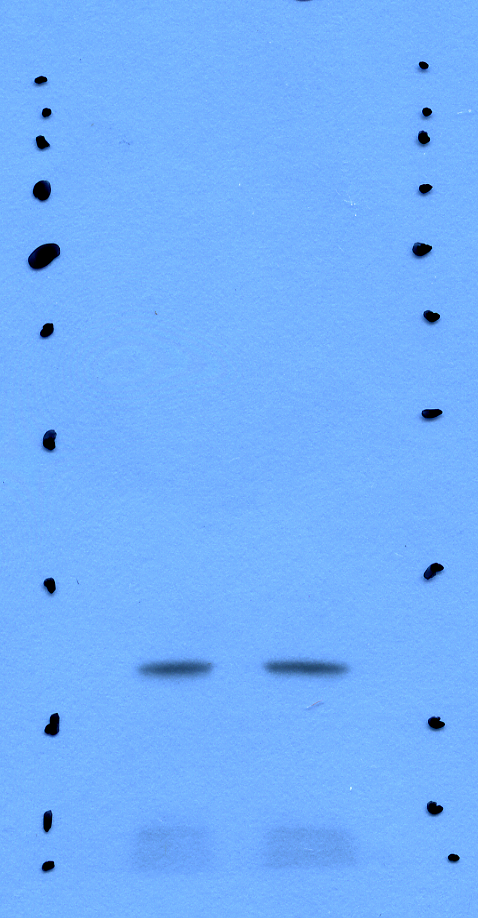

Supplement: Supplementary file 2 [file LSA-2024-02755_SdataFS1.zip › LSA-2024-02755_SdataFS1.25.tif]

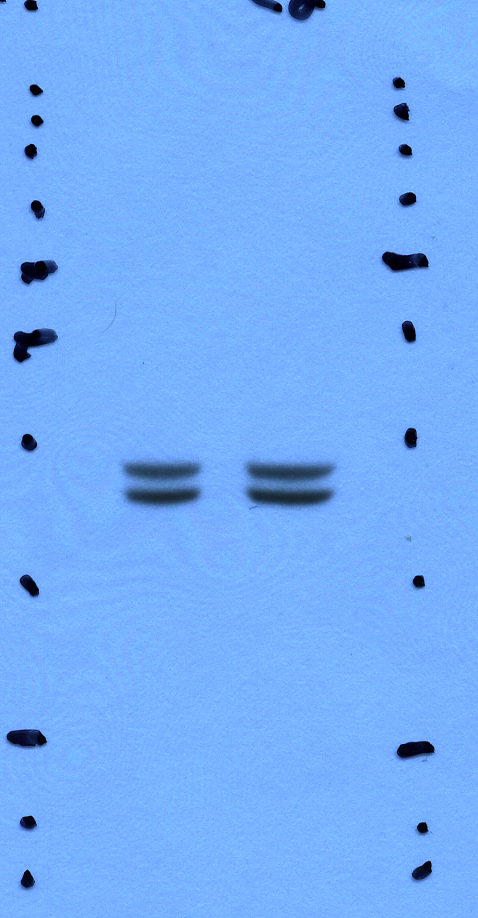

Supplement: Supplementary file 2 [file LSA-2024-02755_SdataFS1.zip › LSA-2024-02755_SdataFS1.26.tif]

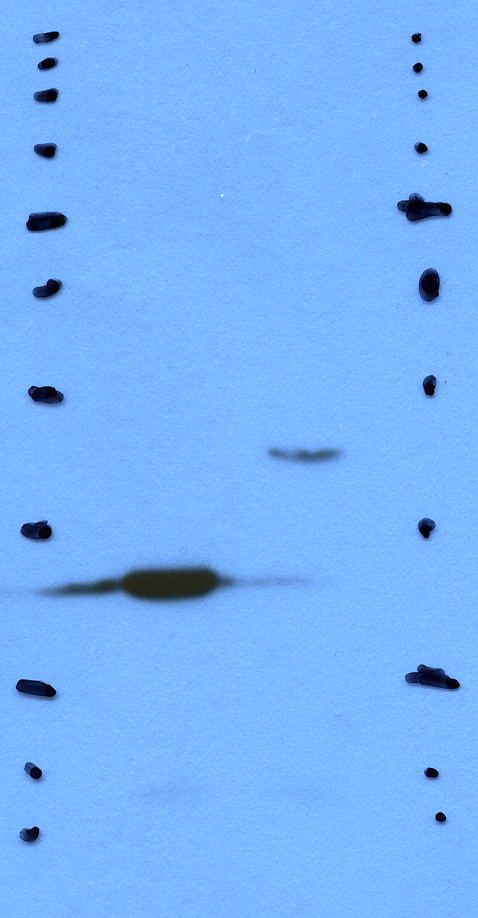

Supplement: Supplementary file 2 [file LSA-2024-02755_SdataFS1.zip › LSA-2024-02755_SdataFS1.27.tif]

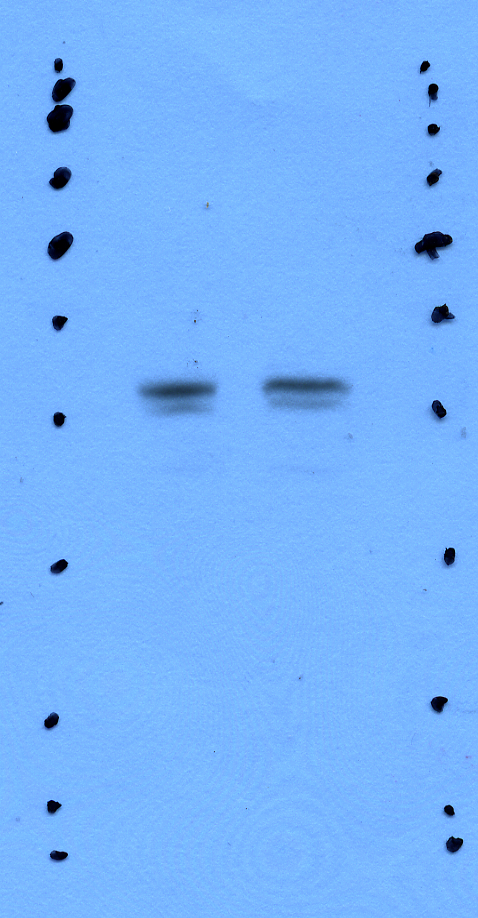

Supplement: Supplementary file 2 [file LSA-2024-02755_SdataFS1.zip › LSA-2024-02755_SdataFS1.28.tif]

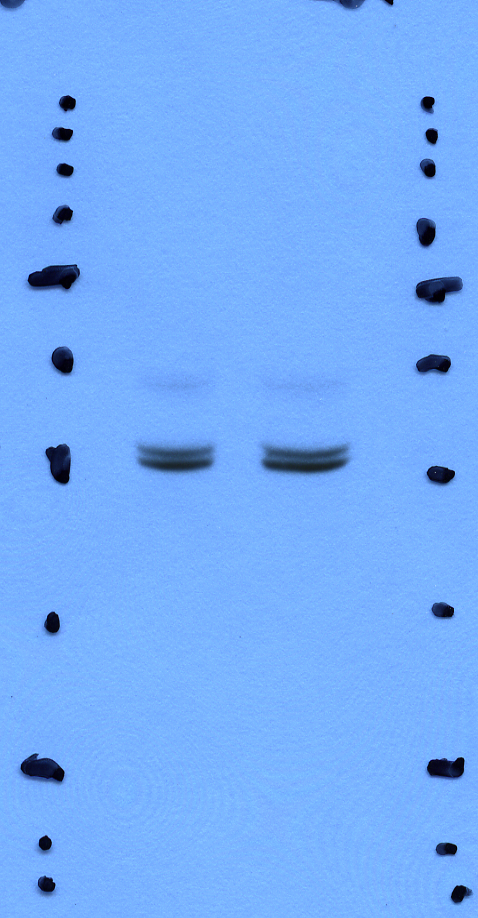

Supplement: Supplementary file 2 [file LSA-2024-02755_SdataFS1.zip › LSA-2024-02755_SdataFS1.29.tif]

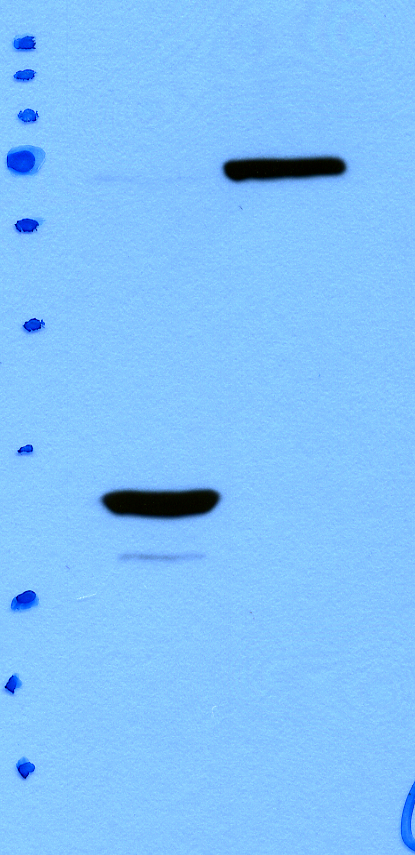

Supplement: Supplementary file 2 [file LSA-2024-02755_SdataFS1.zip › LSA-2024-02755_SdataFS1.3.tif]

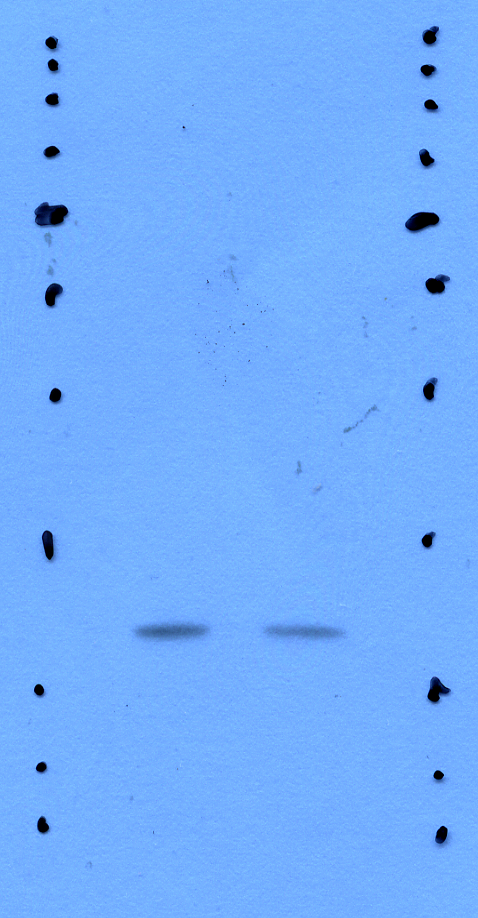

Supplement: Supplementary file 2 [file LSA-2024-02755_SdataFS1.zip › LSA-2024-02755_SdataFS1.30.tif]

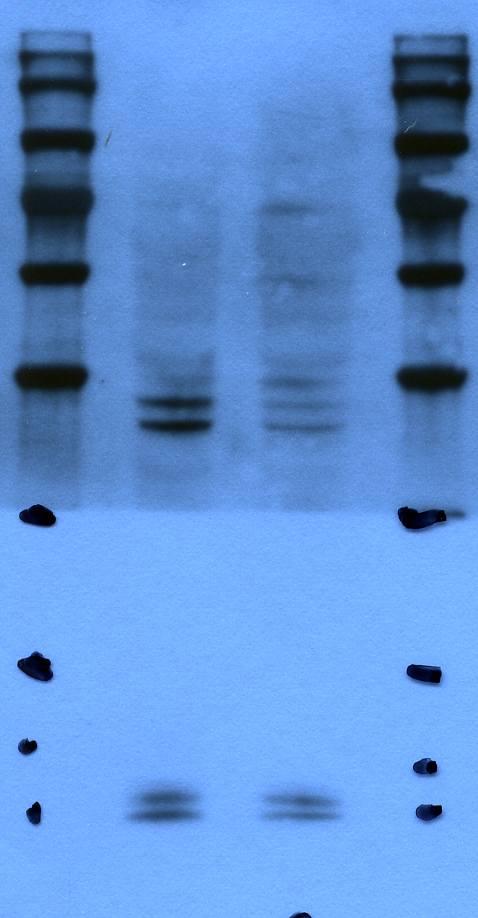

Supplement: Supplementary file 2 [file LSA-2024-02755_SdataFS1.zip › LSA-2024-02755_SdataFS1.31.tif]

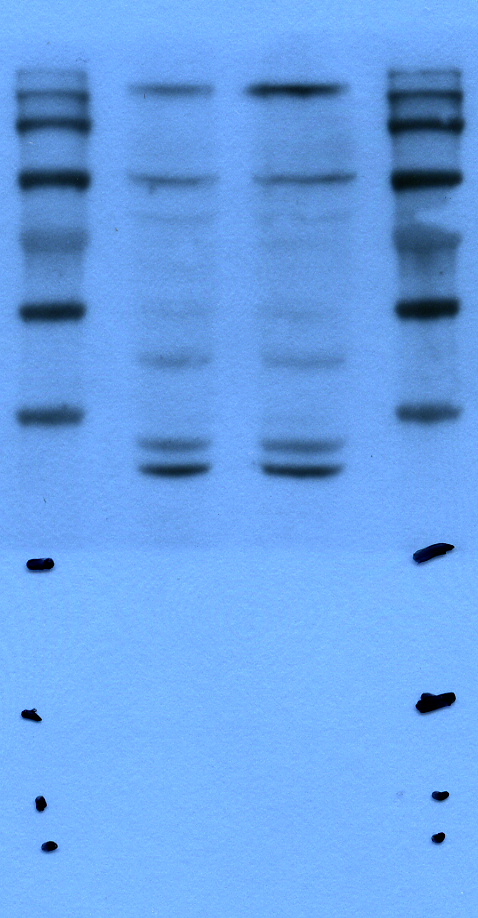

Supplement: Supplementary file 2 [file LSA-2024-02755_SdataFS1.zip › LSA-2024-02755_SdataFS1.32.tif]

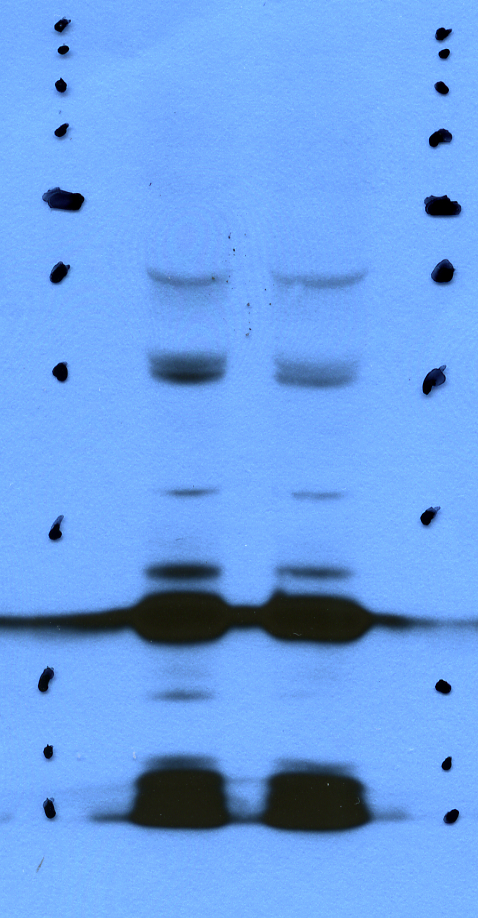

Supplement: Supplementary file 2 [file LSA-2024-02755_SdataFS1.zip › LSA-2024-02755_SdataFS1.33.tif]

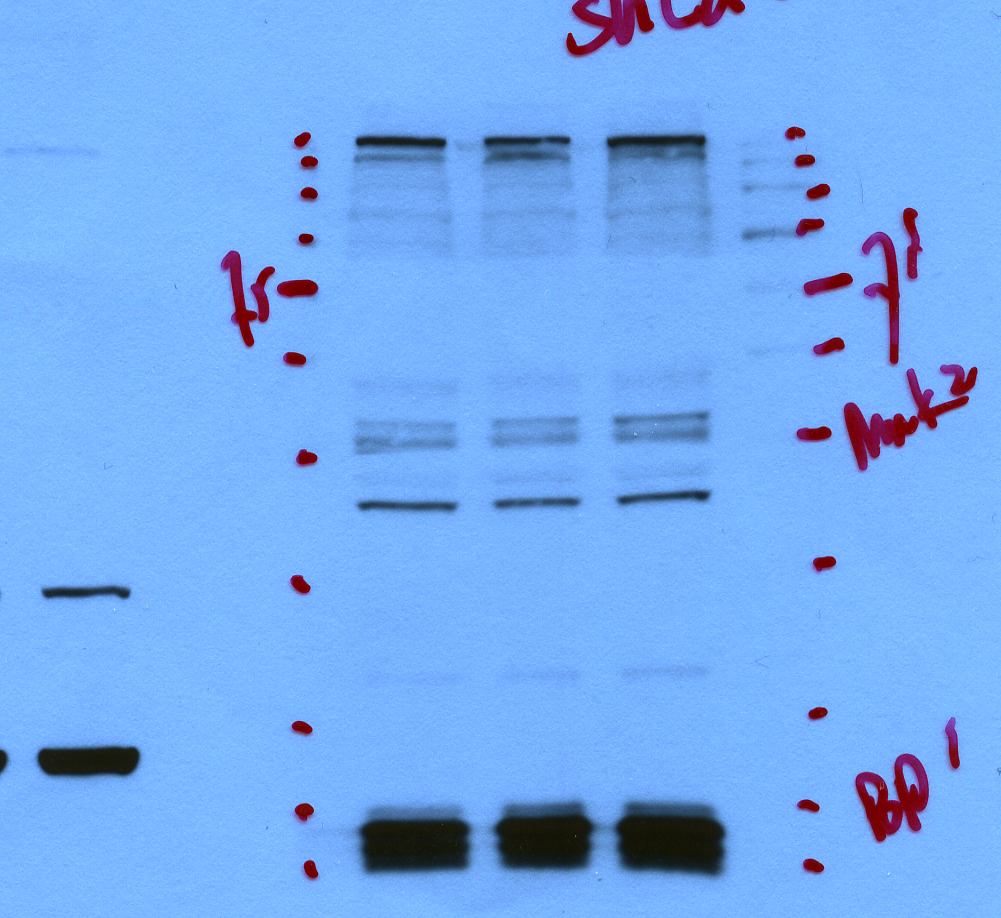

Supplement: Supplementary file 2 [file LSA-2024-02755_SdataFS1.zip › LSA-2024-02755_SdataFS1.34.tif]

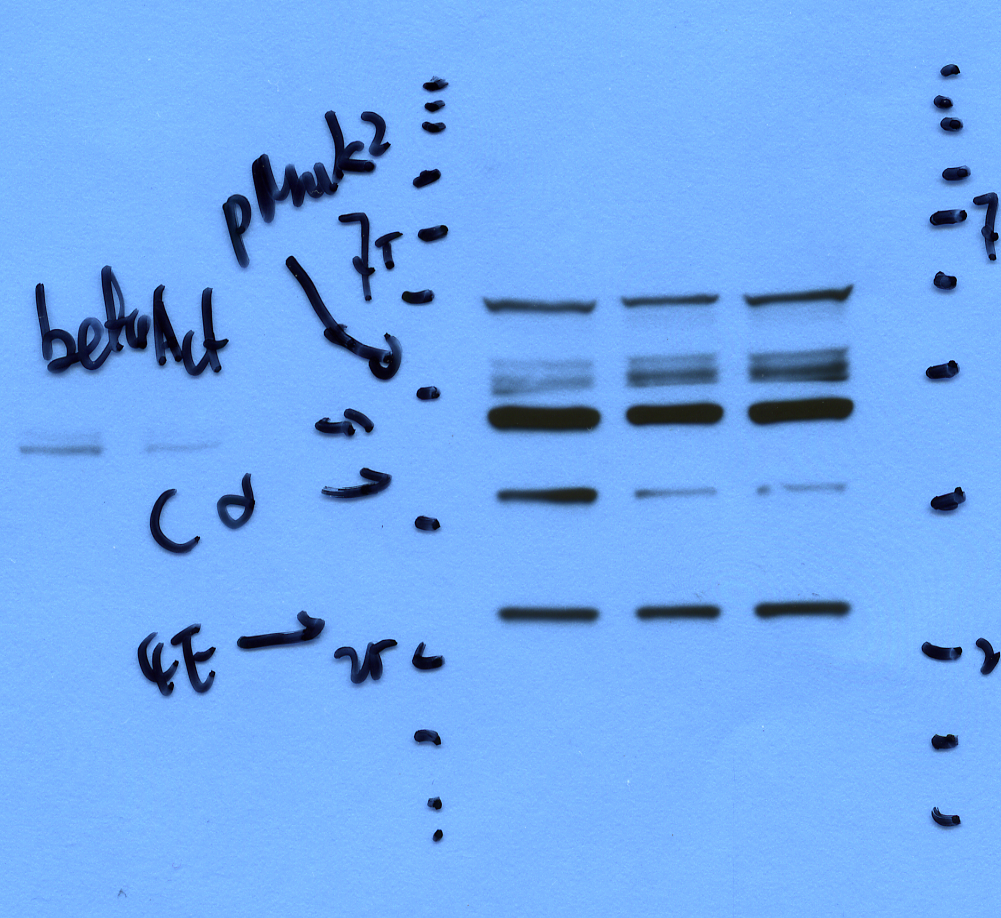

Supplement: Supplementary file 2 [file LSA-2024-02755_SdataFS1.zip › LSA-2024-02755_SdataFS1.35.tif]

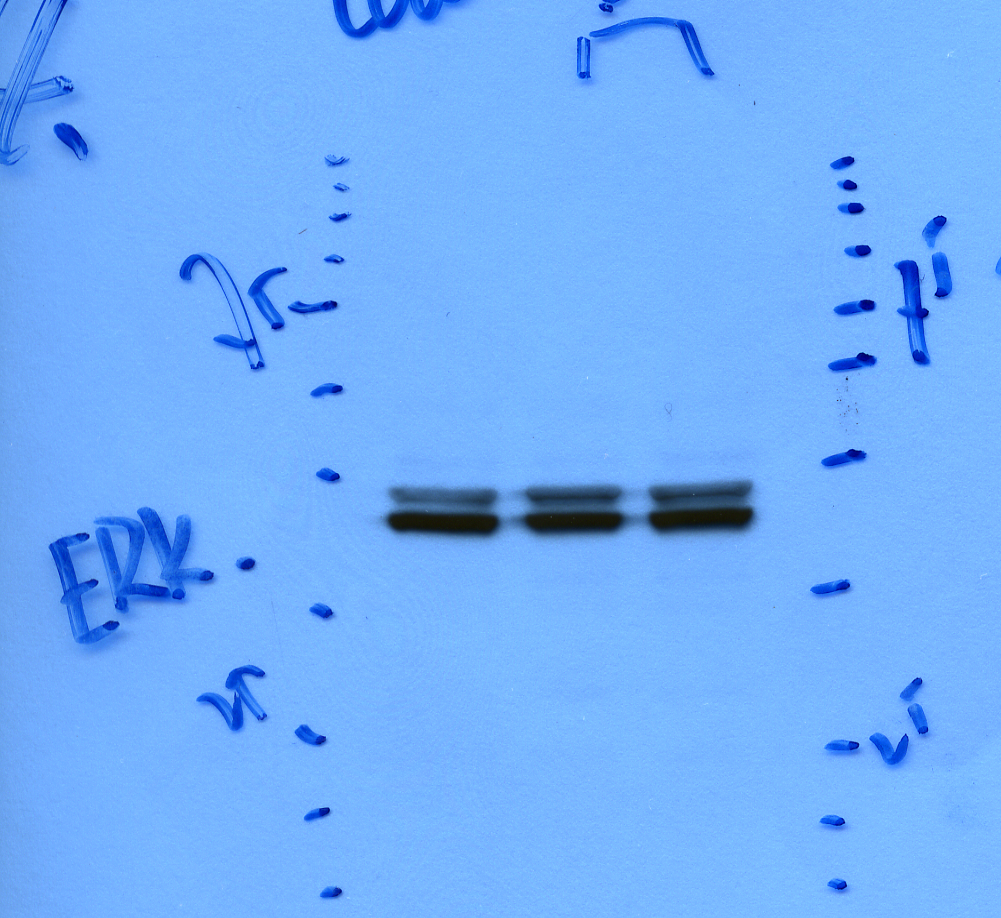

Supplement: Supplementary file 2 [file LSA-2024-02755_SdataFS1.zip › LSA-2024-02755_SdataFS1.36.tif]

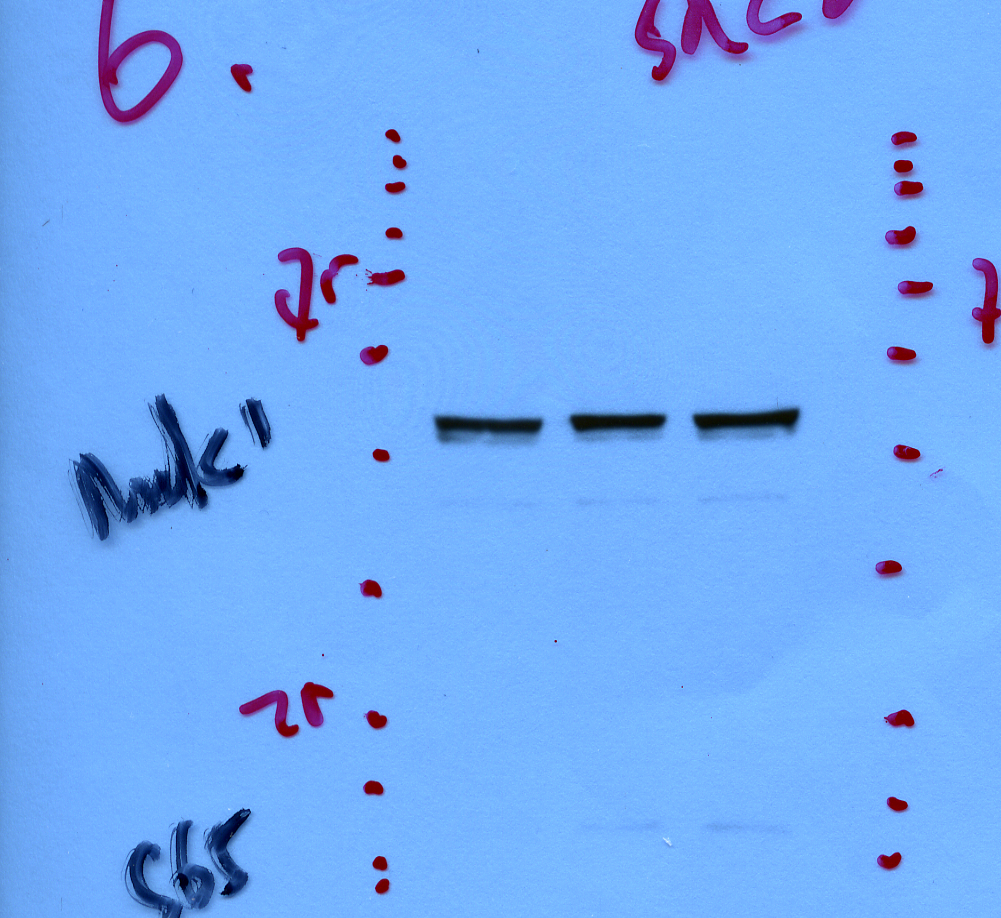

Supplement: Supplementary file 2 [file LSA-2024-02755_SdataFS1.zip › LSA-2024-02755_SdataFS1.37.tif]

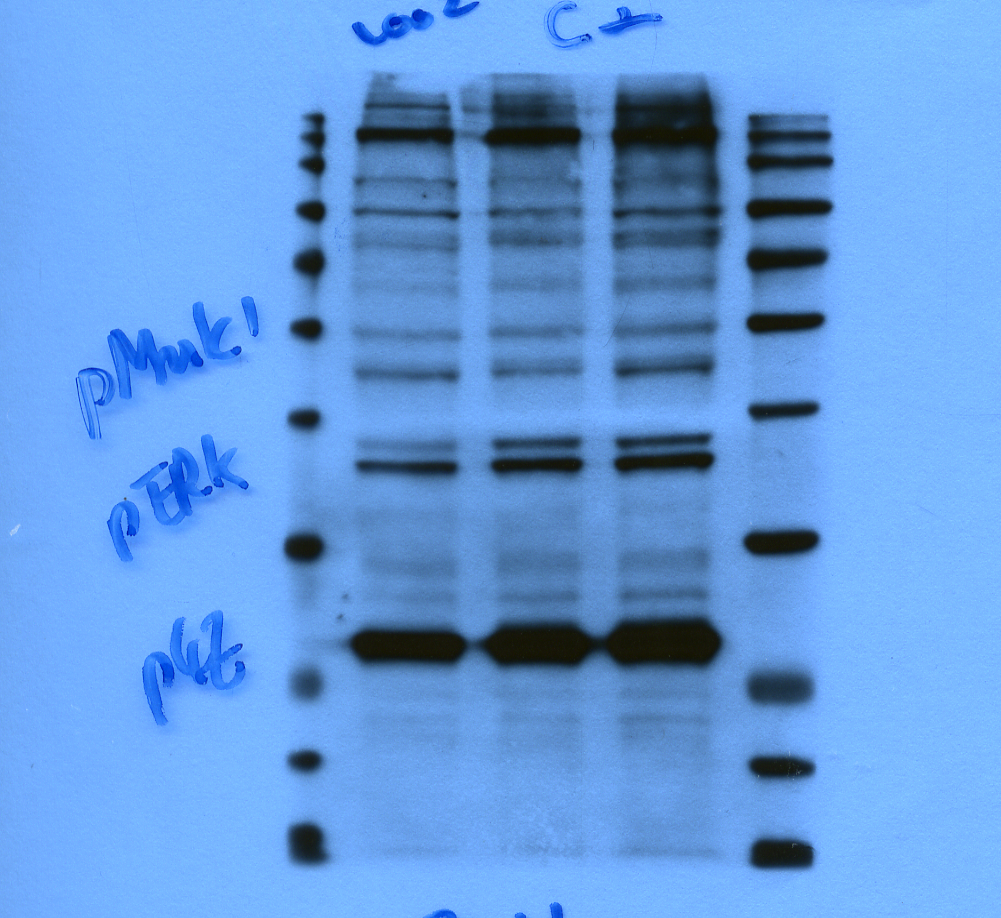

Supplement: Supplementary file 2 [file LSA-2024-02755_SdataFS1.zip › LSA-2024-02755_SdataFS1.38.tif]

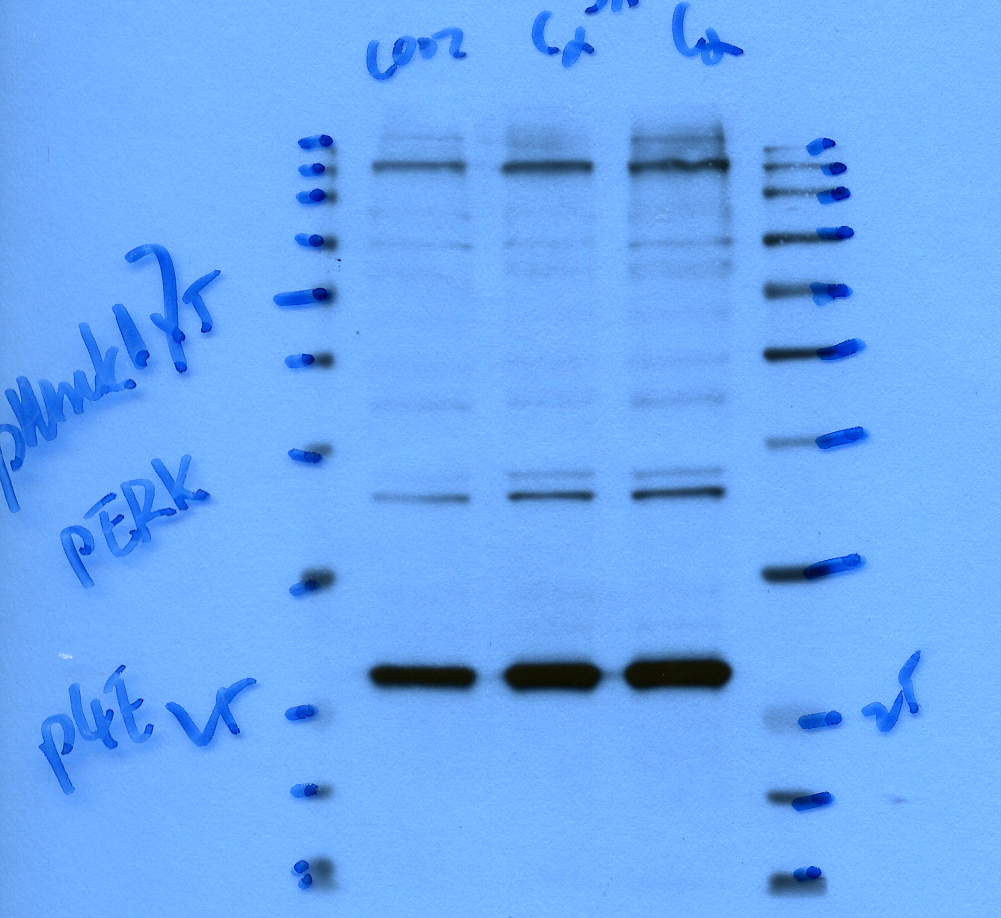

Supplement: Supplementary file 2 [file LSA-2024-02755_SdataFS1.zip › LSA-2024-02755_SdataFS1.39.tif]

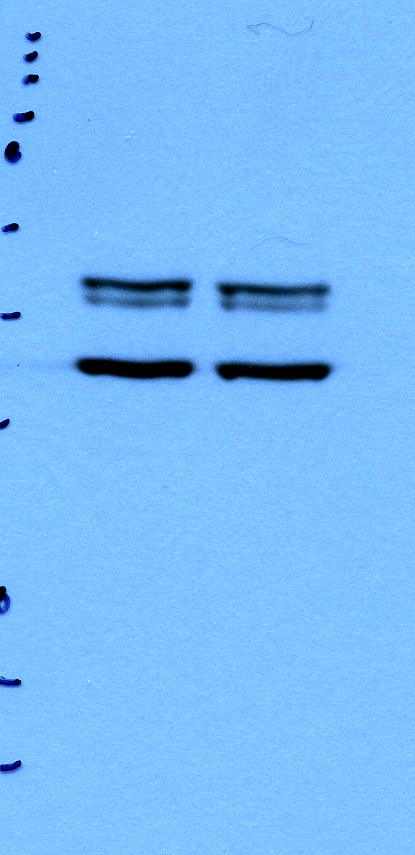

Supplement: Supplementary file 2 [file LSA-2024-02755_SdataFS1.zip › LSA-2024-02755_SdataFS1.4.tif]

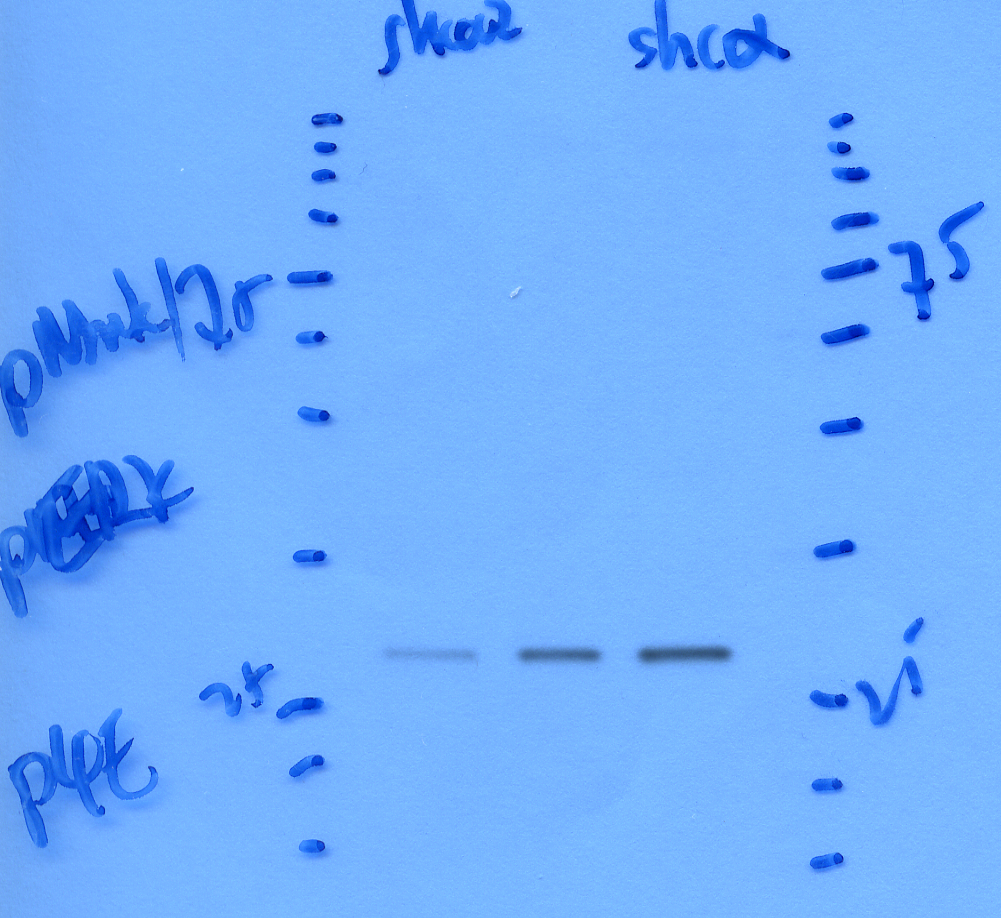

Supplement: Supplementary file 2 [file LSA-2024-02755_SdataFS1.zip › LSA-2024-02755_SdataFS1.40.tif]

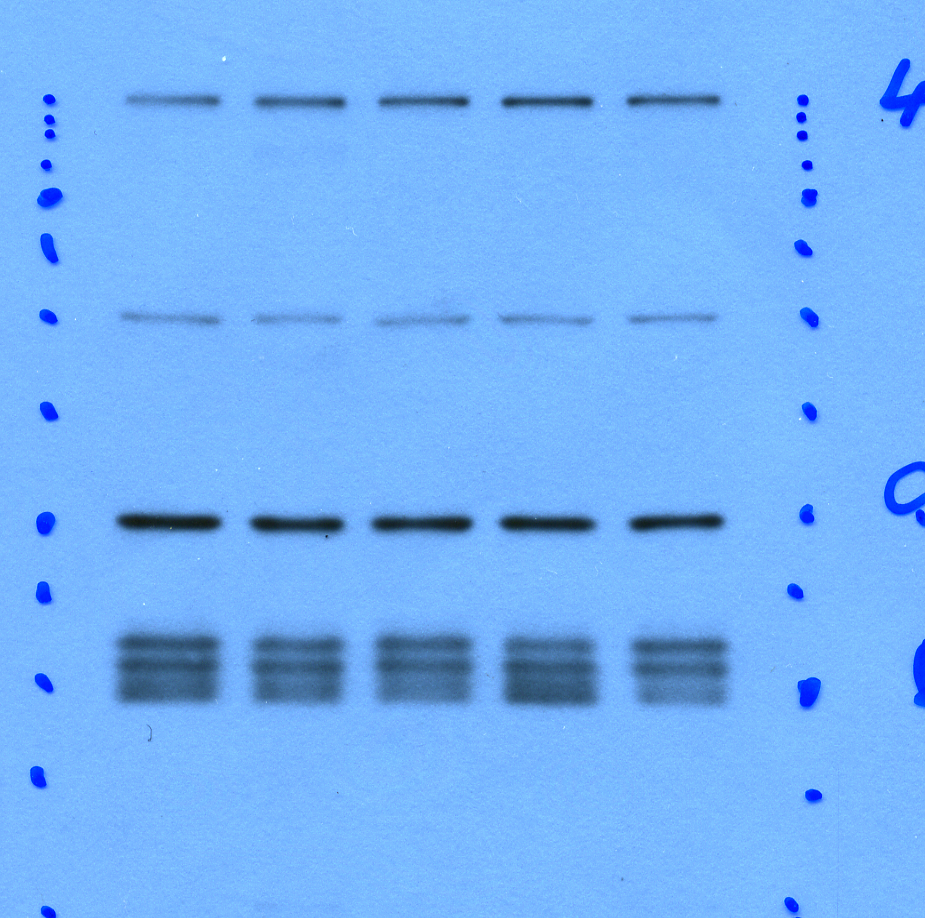

Supplement: Supplementary file 2 [file LSA-2024-02755_SdataFS1.zip › LSA-2024-02755_SdataFS1.41.tif]

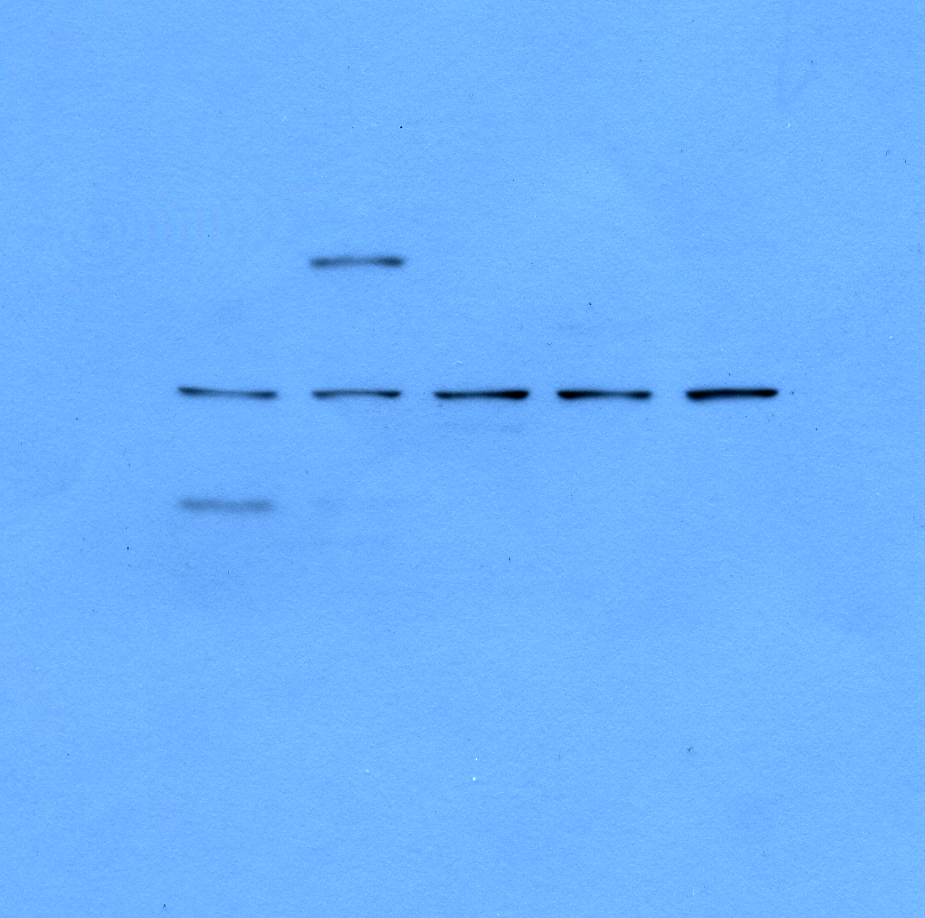

Supplement: Supplementary file 2 [file LSA-2024-02755_SdataFS1.zip › LSA-2024-02755_SdataFS1.42.tif]

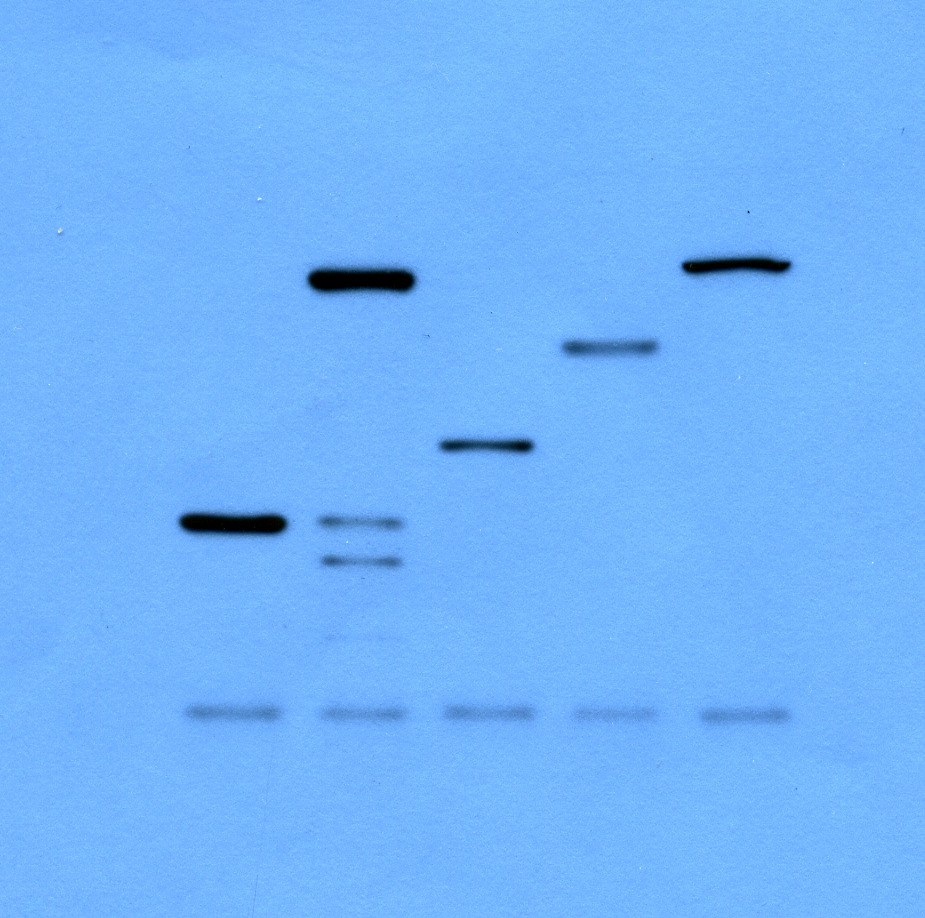

Supplement: Supplementary file 2 [file LSA-2024-02755_SdataFS1.zip › LSA-2024-02755_SdataFS1.43.tif]

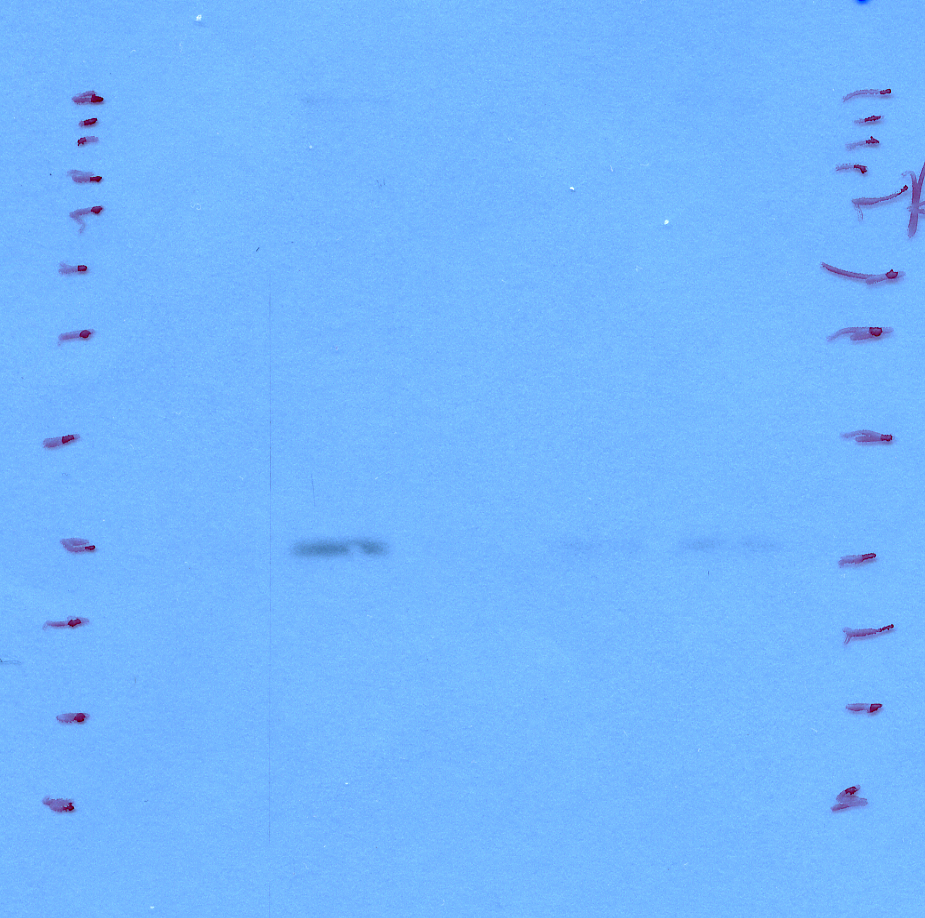

Supplement: Supplementary file 2 [file LSA-2024-02755_SdataFS1.zip › LSA-2024-02755_SdataFS1.44.tif]

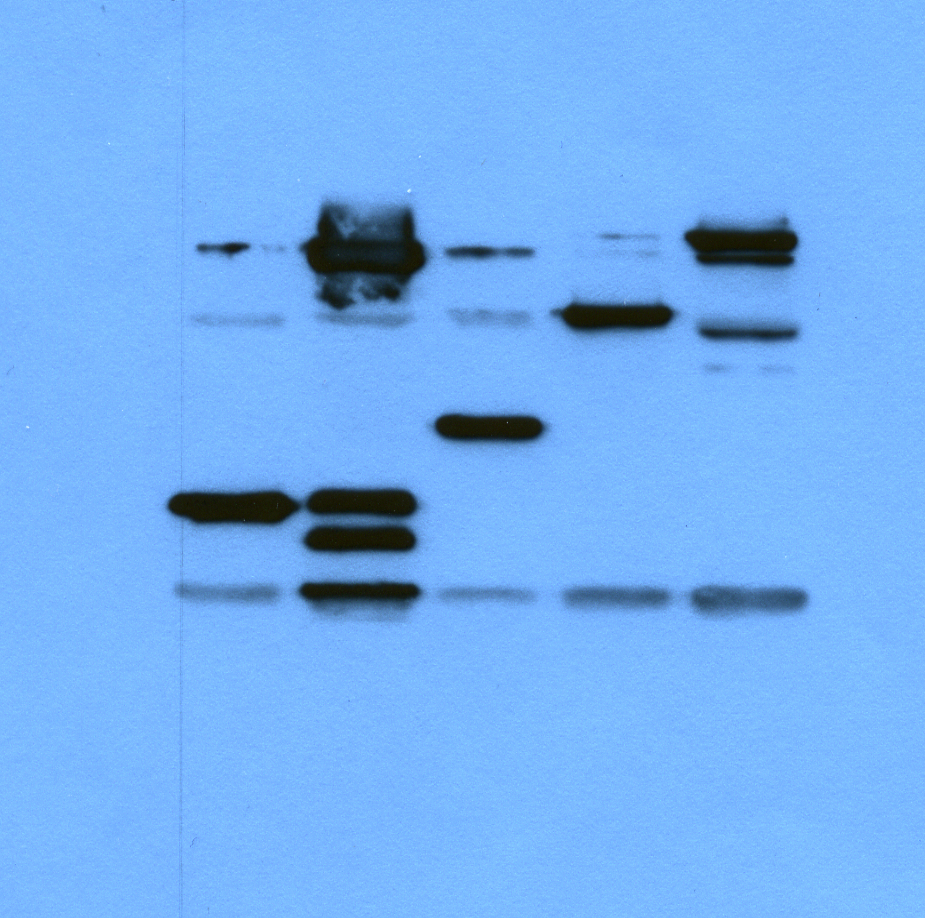

Supplement: Supplementary file 2 [file LSA-2024-02755_SdataFS1.zip › LSA-2024-02755_SdataFS1.45.tif]

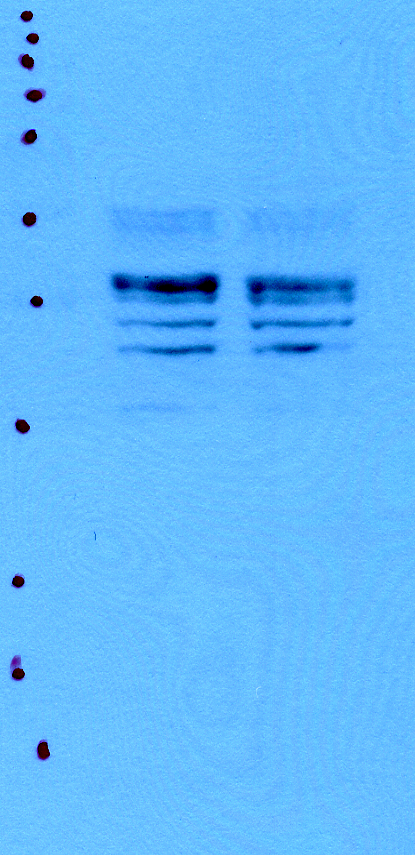

Supplement: Supplementary file 2 [file LSA-2024-02755_SdataFS1.zip › LSA-2024-02755_SdataFS1.5.tif]

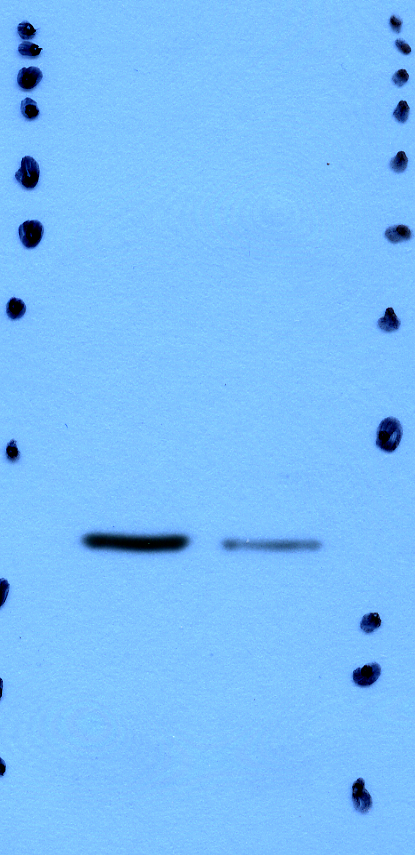

Supplement: Supplementary file 2 [file LSA-2024-02755_SdataFS1.zip › LSA-2024-02755_SdataFS1.6.tif]

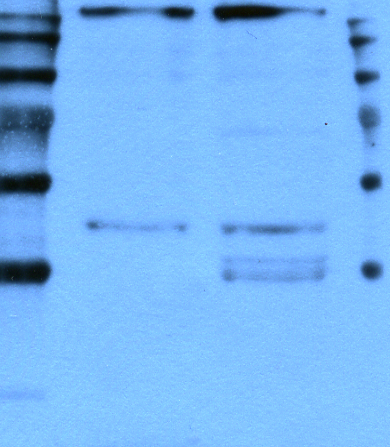

Supplement: Supplementary file 2 [file LSA-2024-02755_SdataFS1.zip › LSA-2024-02755_SdataFS1.7.tif]

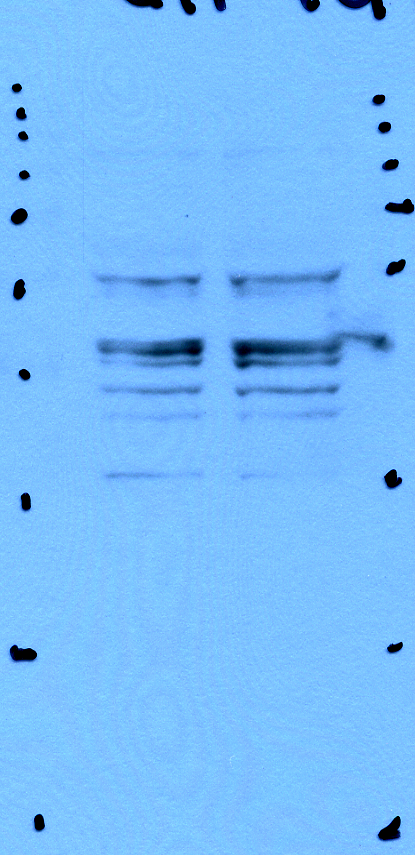

Supplement: Supplementary file 2 [file LSA-2024-02755_SdataFS1.zip › LSA-2024-02755_SdataFS1.8.tif]

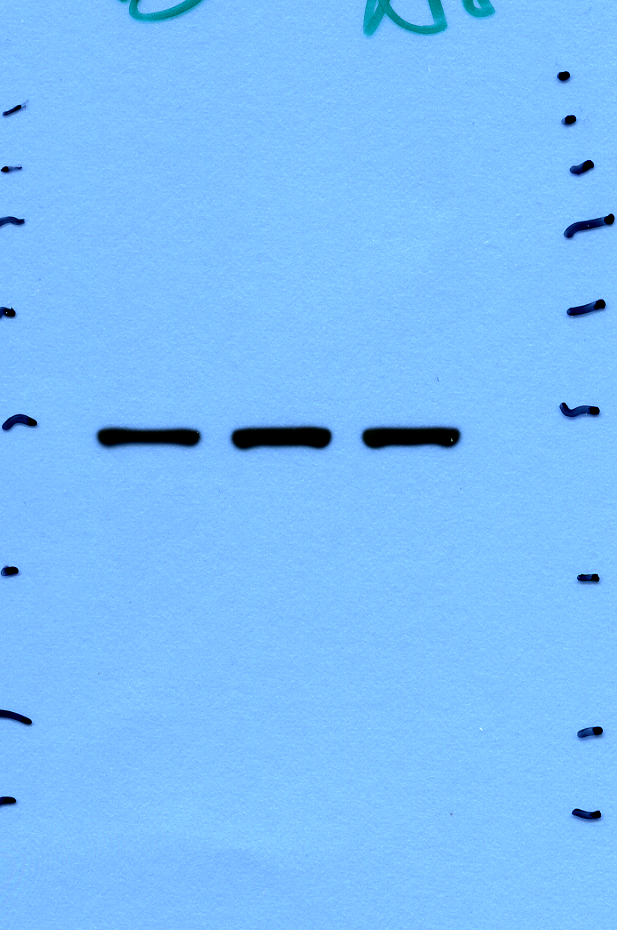

Supplement: Supplementary file 2 [file LSA-2024-02755_SdataFS1.zip › LSA-2024-02755_SdataFS1.9.tif]

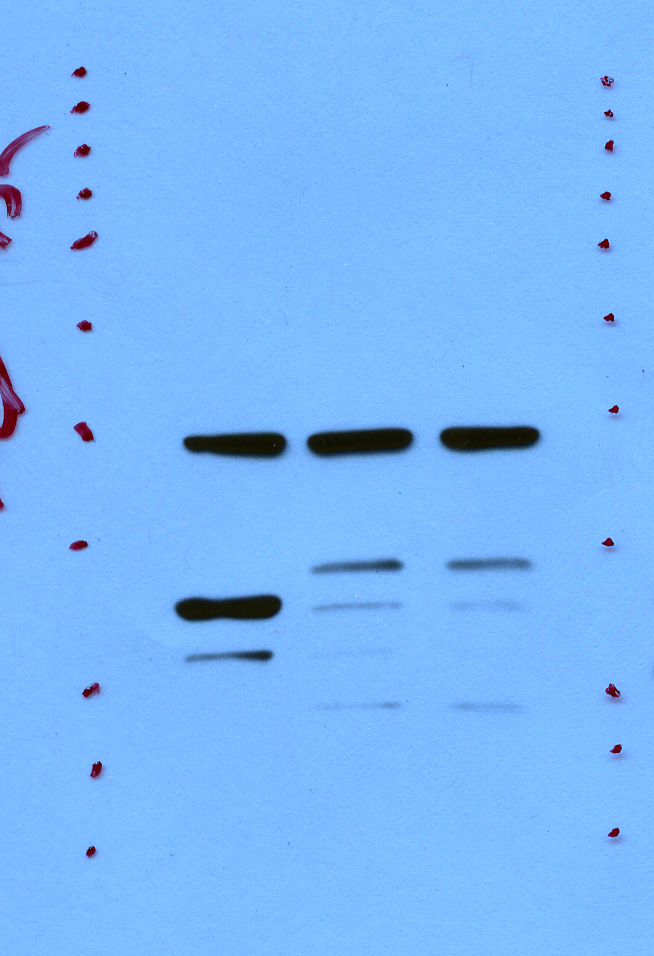

Supplement: Supplementary file 3 [file LSA-2024-02755_SdataF2.zip › LSA-2024-02755_SdataF2.1.tif]

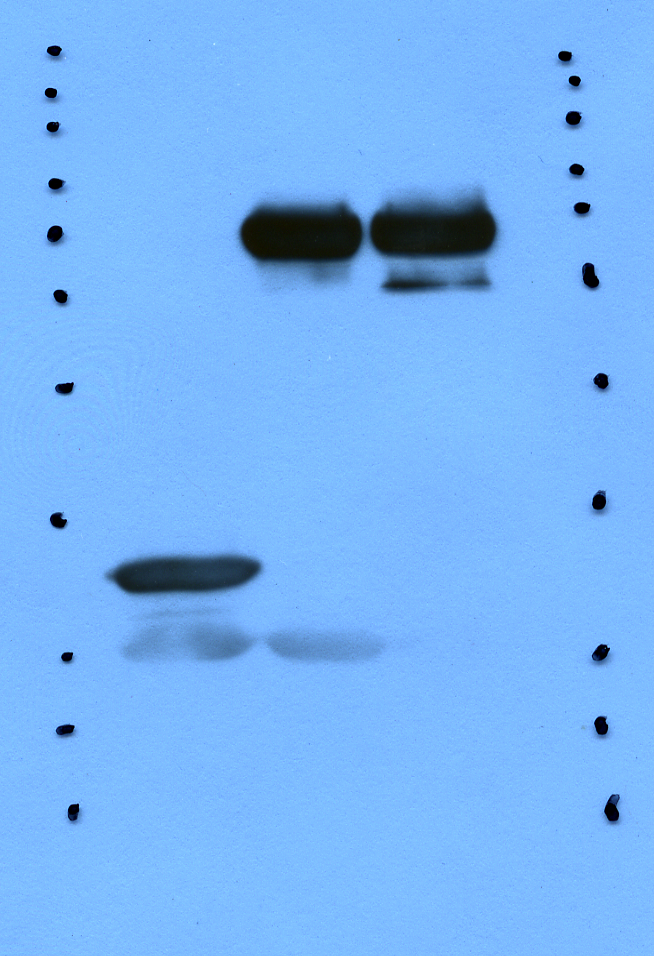

Supplement: Supplementary file 3 [file LSA-2024-02755_SdataF2.zip › LSA-2024-02755_SdataF2.10.tif]

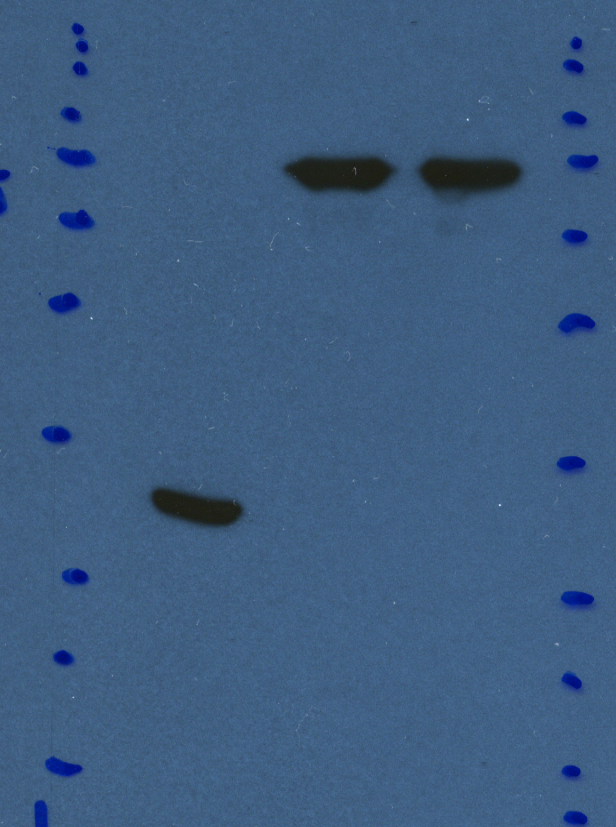

Supplement: Supplementary file 3 [file LSA-2024-02755_SdataF2.zip › LSA-2024-02755_SdataF2.12.tif]

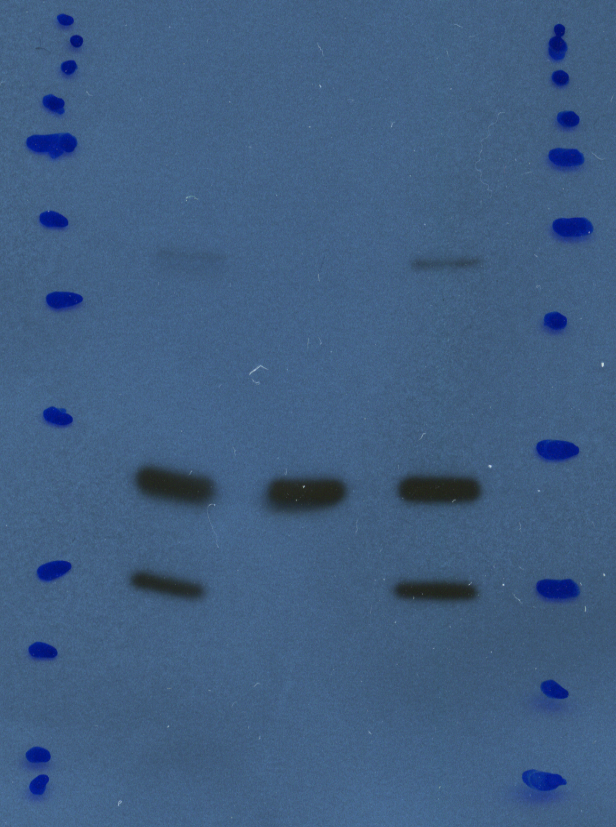

Supplement: Supplementary file 3 [file LSA-2024-02755_SdataF2.zip › LSA-2024-02755_SdataF2.13.tif]

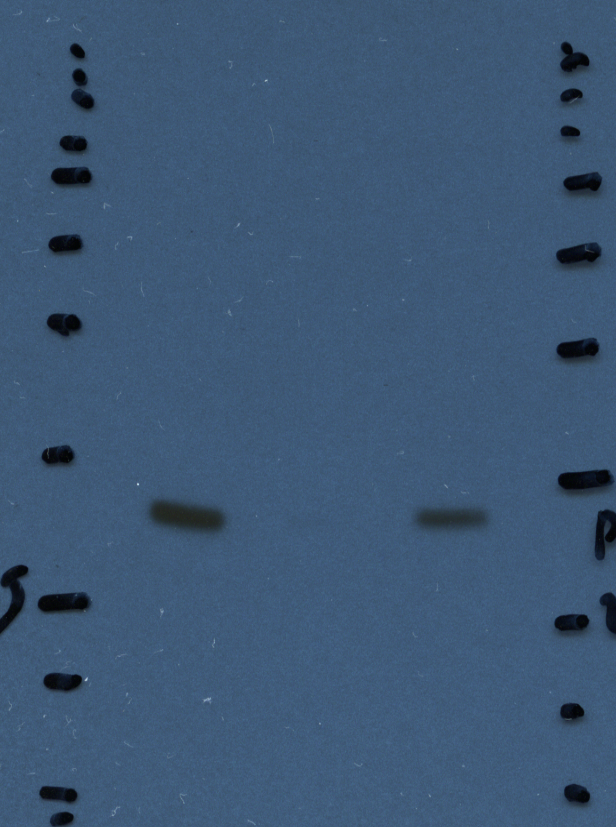

Supplement: Supplementary file 3 [file LSA-2024-02755_SdataF2.zip › LSA-2024-02755_SdataF2.14.tif]

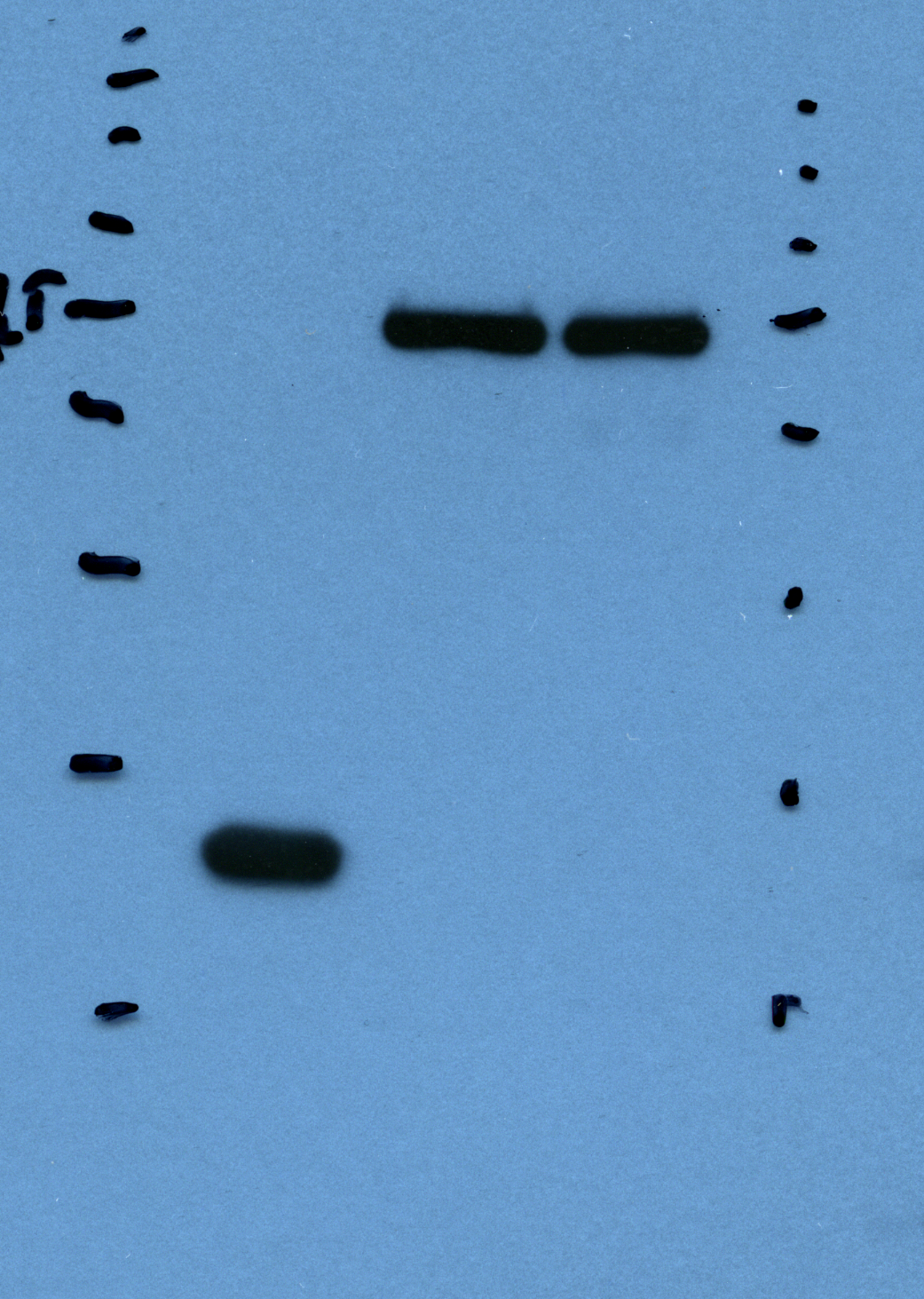

Supplement: Supplementary file 3 [file LSA-2024-02755_SdataF2.zip › LSA-2024-02755_SdataF2.15.tif]

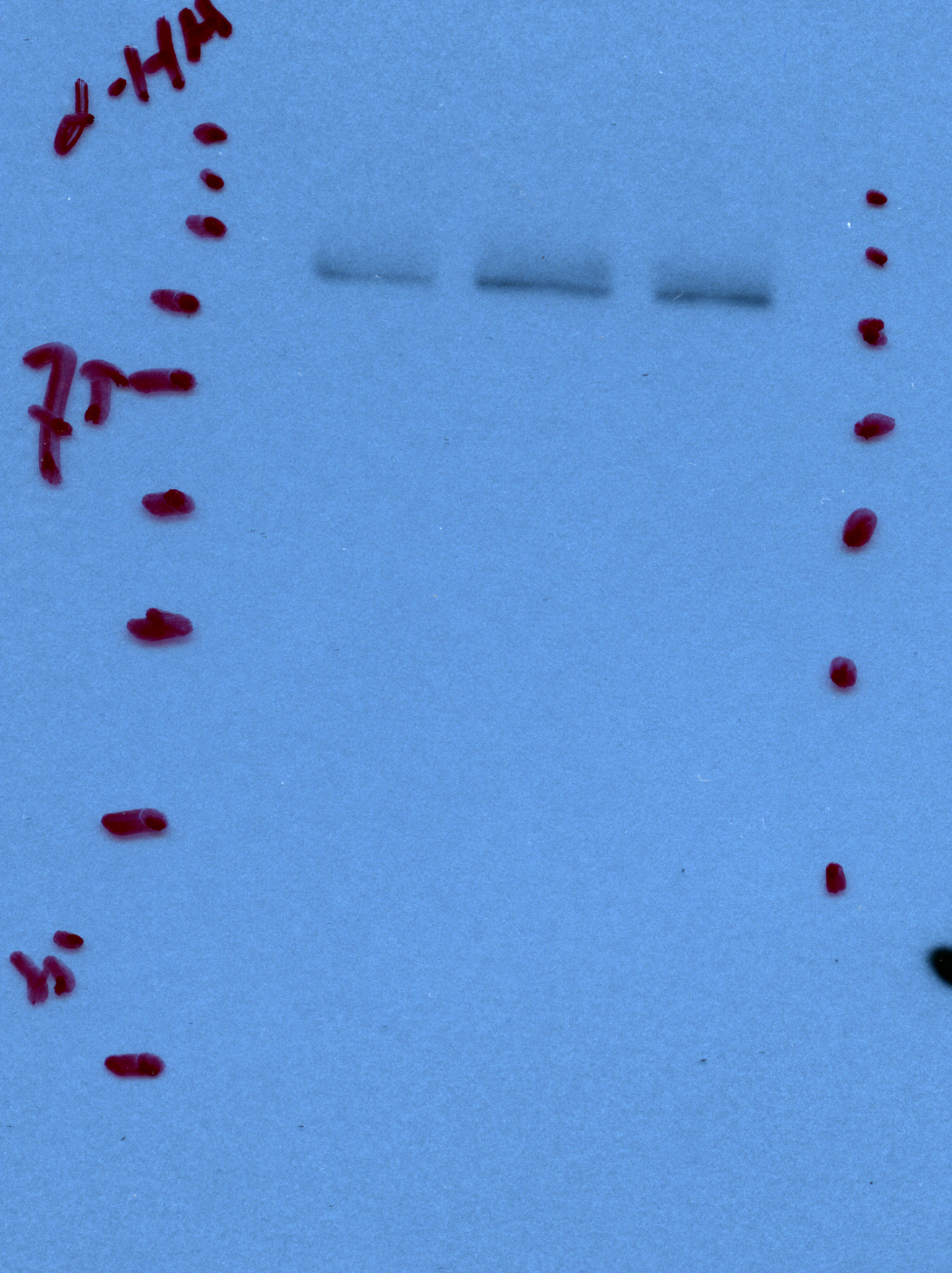

Supplement: Supplementary file 3 [file LSA-2024-02755_SdataF2.zip › LSA-2024-02755_SdataF2.16.tif]

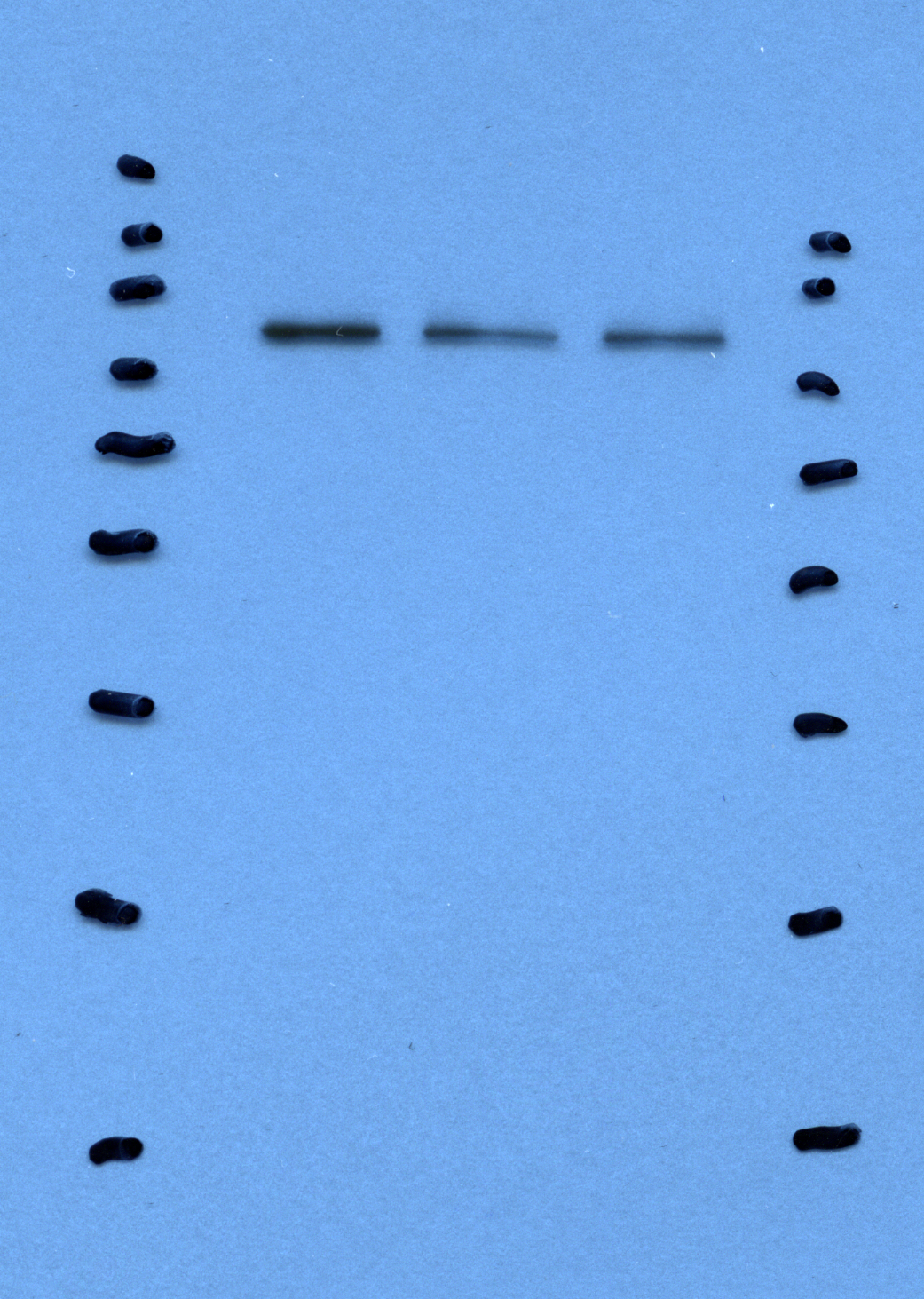

Supplement: Supplementary file 3 [file LSA-2024-02755_SdataF2.zip › LSA-2024-02755_SdataF2.17.tif]

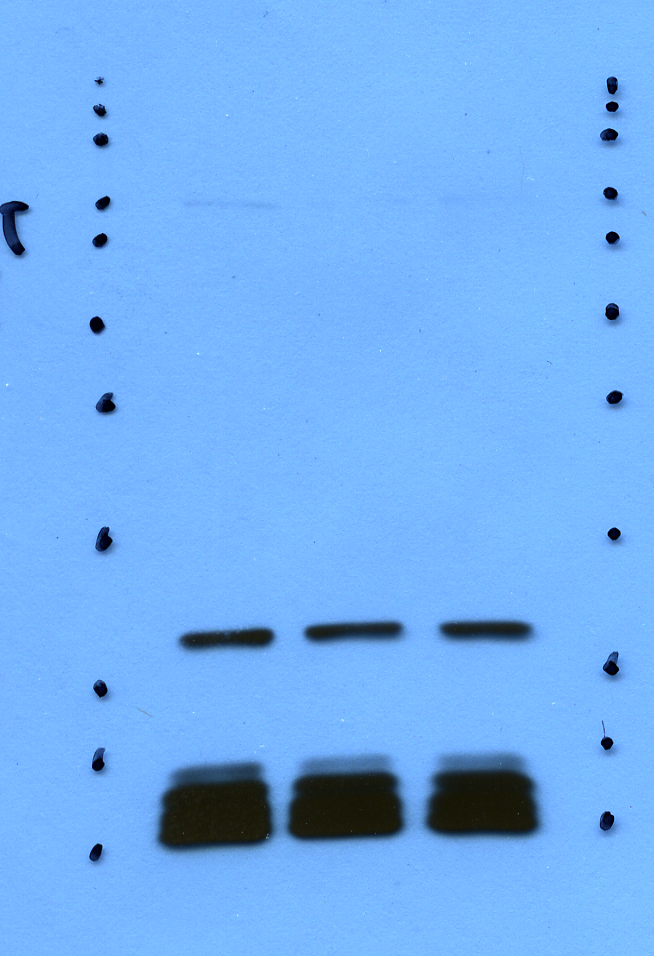

Supplement: Supplementary file 3 [file LSA-2024-02755_SdataF2.zip › LSA-2024-02755_SdataF2.2.tif]

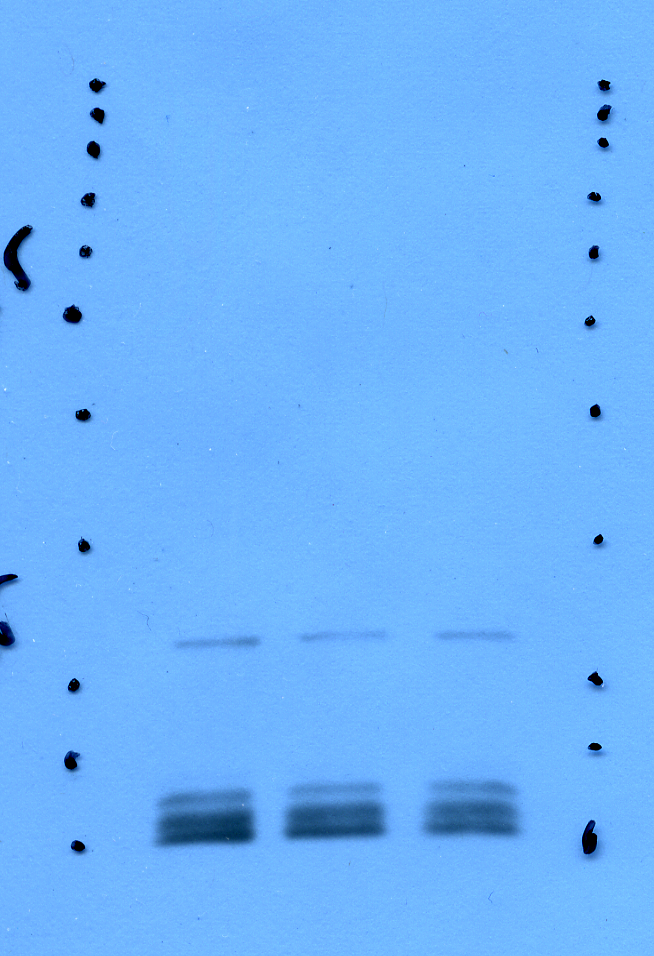

Supplement: Supplementary file 3 [file LSA-2024-02755_SdataF2.zip › LSA-2024-02755_SdataF2.3.tif]

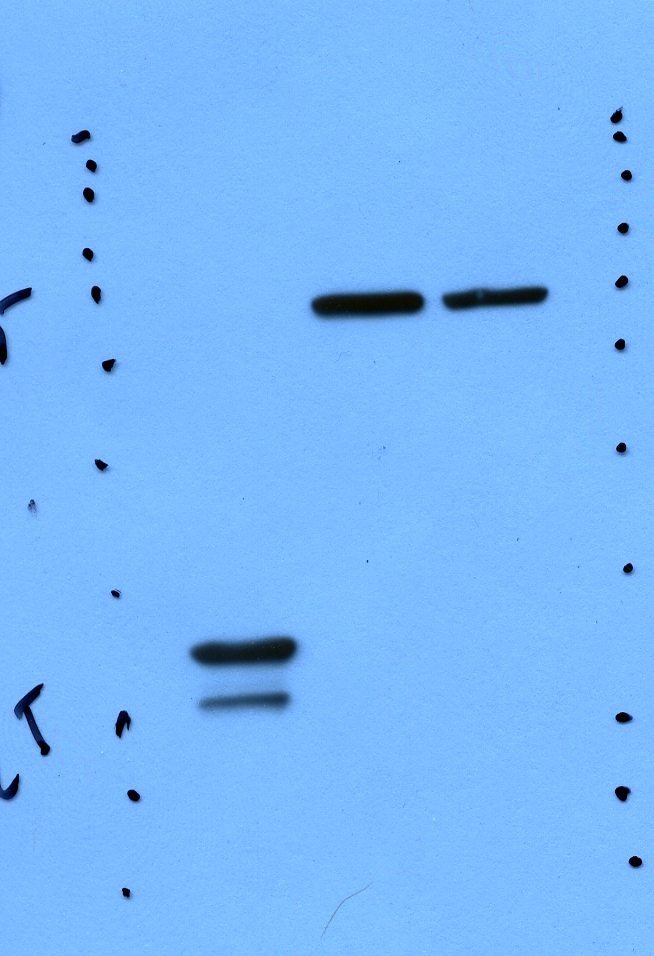

Supplement: Supplementary file 3 [file LSA-2024-02755_SdataF2.zip › LSA-2024-02755_SdataF2.4.tif]

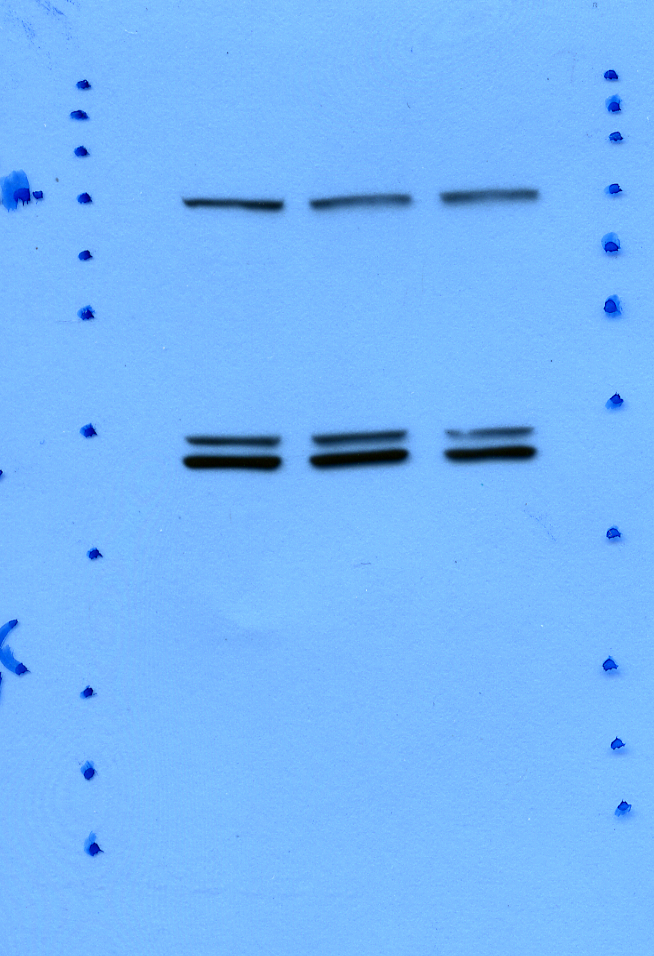

Supplement: Supplementary file 3 [file LSA-2024-02755_SdataF2.zip › LSA-2024-02755_SdataF2.5.tif]

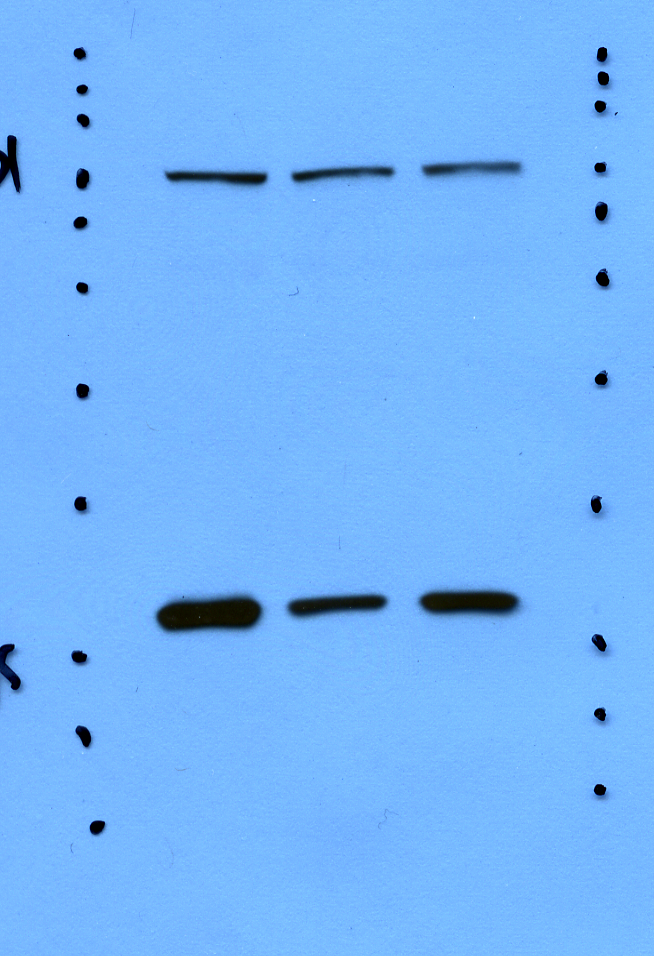

Supplement: Supplementary file 3 [file LSA-2024-02755_SdataF2.zip › LSA-2024-02755_SdataF2.6.tif]

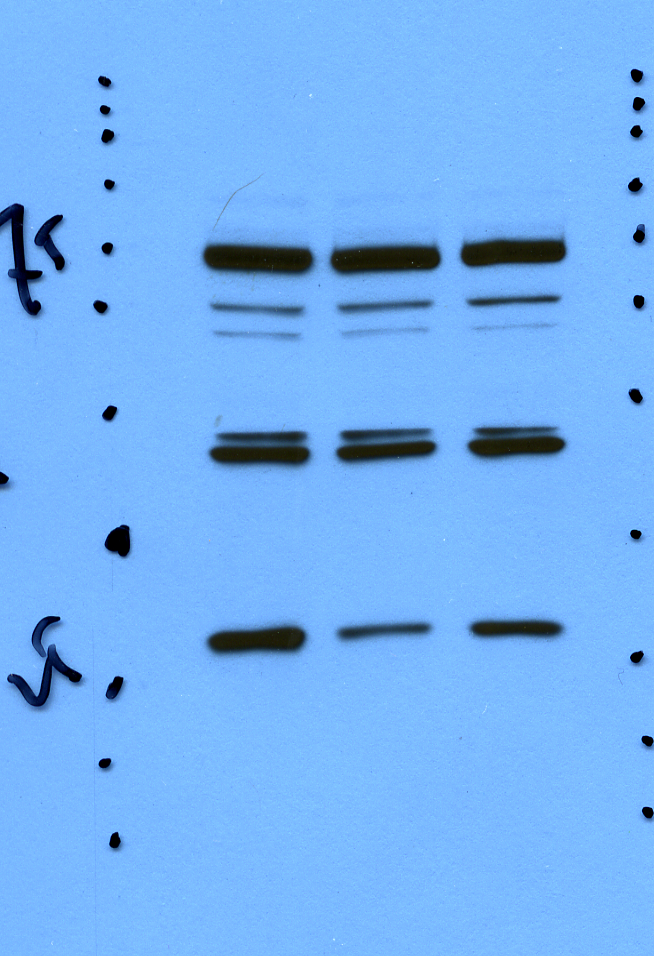

Supplement: Supplementary file 3 [file LSA-2024-02755_SdataF2.zip › LSA-2024-02755_SdataF2.7.tif]

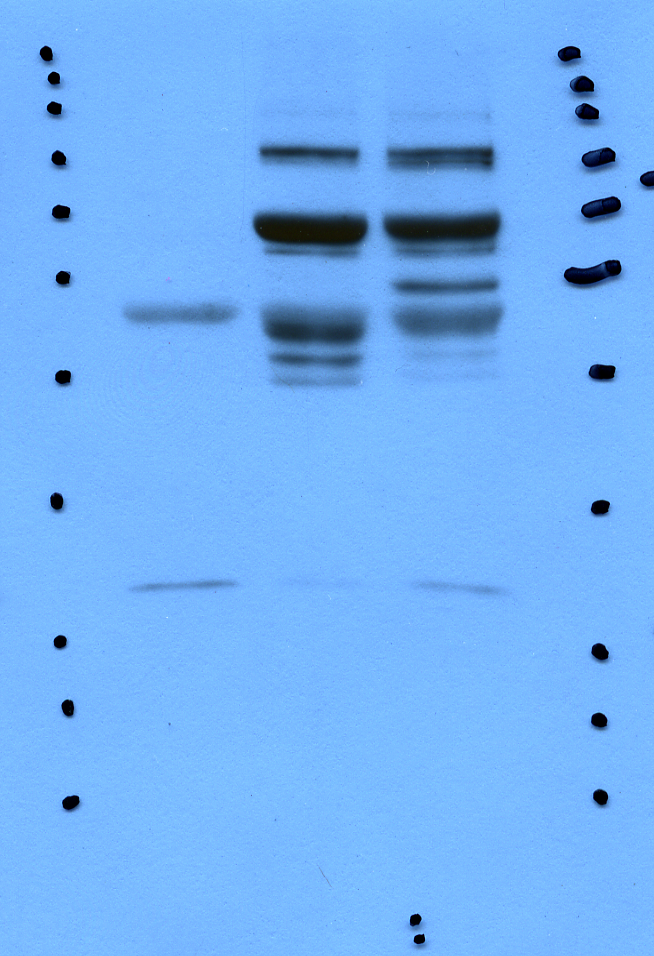

Supplement: Supplementary file 3 [file LSA-2024-02755_SdataF2.zip › LSA-2024-02755_SdataF2.8.tif]

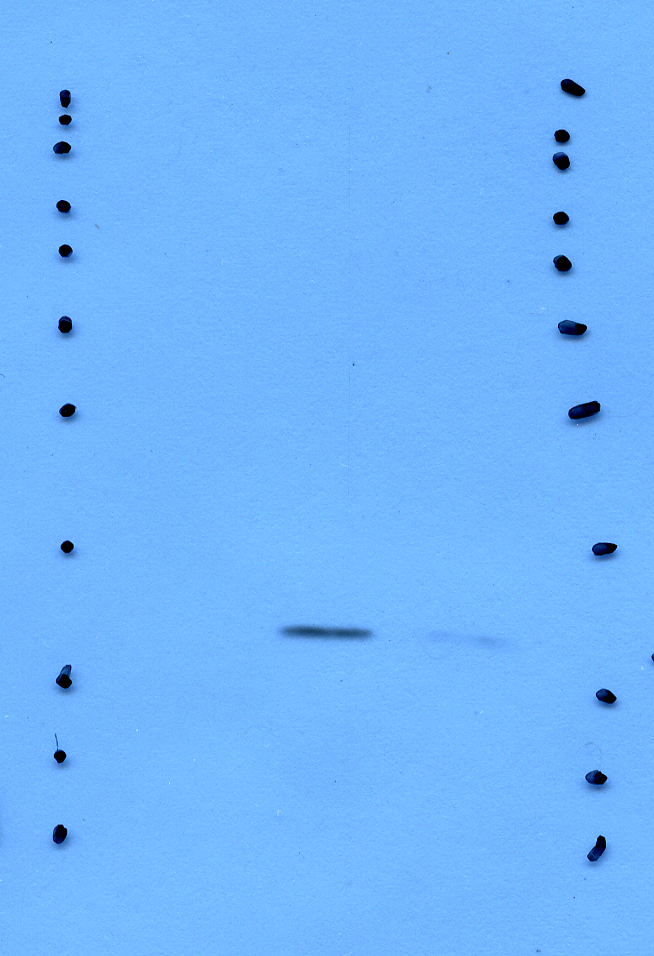

Supplement: Supplementary file 3 [file LSA-2024-02755_SdataF2.zip › LSA-2024-02755_SdataF2.9.tif]

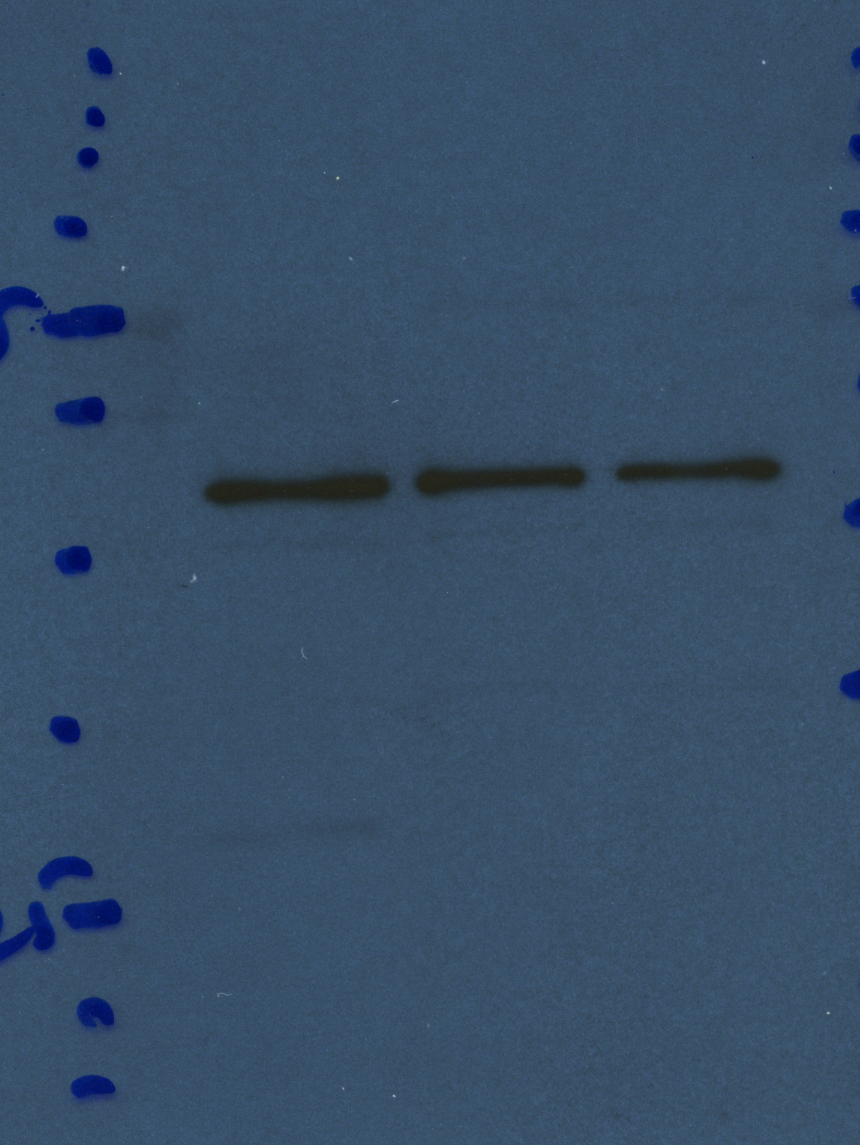

Supplement: Supplementary file 4 [file LSA-2024-02755_SdataFS2.zip › LSA-2024-02755_SdataFS2.2.tif]

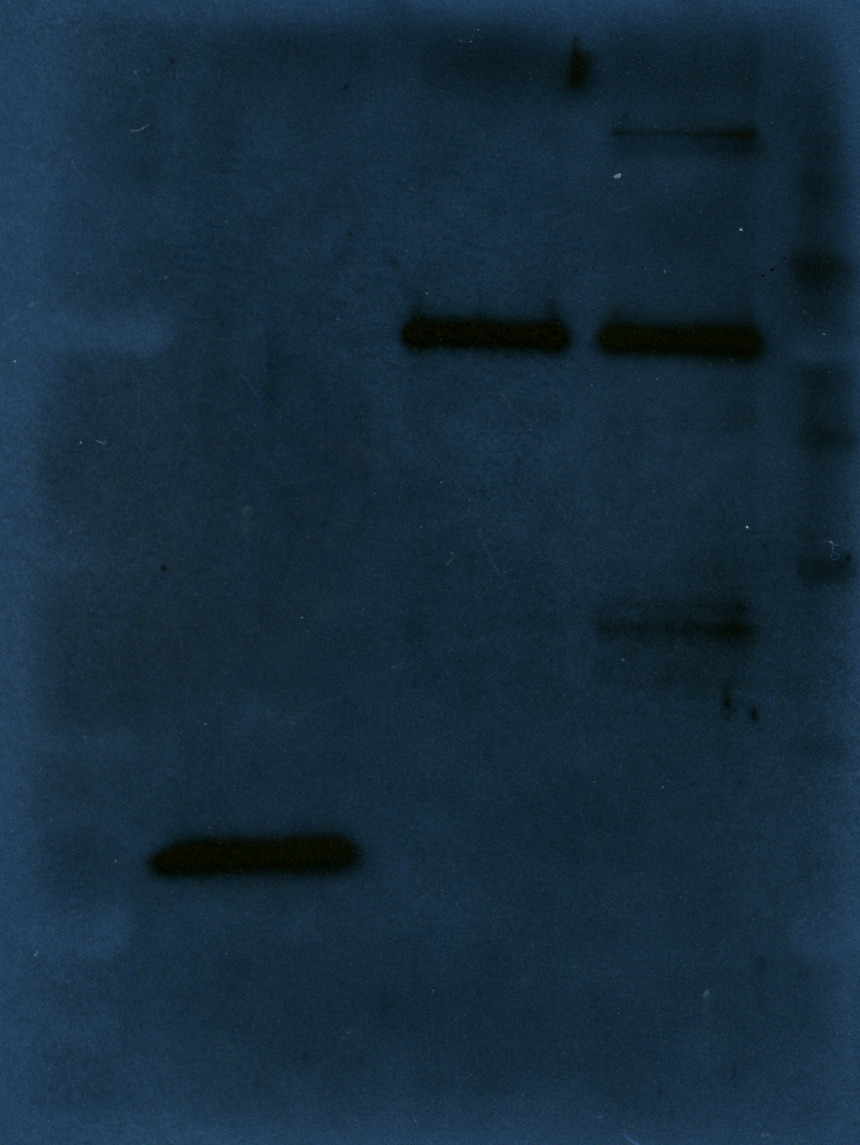

Supplement: Supplementary file 4 [file LSA-2024-02755_SdataFS2.zip › LSA-2024-02755_SdataFS2.3.tif]

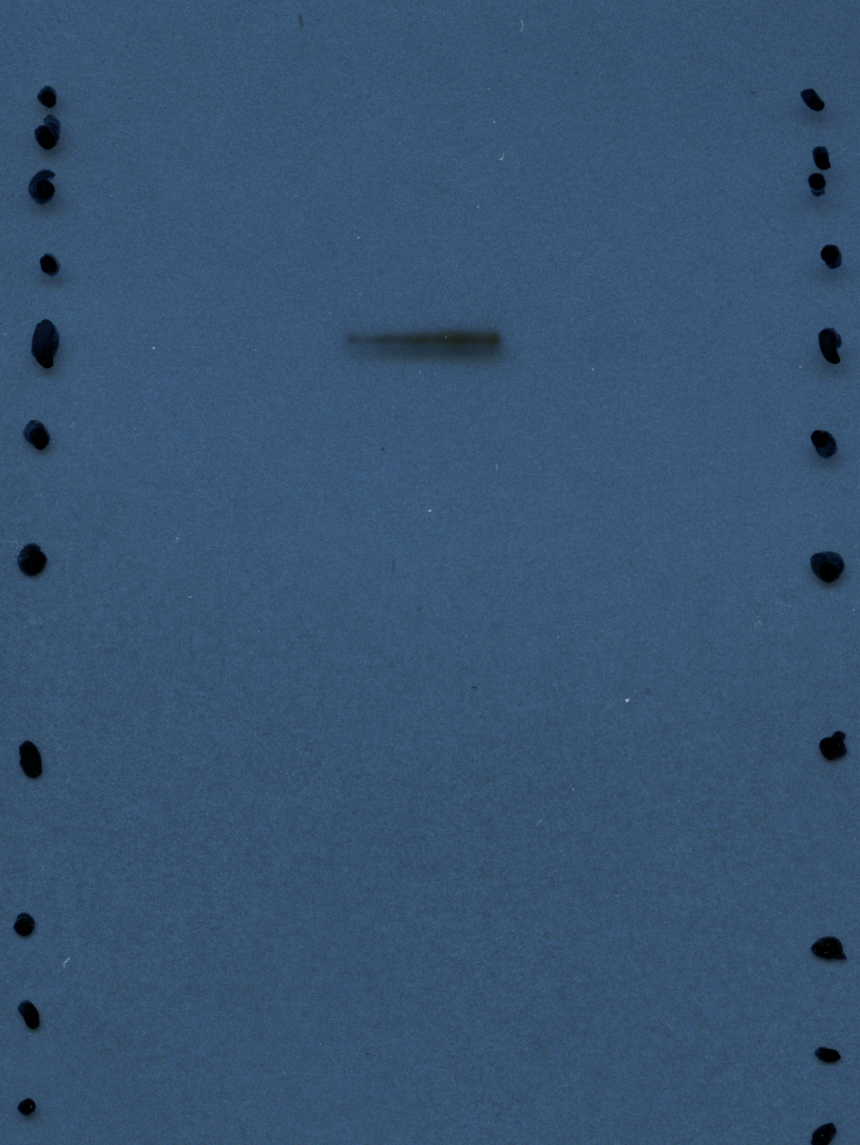

Supplement: Supplementary file 4 [file LSA-2024-02755_SdataFS2.zip › LSA-2024-02755_SdataFS2.4.tif]

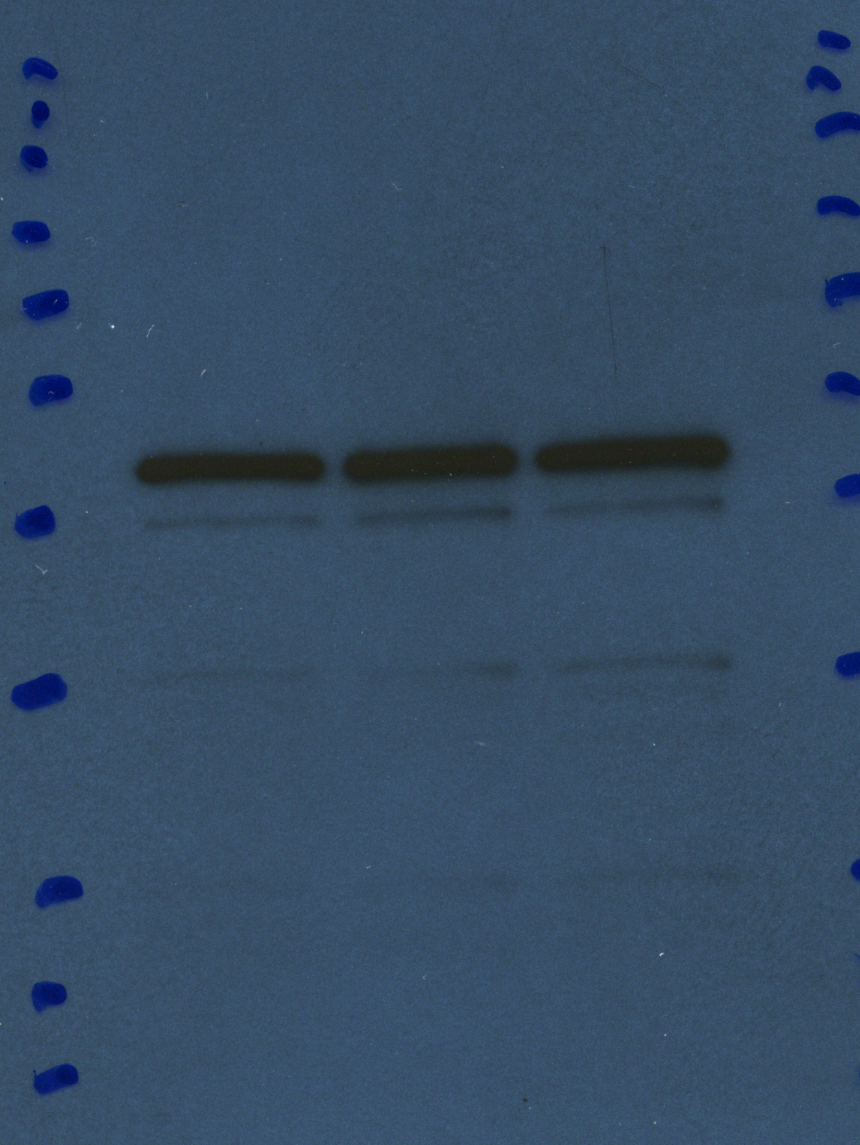

Supplement: Supplementary file 4 [file LSA-2024-02755_SdataFS2.zip › LSA-2024-02755_SdataFS2.5.tif]

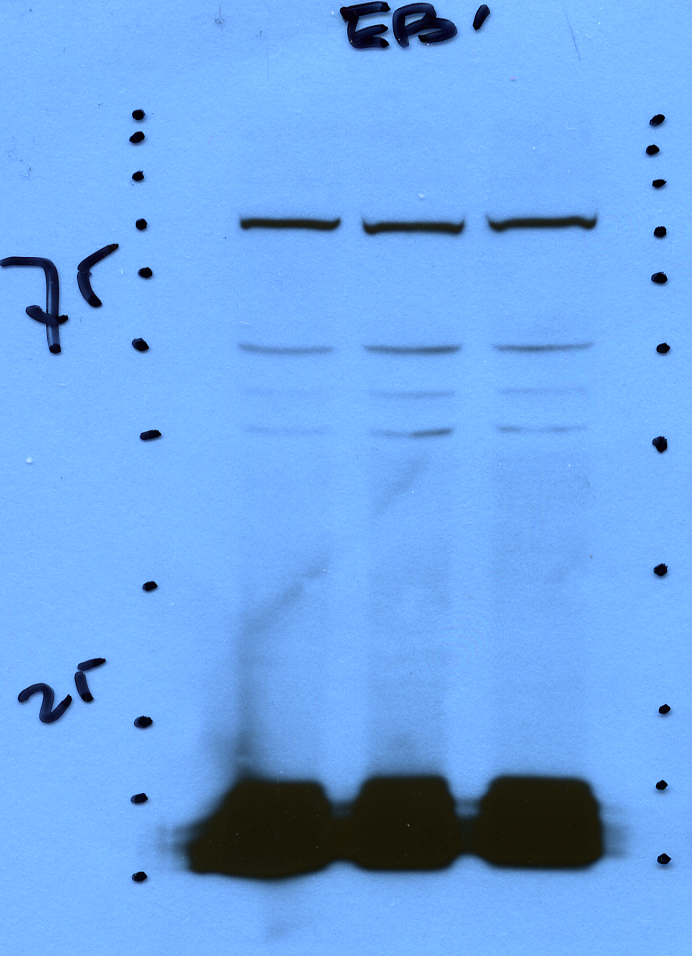

Supplement: Supplementary file 5 [file LSA-2024-02755_SdataF3.zip › LSA-2024-02755_SdataF3.1.tif]

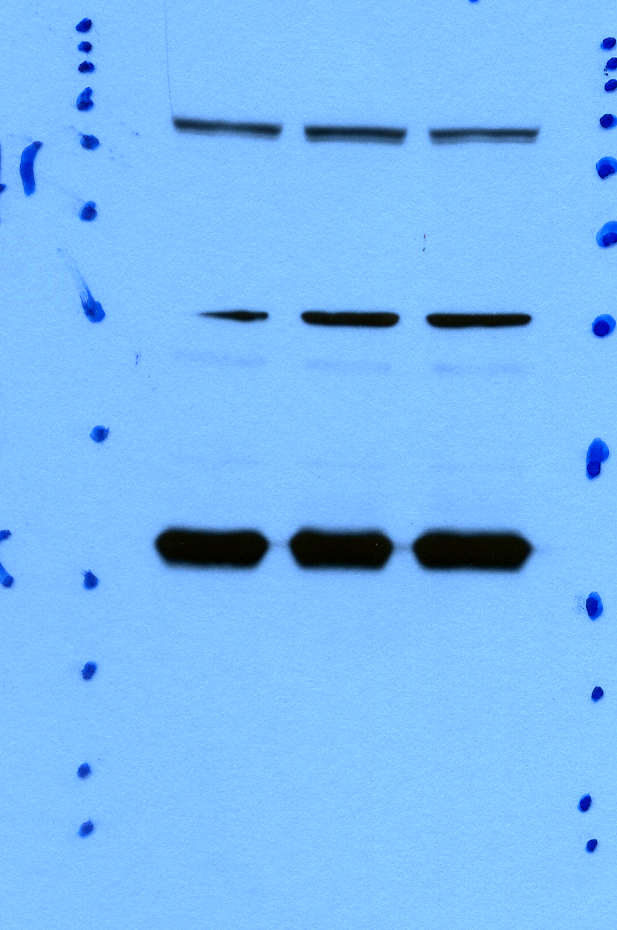

Supplement: Supplementary file 5 [file LSA-2024-02755_SdataF3.zip › LSA-2024-02755_SdataF3.10.tif]

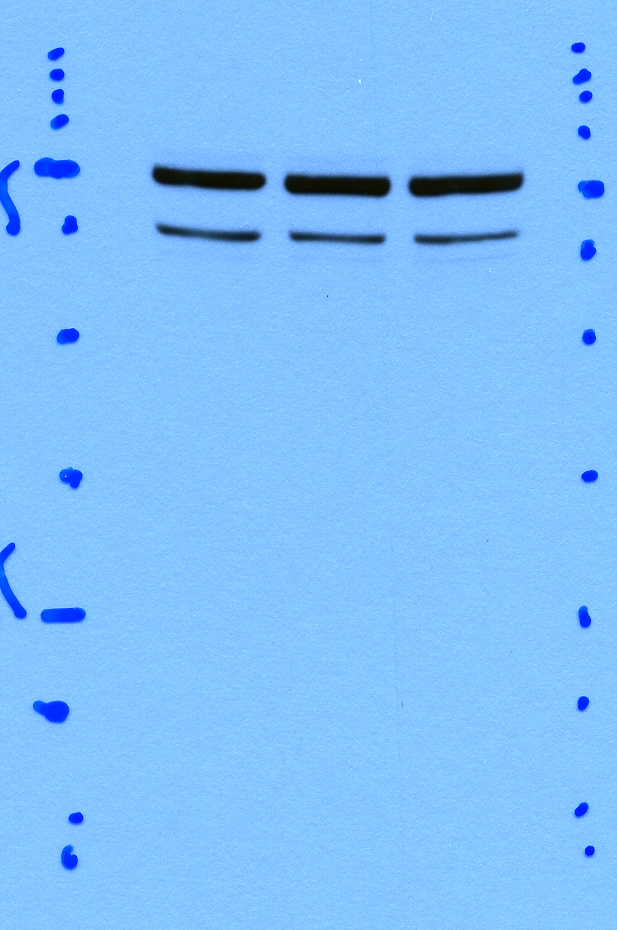

Supplement: Supplementary file 5 [file LSA-2024-02755_SdataF3.zip › LSA-2024-02755_SdataF3.11.tif]

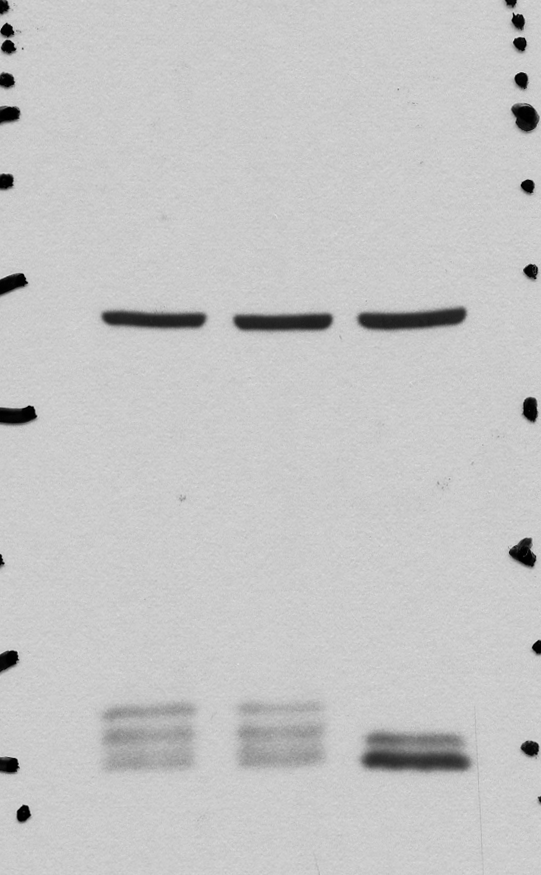

Supplement: Supplementary file 5 [file LSA-2024-02755_SdataF3.zip › LSA-2024-02755_SdataF3.12.tif]
