# Supplementary figures and images for: PPM1G dephosphorylates eIF4E in control of mRNA translation and cell proliferation (part 2 of 2)
Source: Life Sci Alliance. 2024 Aug 7;7(10):e202402755. doi: 10.26508/lsa.202402755 (PMC11306785; doi:10.26508/lsa.202402755)

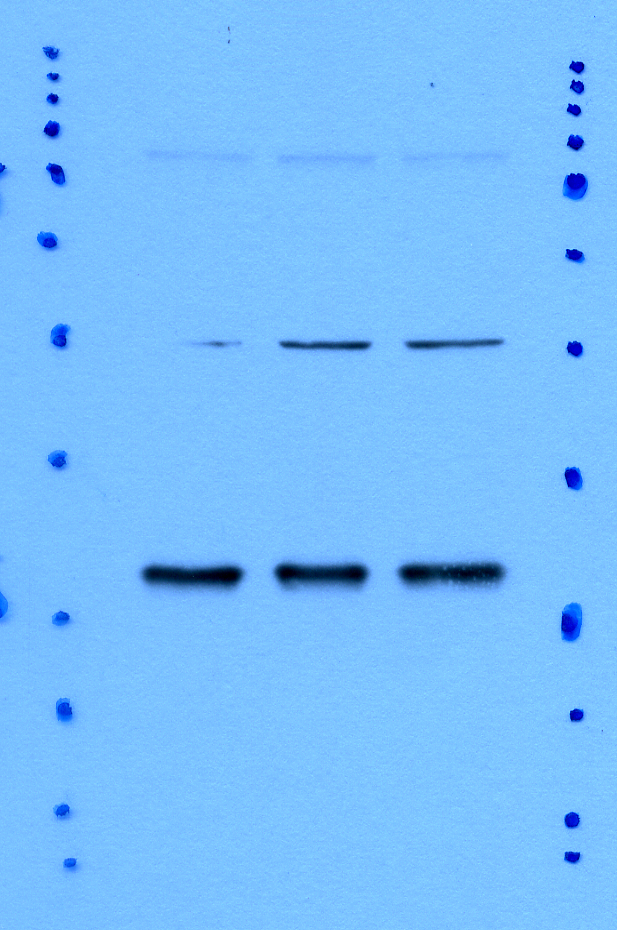

Supplement: Supplementary file 5 [file LSA-2024-02755_SdataF3.zip › LSA-2024-02755_SdataF3.13.tif]

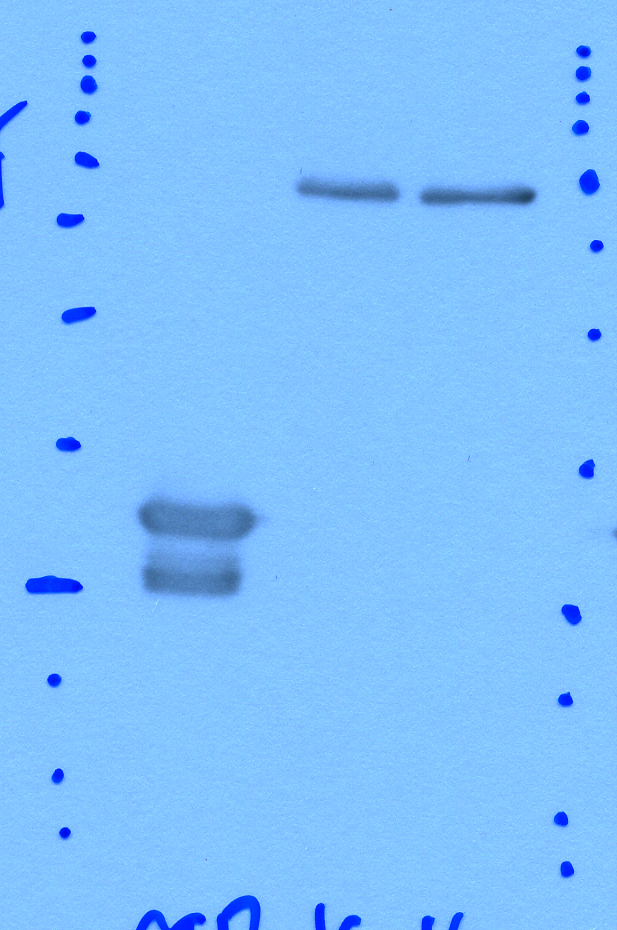

Supplement: Supplementary file 5 [file LSA-2024-02755_SdataF3.zip › LSA-2024-02755_SdataF3.14.tif]

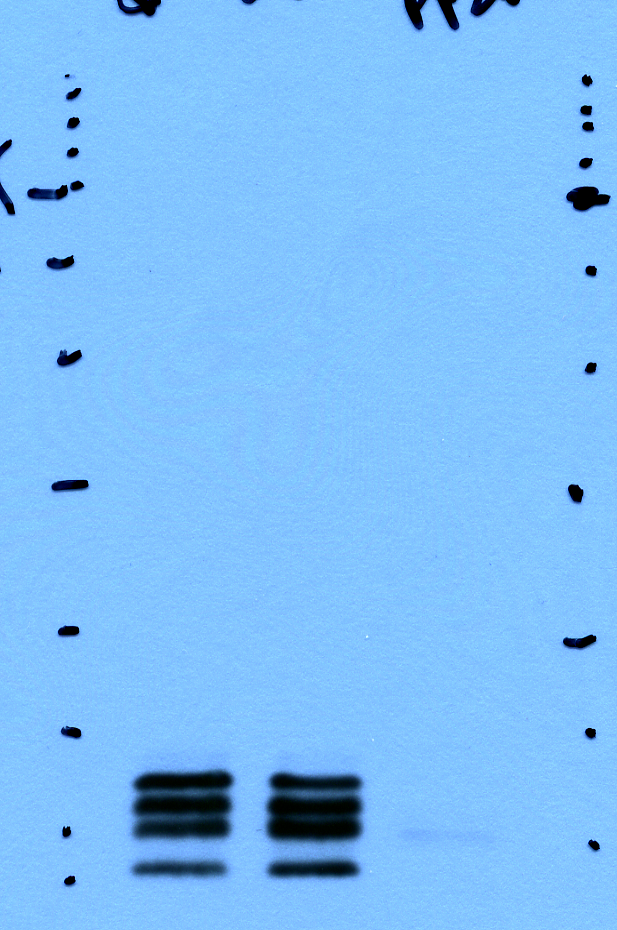

Supplement: Supplementary file 5 [file LSA-2024-02755_SdataF3.zip › LSA-2024-02755_SdataF3.15.tif]

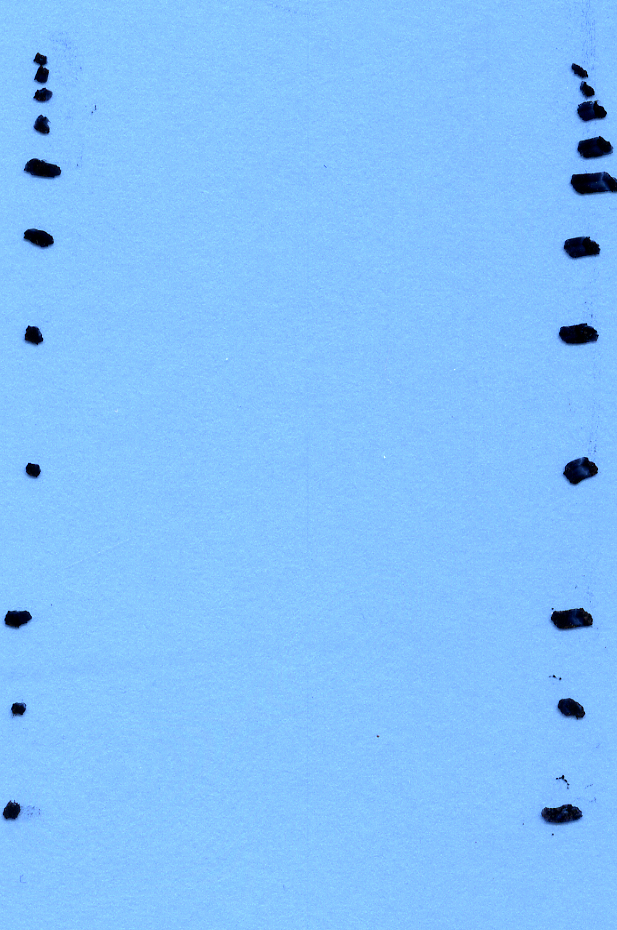

Supplement: Supplementary file 5 [file LSA-2024-02755_SdataF3.zip › LSA-2024-02755_SdataF3.16.tif]

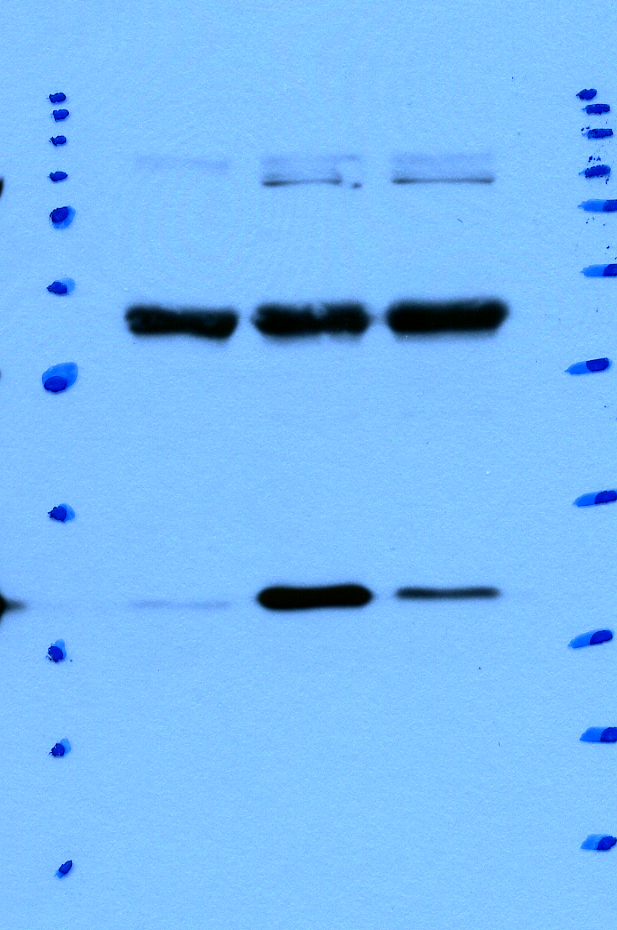

Supplement: Supplementary file 5 [file LSA-2024-02755_SdataF3.zip › LSA-2024-02755_SdataF3.17.tif]

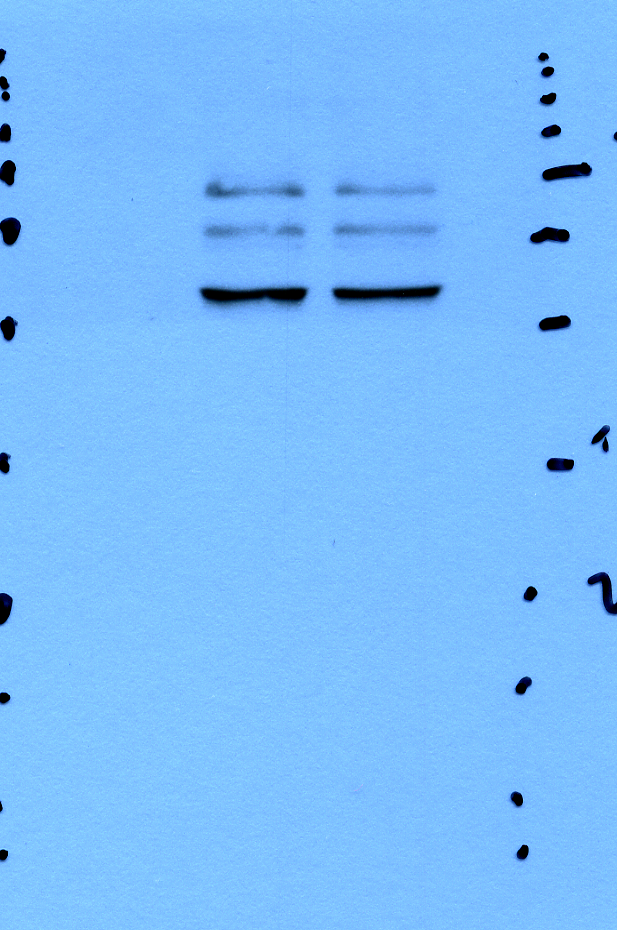

Supplement: Supplementary file 5 [file LSA-2024-02755_SdataF3.zip › LSA-2024-02755_SdataF3.18.tif]

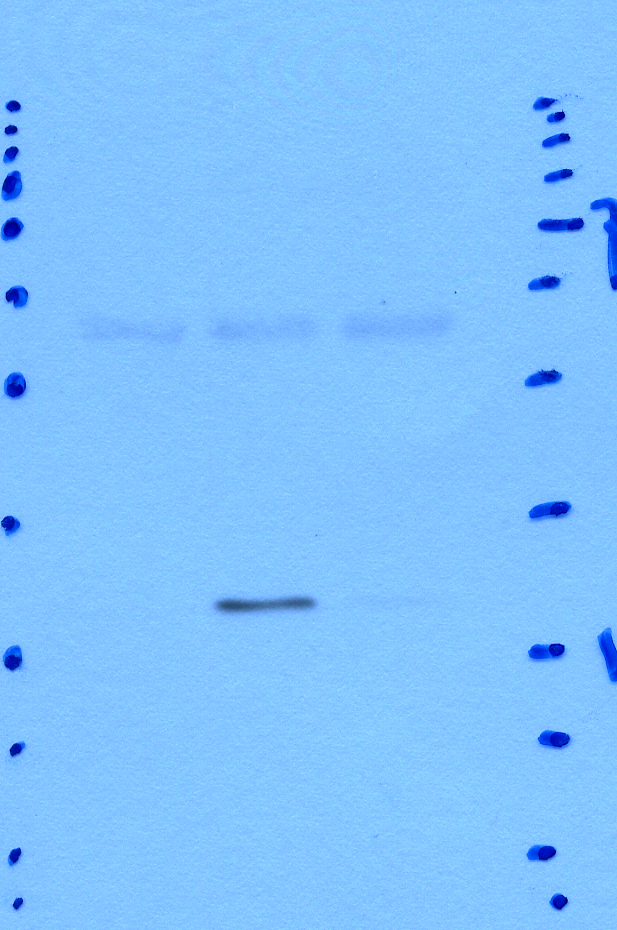

Supplement: Supplementary file 5 [file LSA-2024-02755_SdataF3.zip › LSA-2024-02755_SdataF3.19.tif]

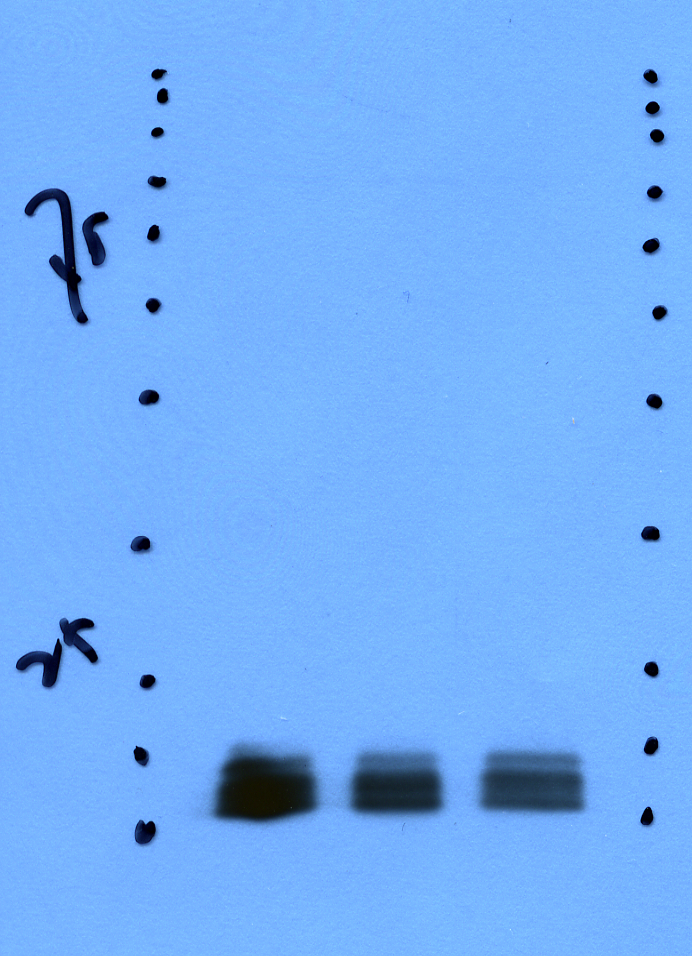

Supplement: Supplementary file 5 [file LSA-2024-02755_SdataF3.zip › LSA-2024-02755_SdataF3.2.tif]

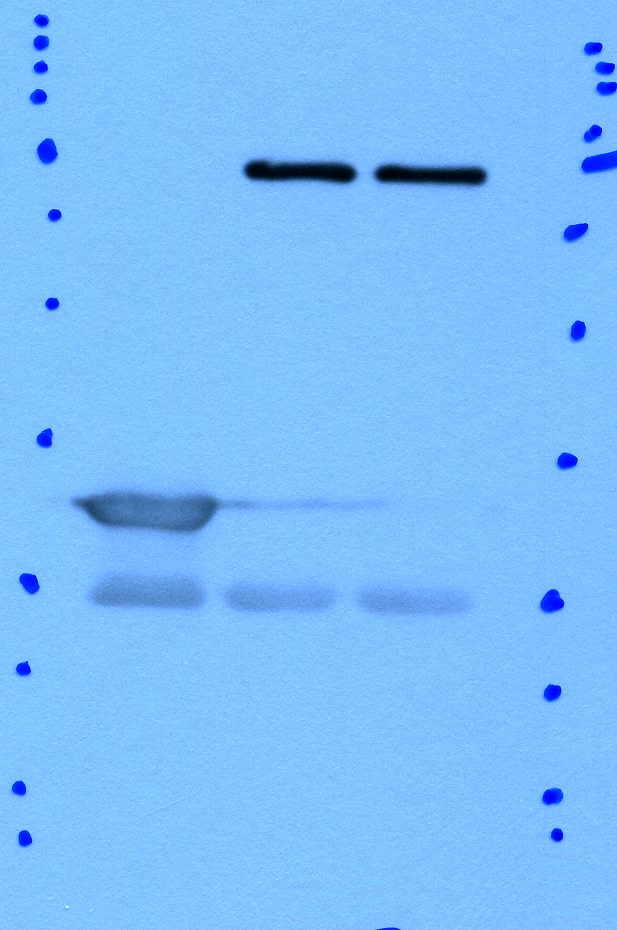

Supplement: Supplementary file 5 [file LSA-2024-02755_SdataF3.zip › LSA-2024-02755_SdataF3.20.tif]

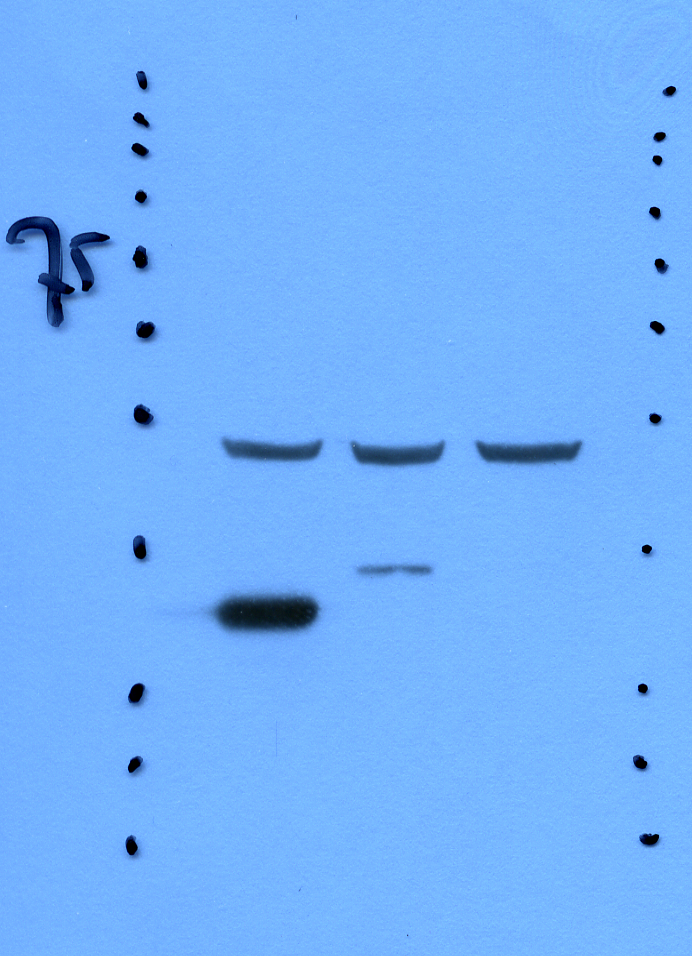

Supplement: Supplementary file 5 [file LSA-2024-02755_SdataF3.zip › LSA-2024-02755_SdataF3.3.tif]

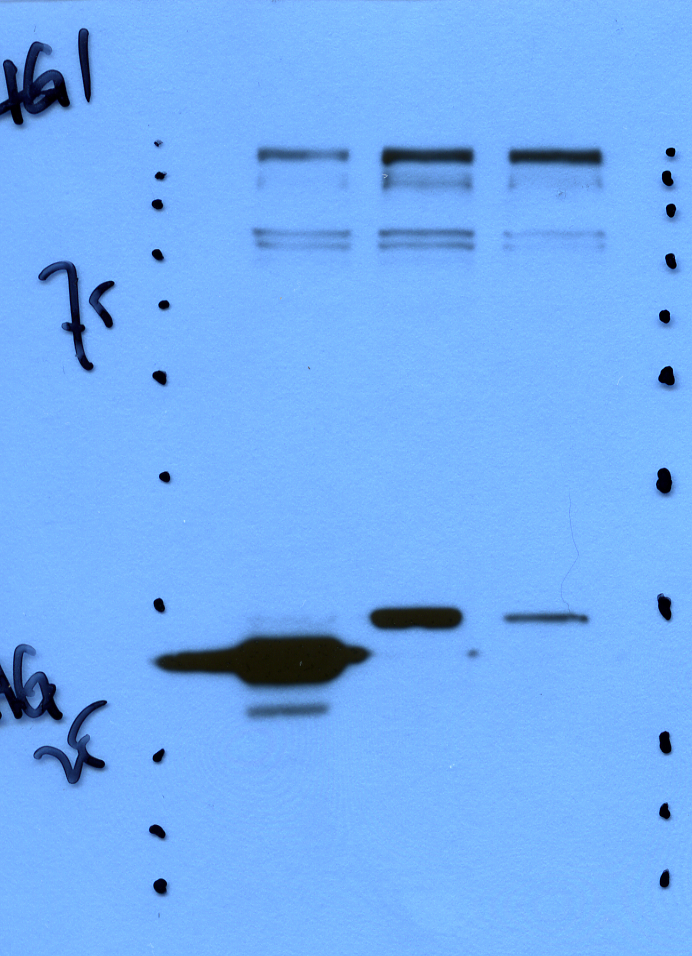

Supplement: Supplementary file 5 [file LSA-2024-02755_SdataF3.zip › LSA-2024-02755_SdataF3.4.tif]

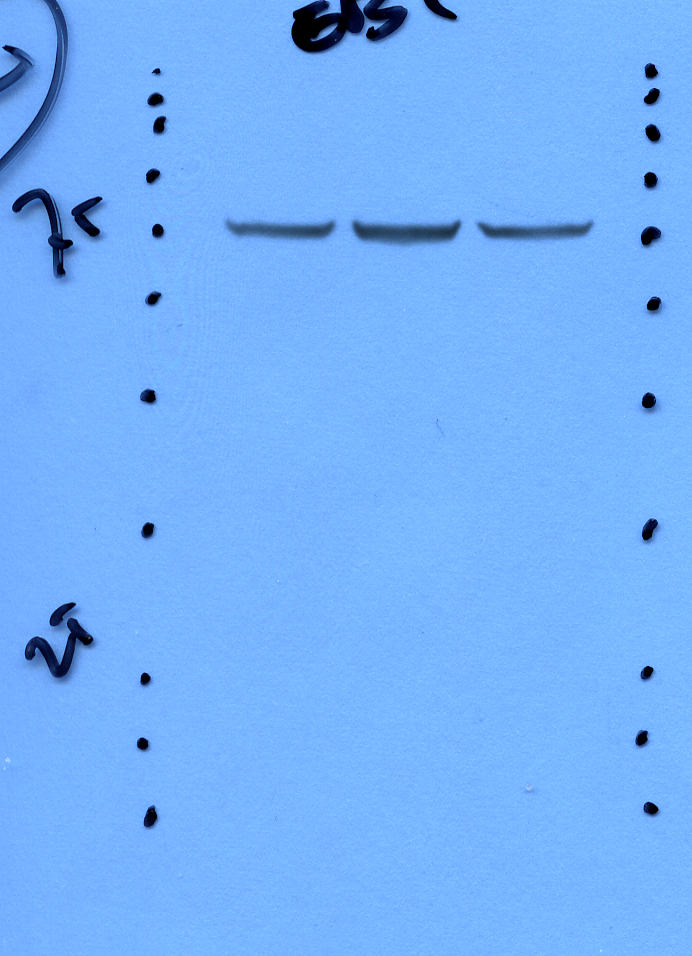

Supplement: Supplementary file 5 [file LSA-2024-02755_SdataF3.zip › LSA-2024-02755_SdataF3.5.tif]

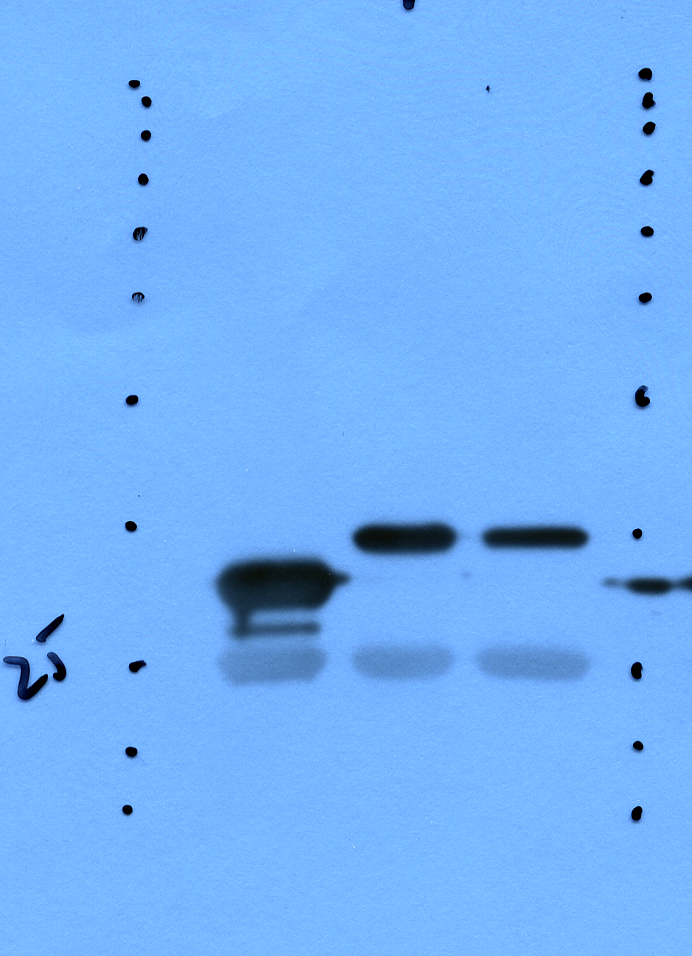

Supplement: Supplementary file 5 [file LSA-2024-02755_SdataF3.zip › LSA-2024-02755_SdataF3.6.tif]

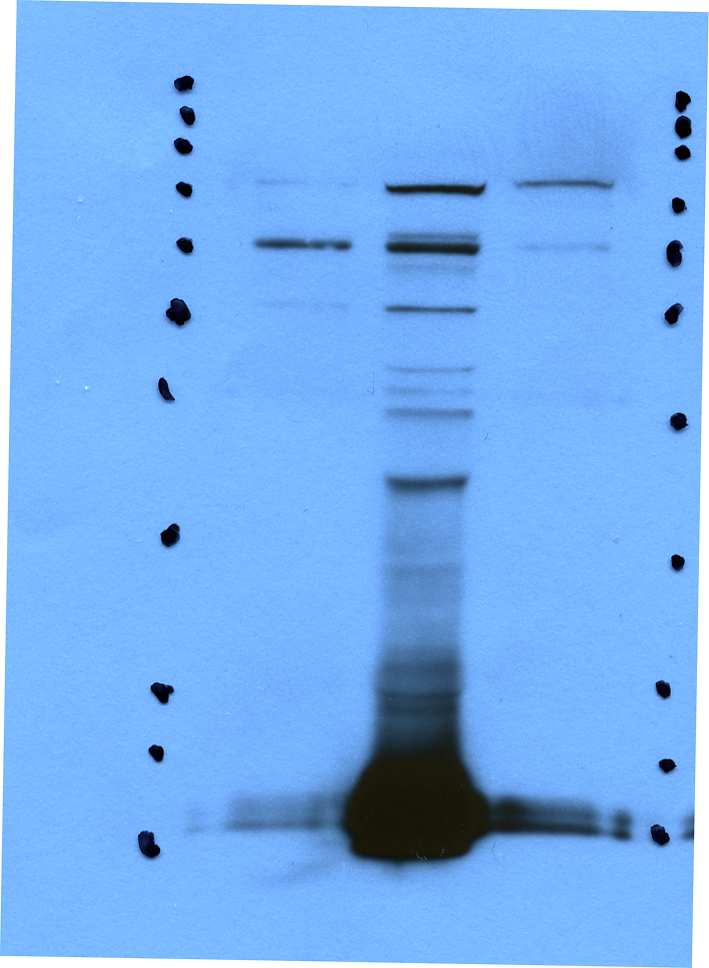

Supplement: Supplementary file 5 [file LSA-2024-02755_SdataF3.zip › LSA-2024-02755_SdataF3.7.tif]

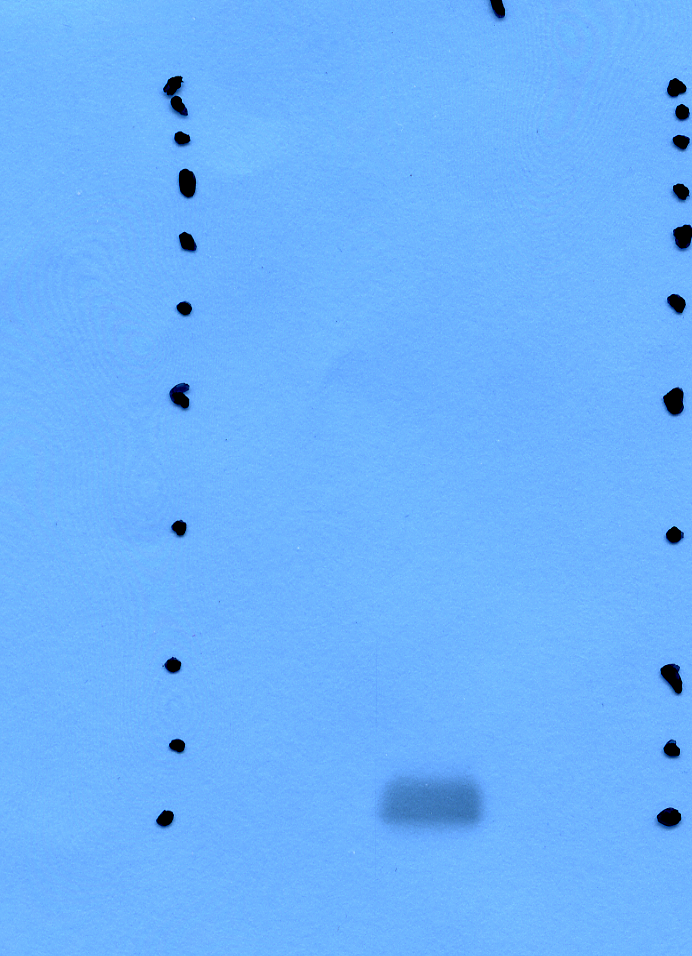

Supplement: Supplementary file 5 [file LSA-2024-02755_SdataF3.zip › LSA-2024-02755_SdataF3.8.tif]

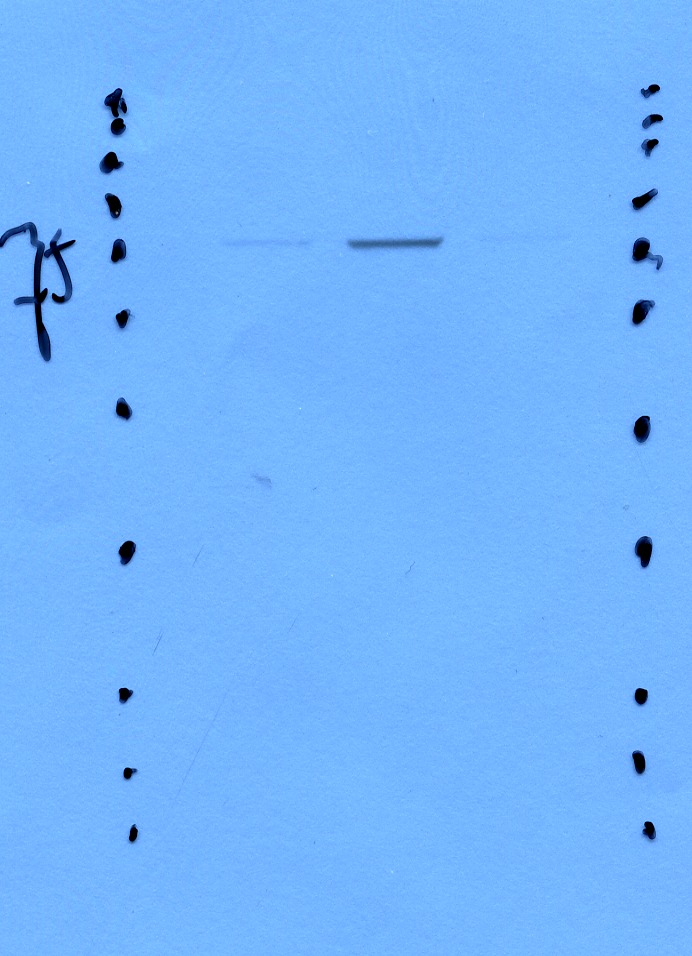

Supplement: Supplementary file 5 [file LSA-2024-02755_SdataF3.zip › LSA-2024-02755_SdataF3.9.tif]

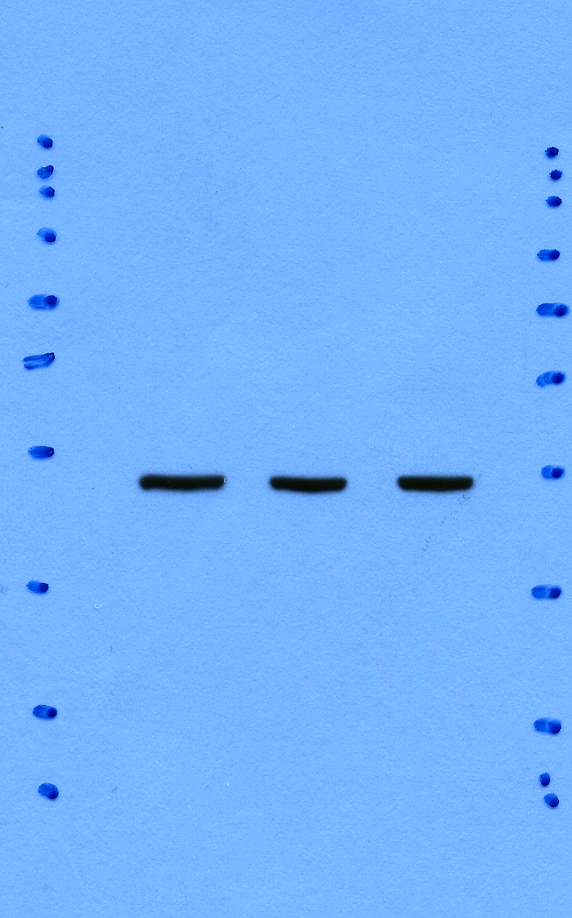

Supplement: Supplementary file 6 [file LSA-2024-02755_SdataF4.zip › LSA-2024-02755_SdataF4.1.tif]

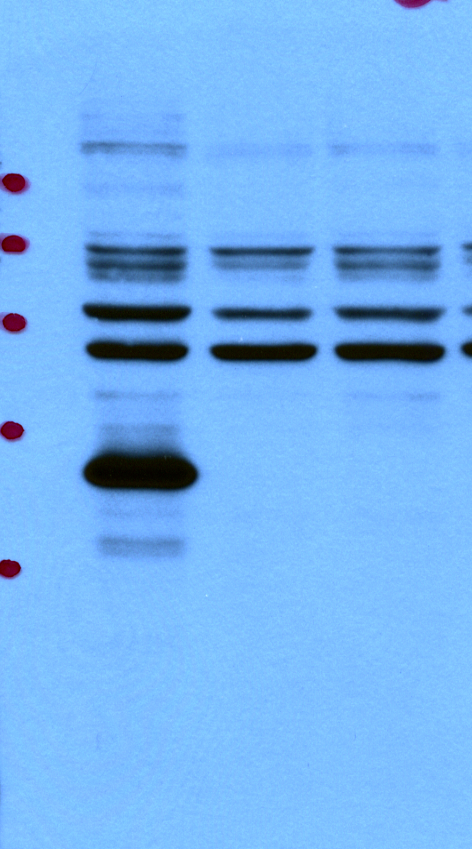

Supplement: Supplementary file 6 [file LSA-2024-02755_SdataF4.zip › LSA-2024-02755_SdataF4.10.tif]

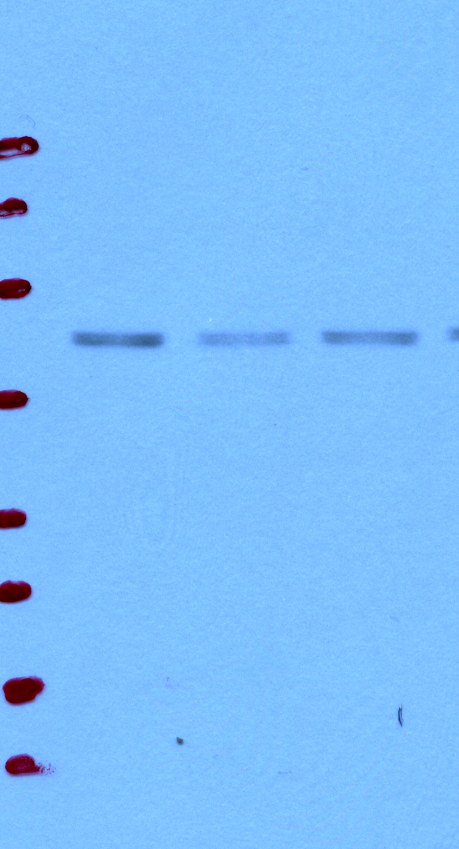

Supplement: Supplementary file 6 [file LSA-2024-02755_SdataF4.zip › LSA-2024-02755_SdataF4.11.tif]

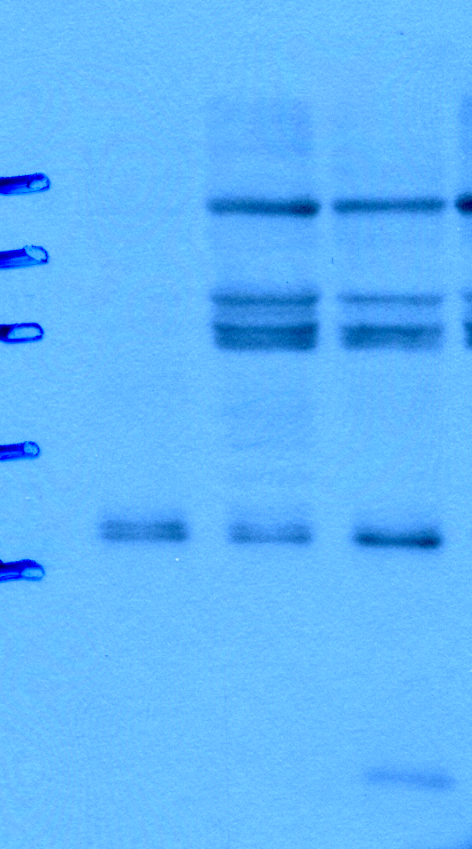

Supplement: Supplementary file 6 [file LSA-2024-02755_SdataF4.zip › LSA-2024-02755_SdataF4.12.tif]

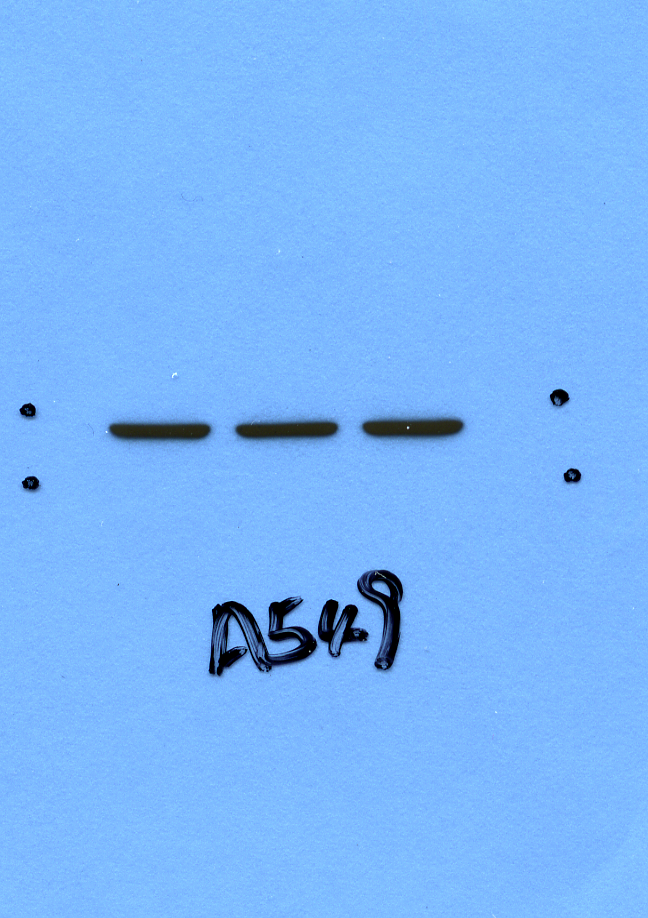

Supplement: Supplementary file 6 [file LSA-2024-02755_SdataF4.zip › LSA-2024-02755_SdataF4.13.tif]

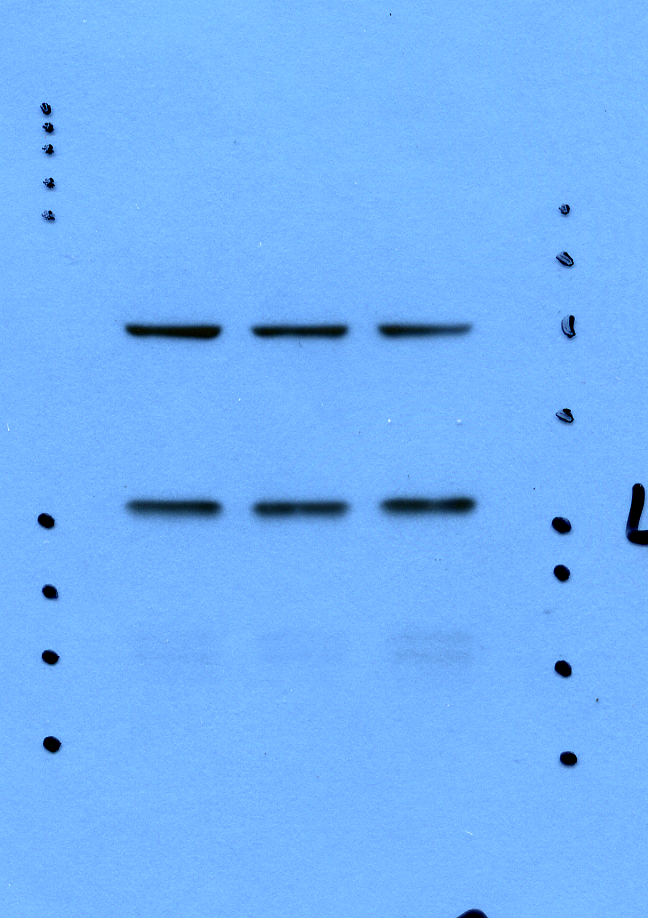

Supplement: Supplementary file 6 [file LSA-2024-02755_SdataF4.zip › LSA-2024-02755_SdataF4.14.tif]

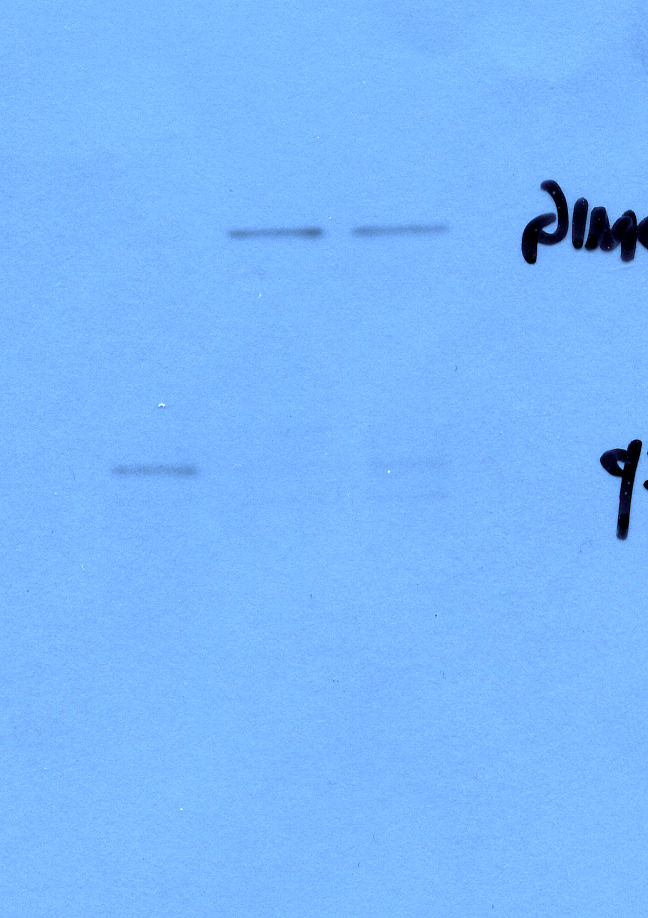

Supplement: Supplementary file 6 [file LSA-2024-02755_SdataF4.zip › LSA-2024-02755_SdataF4.15.tif]

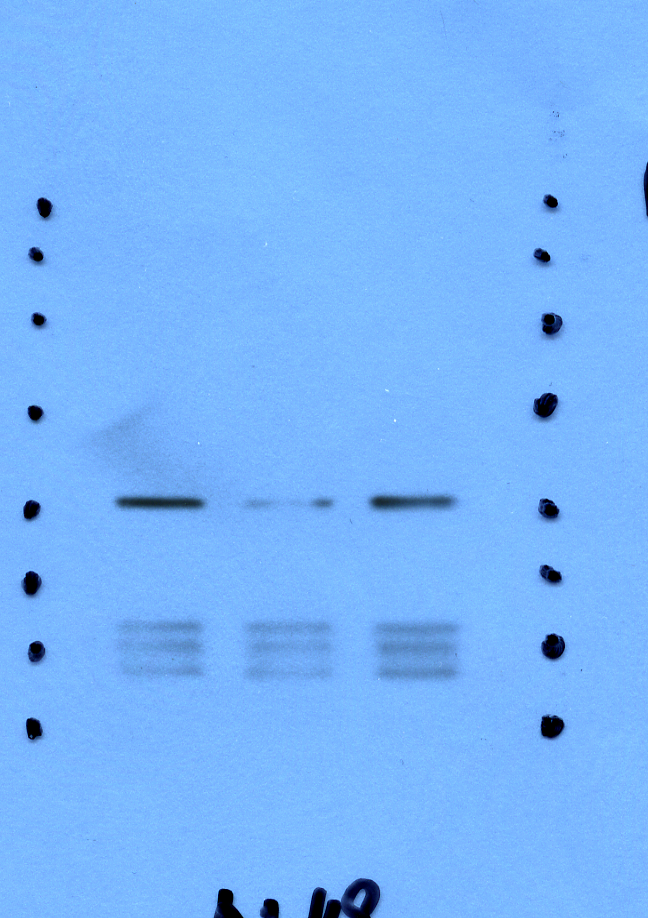

Supplement: Supplementary file 6 [file LSA-2024-02755_SdataF4.zip › LSA-2024-02755_SdataF4.16.tif]

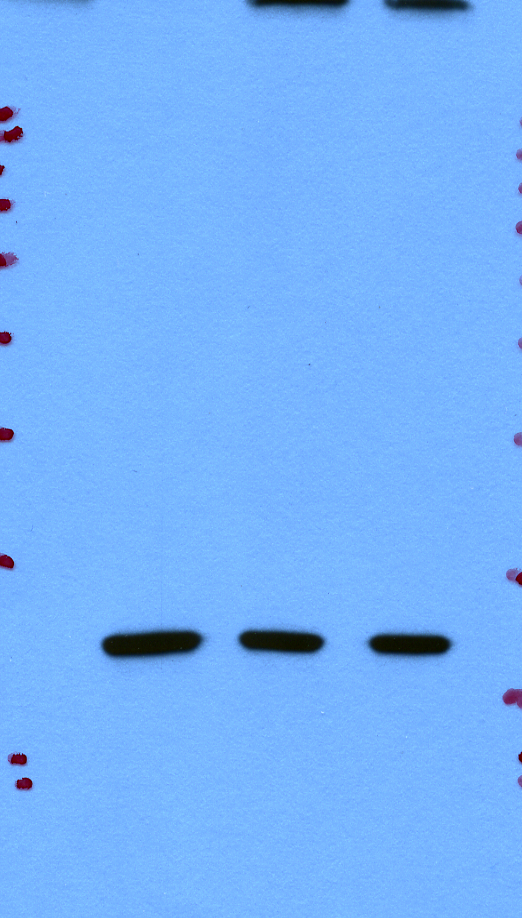

Supplement: Supplementary file 6 [file LSA-2024-02755_SdataF4.zip › LSA-2024-02755_SdataF4.2.tif]

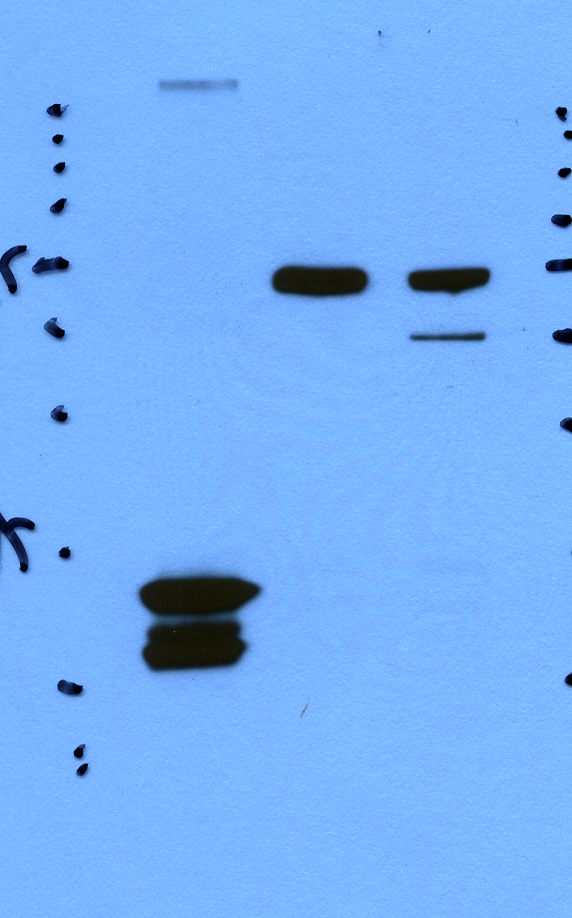

Supplement: Supplementary file 6 [file LSA-2024-02755_SdataF4.zip › LSA-2024-02755_SdataF4.3.tif]

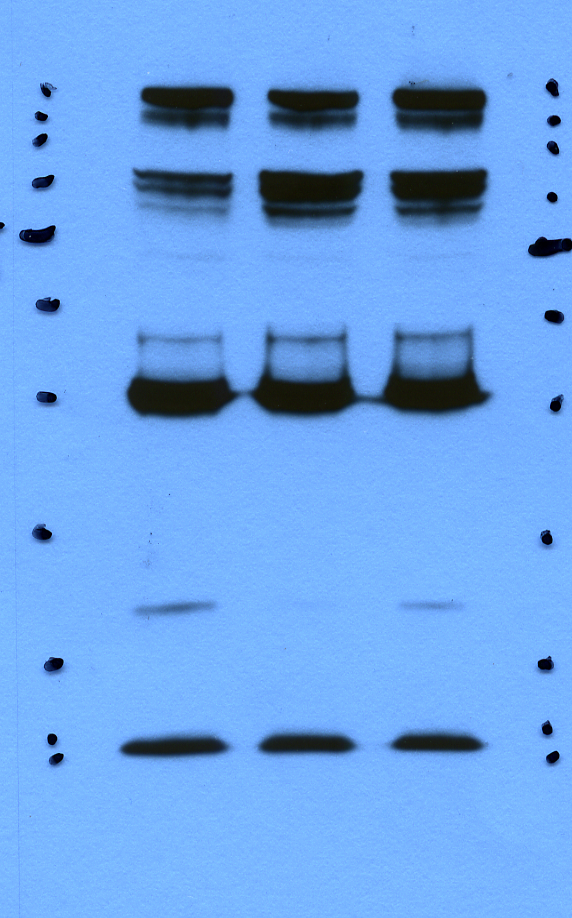

Supplement: Supplementary file 6 [file LSA-2024-02755_SdataF4.zip › LSA-2024-02755_SdataF4.4.tif]

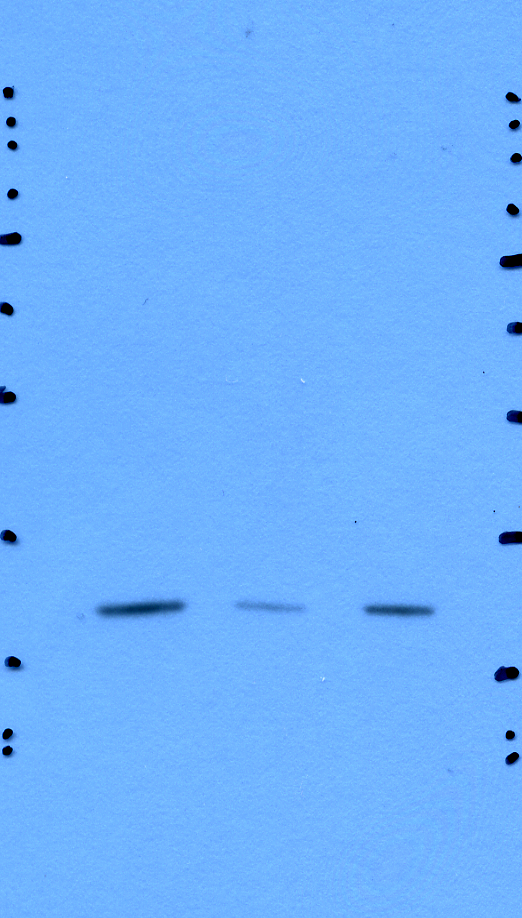

Supplement: Supplementary file 6 [file LSA-2024-02755_SdataF4.zip › LSA-2024-02755_SdataF4.5.tif]

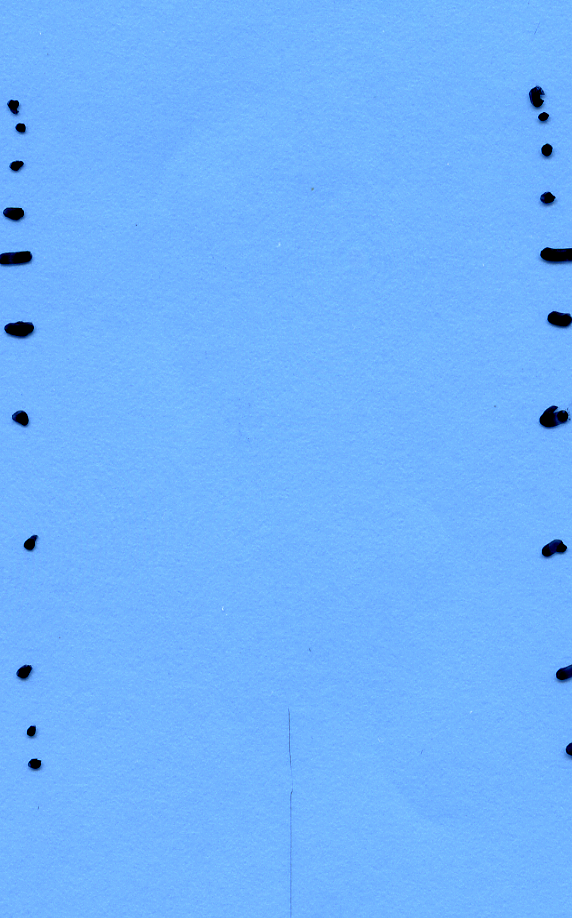

Supplement: Supplementary file 6 [file LSA-2024-02755_SdataF4.zip › LSA-2024-02755_SdataF4.6.tif]

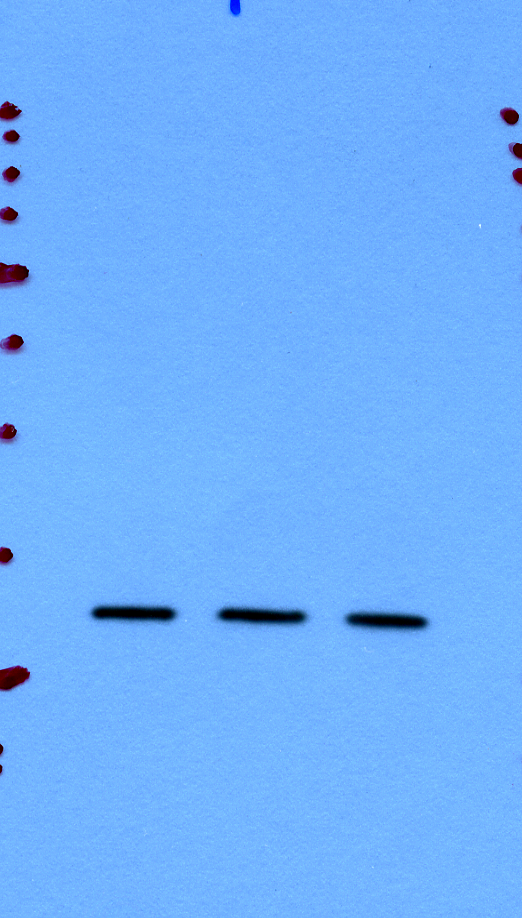

Supplement: Supplementary file 6 [file LSA-2024-02755_SdataF4.zip › LSA-2024-02755_SdataF4.7.tif]

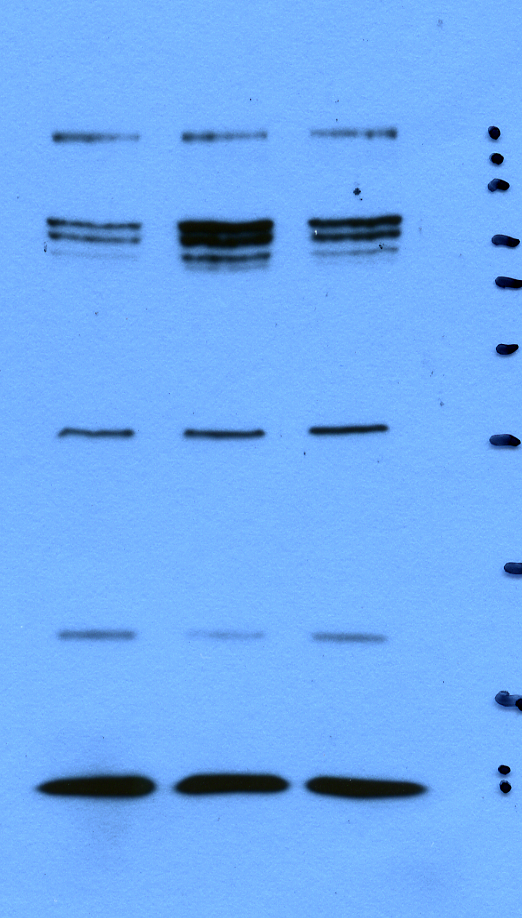

Supplement: Supplementary file 6 [file LSA-2024-02755_SdataF4.zip › LSA-2024-02755_SdataF4.8.tif]

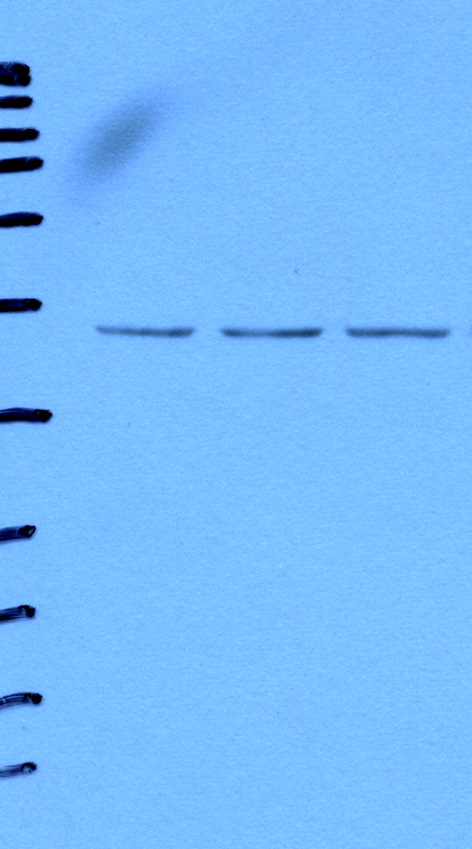

Supplement: Supplementary file 6 [file LSA-2024-02755_SdataF4.zip › LSA-2024-02755_SdataF4.9.tif]
